# Fast, flexible analysis of differences in cellular composition with *crumblr*

**DOI:** 10.1038/s41467-026-75681-7

**Published:** 2026-09-23

**Authors:** Gabriel E. Hoffman, Panos Roussos

**Affiliations:** 1 Center for Disease Neurogenomics, Icahn School of Medicine at Mount Sinai, New York, NY USA; 2 Department of Psychiatry, Icahn School of Medicine at Mount Sinai, New York, NY USA; 3 Department of Genetics and Genomic Sciences, Icahn School of Medicine at Mount Sinai, New York, NY USA; 4 Friedman Brain Institute, Icahn School of Medicine at Mount Sinai, New York, NY USA; 5 Center for Precision Medicine and Translational Therapeutics, James J. Peters VA Medical Center, Bronx, NY USA; 6 Mental Illness Research, Education and Clinical Centers, James J. Peters VA Medical Center, Bronx, NY USA

**Keywords:** Statistical methods, Software

## Abstract

Changes in cell type composition play an important role in human health and disease. Recent advances in single-cell technology have enabled the measurement of cell type composition at increasing cell lineage resolution across large cohorts of individuals. Yet this raises new challenges for statistical analysis of these compositional data to identify changes in cell type frequency. We introduce *crumblr* (DiseaseNeurogenomics.github.io/crumblr), a scalable statistical method for analyzing count ratio data using precision-weighted linear mixed models incorporating random effects for complex study designs. Uniquely, *crumblr* performs statistical testing at multiple levels of the cell lineage hierarchy using a multivariate approach to increase power over tests of one cell type. In simulations, *crumblr* increases power compared to existing methods while controlling the false positive rate. We demonstrate the application of *crumblr* to published single-cell RNA-seq datasets for aging, tuberculosis infection in T cells, bone metastases from prostate cancer, and SARS-CoV-2 infection.

## Abstract (web summary)

Statistical analysis of single-cell compositional data can be challenging. Here, Hoffman and Roussos develop the crumblr method and software to identify differences in cellular composition in single-cell datasets by incorporating the cell lineage hierarchy.

## Introduction

Tissues are composed of diverse cell types that play essential roles in human biology and disease^1^. Advances in single-cell technologies have enabled the measurement of genome-wide gene expression profiles from millions of single cells across hundreds of samples^2^. This has driven an improved understanding of gene expression dynamics within and across cell types, developmental stages, and disease states^3,4^. Cellular composition is also dynamic, and single-cell technologies have enabled studies of the changes in the frequency of different cell types due to early developmental stages, adult aging, immune response, and disease progression^5–9^.

As the scale of single-cell datasets continues to increase, study designs have become more complex, and the cell type resolution is expanding to consider lower-frequency cell types. Statistical tools should model these complex datasets, have the power to identify differences in cellular composition across samples, and control the false positive rate. Cell type frequency can vary due to biological factors of interest, but also due to tissue dissection, specimen quality, and technical factors. In a typical workflow, data-derived cell clusters are identified based on gene expression profiles, and the counts for each cell cluster are then analyzed with a regression model to test for changes in frequency across samples based on a variable of interest (i.e., disease state) while accounting for confounding variables.

Analyses take one of two approaches to examine changes in cellular composition, and the choice of approach affects statistical power, handling of complex study designs, integration with downstream analyses, and computational scaling. Regression models either use cell fractions (i.e., frequency) or model cell counts directly. While using cell fractions is simplest, computing a fraction from cell counts loses valuable information about the precision of the measurement. Consider two samples with an equal fraction of neuronal cells, but the first observation counts 1000 neurons out of 5000 total cells, while the second counts one neuron out of 5 total cells. Of course, 1000/5000 is a more precise measurement than 1/5 while having the same cell fraction. Similarly, measurements of lower-frequency cell types are less precise when the total number of cells observed is the same. Translating this intuition and lessons learned from modeling counts in RNA-seq data^10,11^ to the case of compositional data analysis, modeling the variation in measurement precision is essential to maximize statistical power when the total number of counts or the cell frequency varies widely across samples. Both of these cases are common in single-cell datasets.

The most widely used methods that analyze cell fractions are simple linear models using either observed cell fractions or some transformation of these fractions as the regression response. Linear models assume normally distributed errors, and this assumption is better satisfied after transforming the cell fractions using a log, logit, arcsin-sqrt, or the centered log ratio (CLR) transforms^12,13^. These methods are fast but sacrifice statistical power because they do not consider the precision of the cell fraction measurements. In addition, cell fractions can also be modeled directly with binomial or beta-binomial models to address this issue partially, but these also ignore the magnitude of the counts. Alternatively, methods that directly use count data and thus model variation in measurement precision include Poisson and negative binomial models, as well as the hierarchical Bayesian model of scCODA^14^. Yet these methods may not control the false positive rate across all conditions, can be computationally demanding, and don’t easily integrate into other downstream analyses.

Here, we introduce count ratio uncertainty modeling based linear regression (*crumblr*) for differential analysis of cellular composition that models variation in measurement precision, handles complex study designs with random effects, and performs tests at multiple levels of the cell lineage hierarchy using a multivariate approach. The *crumblr* framework has high statistical power, controls the false positive rate while being scalable to large single-cell datasets, and integrates with widely used R/Bioconductor packages.

## Results

### *crumblr* workflow for differential cellular composition analysis

**Fig. 1:**
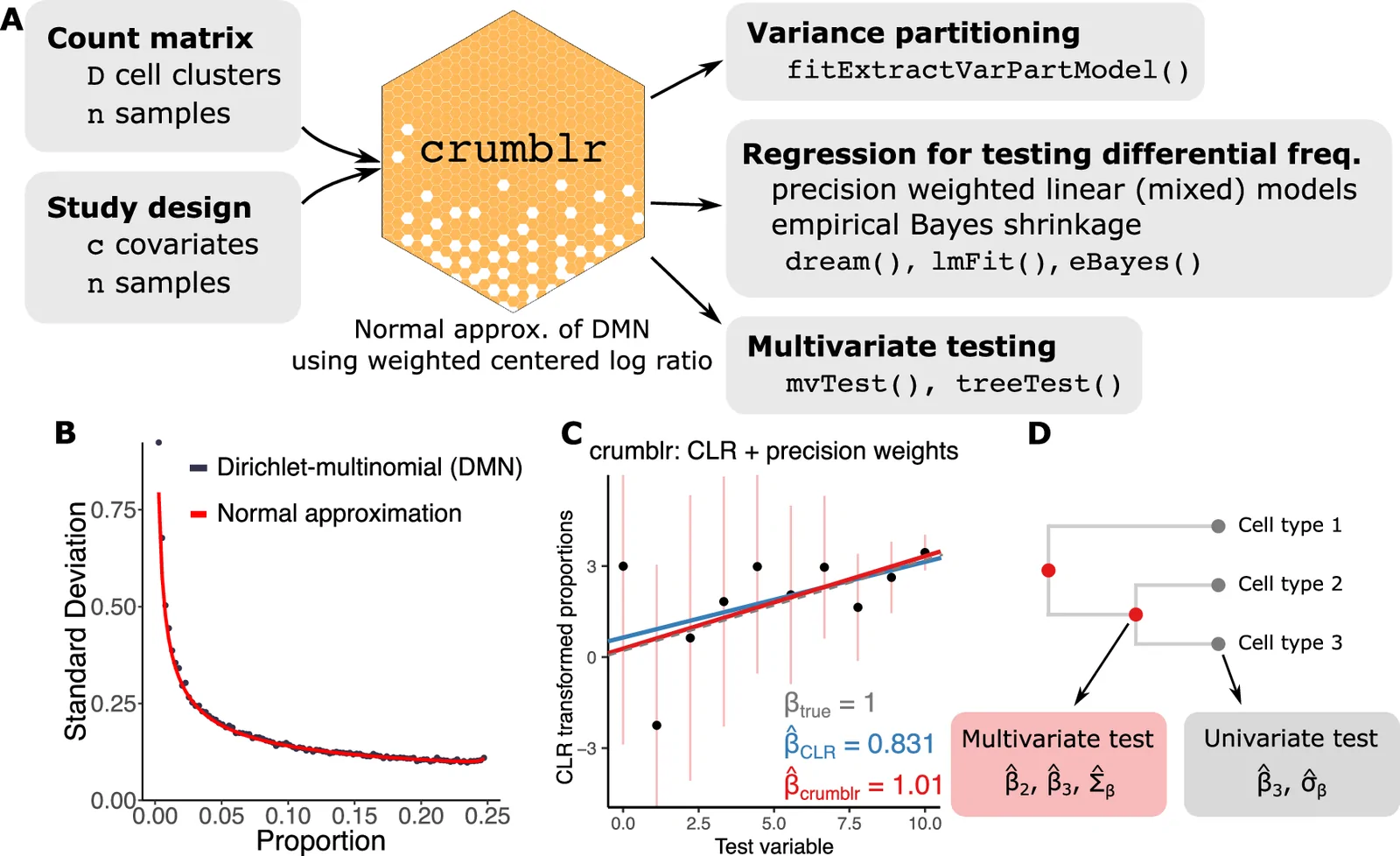
*crumblr* analysis workflow. **A** *crumblr* transformation of the count matrix enables precision weighting for variance partition analysis, linear mixed model testing for differential cell frequency, and multivariate testing along a cellular hierarchy. **B** Standard deviation of the CLR-transformed proportions from 1000 samples drawn from a Dirichlet-multinomial distribution (blue points) is compared to the normal approximation developed here (red line) when at least two counts are observed. Data was simulated using an overdispersion of τ = 10, 500 total counts across 15 categories and 1000 simulations. **C** Illustration showing the estimated coefficient estimated using standard regression fit on CLR-transformed proportions (blue line), and precision-weighted regression using *crumblr* (red line) that models the measurement uncertainty in the count ratios, shown here by error bars indicating 95% confidence intervals. **D** Multivariate hypothesis testing in *crumblr* enables analysis of internal nodes in a hierarchical clustering of cell types while modeling estimated effect sizes and the correlation between them.

The *crumblr* framework enables analysis of compositional data starting from cell cluster counts by transforming the count data and using weighted regression models for variance partitioning analysis, differential composition testing with univariate tests, and multivariate testing along a cellular hierarchy (Fig. 1A). The *crumblr* approach models observed cell counts following transformation with the CLR. The CLR transform is widely used in compositional data analysis and normalizes each cell component with the same denominator using the geometric mean of cell frequencies^12,15,16^. The CLR can be evaluated in log space as a linear combination (i.e., weighted sum) of the log proportions and transforms fractions for use as responses or covariates in regression models, as well as PCA and hierarchical clustering^12,15,16^. CLR can naturally be used in linear mixed models, allowing for random effects and multivariate testing.

Yet CLR transforms the cell *fractions* and does not consider the observed *counts* or the variation in measurement precision. The *crumblr* framework applies the CLR transform and uses an asymptotic normal approximation of the Dirichlet-multinomial distribution to estimate the sampling variance of the transformed fractions (see “Methods”). This approximation works well across a wide range of cell counts and proportions (Fig. 1B). These sample variances are used in precision-weighted regressions to model variation in measurement precision (Fig. 1C), as is widely used in differential expression analysis^10,17^. Transforming compositional data analysis into a precision-weighted regression model enables the incorporation of random effects and empirical Bayes shrinkage of test statistics. In addition to univariate testing of each cell cluster, precision-weighted models enable testing at multiple levels of the cell lineage hierarchy using a multivariate approach to increase power over tests of one cell cluster. Compared to 12 other methods for univariate testing of changes in cellular composition, *crumblr* matches the statistical performance of negative binomial methods, having the highest statistical power. Yet of these, only *crumblr* controls the false positive rate across conditions under a range of conditions, has the flexibility to incorporate random effects, and enables multivariate testing while being scalable to large datasets (Supplementary Fig. 1). The *crumblr* workflow easily integrates with widely used R/Bioconductor packages.

### Performance on simulated data

**Fig. 2:**
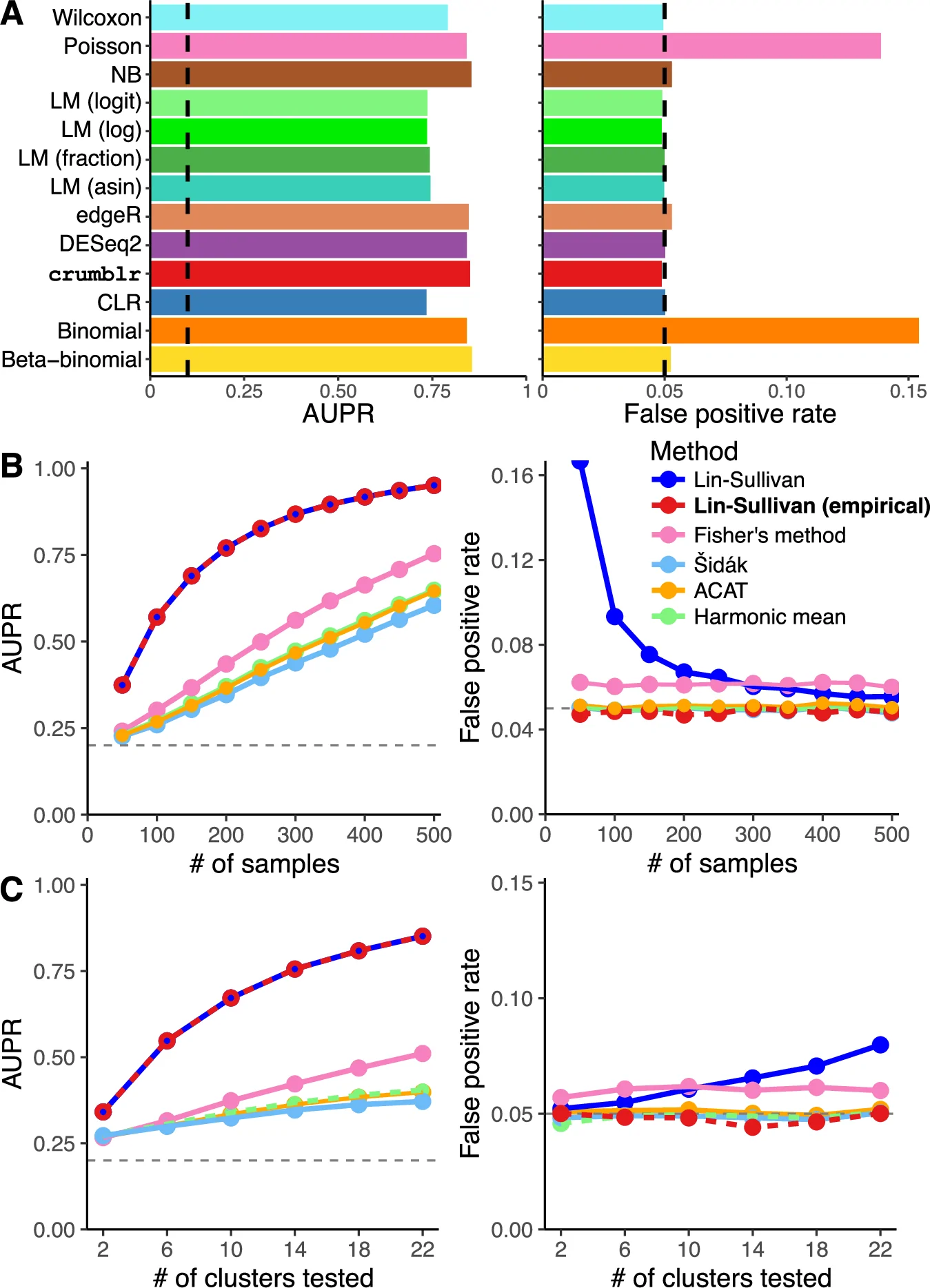
Performance on simulated cellar composition data. **A** Performance of univariate tests across 10k simulated datasets under baseline parameters. For linear models indicated with “LM”, “fraction” indicates using the cell fraction, f, as the response, “log” indicates log(f), “logit” indicates ${{\mbox{logit}}}(f)\,=\,\log (f)\,-\,\log (1-f)$, and “asin” indicates $\arcsin (\sqrt{f})$. The area under the precision-recall curve (AUPR) (left) and false positive rate (FPR) at a 5% cutoff computed under the null model (right). For AUPR, the dashed line indicates the performance of the random method. For FPR, the dashed line indicates the target for a properly calibrated method. **B** Performance of multivariate testing in simulated data for increasing sample sizes. AUPR (left) and FPR (right) of six methods for multivariate testing. **C** Performance of multivariate testing in simulated data for an increasing number of cell clusters tested. AUPR (left) and FPR (right).

We evaluated the performance of *crumblr* and 10 other methods to identify differences in cell frequencies in simulations across a broad range of conditions. Count data were drawn from a Dirichlet-multinomial distribution, and baseline simulations were performed with 200 samples, a variable of interest drawn from a standard normal distribution, 10 cell clusters, of which one had differential frequency with an effect size of 0.2, a mean of 2000 cells observed for each sample, no batch effect, an overdispersion parameter of 10, and 10 K simulated datasets. The *crumblr* and negative binomial models showed the highest area under the precision-recall (AUPR) curve to identify the cell clusters with a change in frequency driven by the simulated variable of interest (Fig. 2A). Methods that did not model the measurement precision (i.e., linear regression on fraction, log fraction, logit fraction, arcsin-sqrt fraction, or CLR) had lower power, and methods that did not model overdispersion of count data (i.e., Poisson and binomial models) did not control the false positive rate. Further simulations modified the parameters of this baseline simulation by varying the total cell counts between 100 and 4000 (Supplementary Fig. 2), number of cell clusters between 7 and 20 (Supplementary Fig. 3), sample size between 20 and 200 (Supplementary Fig. 4), count overdispersion parameter between 1 and 20 (Supplementary Fig. 5), and variance explained by batch effect between 0 and 20% (Supplementary Fig. 6). Across all these conditions, *crumblr* and the negative binomial model consistently showed the highest power, while *crumblr* controlled the false positive rate even for small sample sizes. Both *crumblr* and the negative binomial model were fast and required <10 s to analyze 500 samples with 20 cell clusters (Supplementary Fig. 7). Rigorous analysis with scCODA^14^ was limited by its long run time (~1000 s on this dataset), and evaluation on 100 simulated datasets showed lower statistical power than *crumblr* using default settings (Supplementary Fig. 8).

In addition to performing univariate testing of each cell cluster individually, *crumblr* can perform multivariate testing along the cell lineage hierarchy to test for changes in cellular composition at multiple resolutions. In a hierarchy, testing an internal node with *m* children corresponds to a multivariate test of the *m* child cell clusters. We evaluated the performance of 3 existing methods to perform multivariate testing by combining the results for univariate tests from *crumblr*. Based on these simulations, varying the number of samples (Fig. 2B) and the cell clusters *m* (Fig. 2C), we developed a statistical test with high power that controls the false positive rate.

Of these multivariate methods, the Šidák method is the simplest and reports the smallest *P* value corrected for the number of clusters tested. This method had low power because it uses only the smallest *P* value. We also consider Fisher’s method that combines *P* values from all tests and compares a test statistic to a chi-squared null distribution, the aggregated Cauchy association test that transforms the *P* values and compares a test statistic to a Cauchy distribution^18^, and the harmonic mean of the *P* values^19^. Yet these methods do not consider the effect size or model the known dependency structure between tests, resulting in lower power. Instead, we use a fixed effect meta-analysis of the estimated effect size while modeling the correlation in the estimates using the Lin-Sullivan test^20^. This approach substantially increases the power to find sets of cell clusters with differential frequency, but for small sample sizes, it gave an elevated false positive rate since it depends on an asymptotic null distribution. We developed an extension to the Lin-Sullivan test using an empirical null distribution that controls the false positive rate for small sample sizes while retaining high power (see “Methods”).

### Cell type composition changes with age in PBMCs

**Fig. 3:**
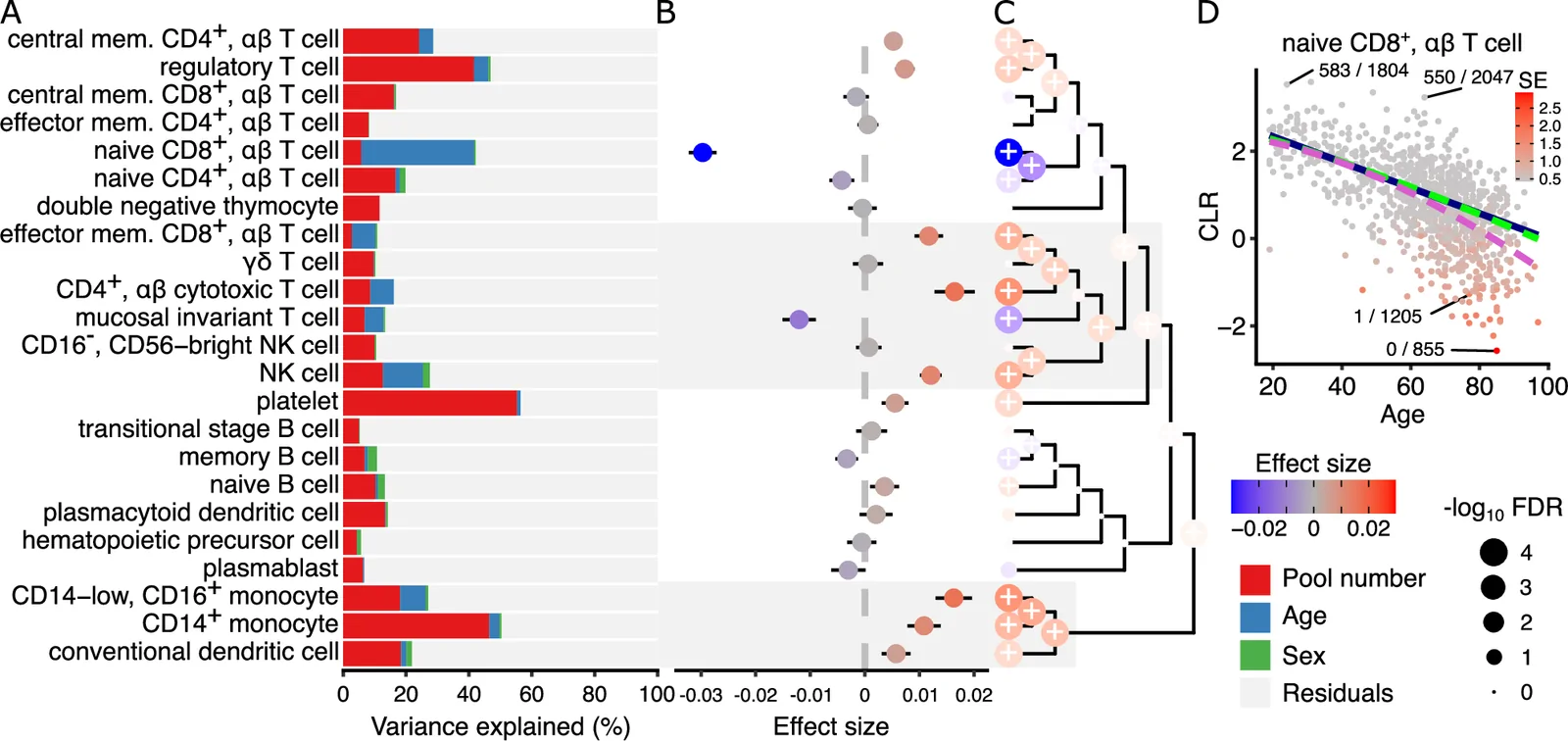
*crumblr* identifies compositional changes associated with aging in blood. **A** Variance partitioning analysis quantifies the contribution of each variable to variation in frequency for each cell type. **B** Estimated effect size from regression analysis of composition associated with donor age. A positive coefficient estimate indicates increased cell type frequency with age. The color indicates effect size, and error bars indicate 95% confidence interval. **C** Hierarchical clustering of cell types with colored points indicating estimated effect sizes for each cell type at leaves and multivariate testing at internal nodes. Results on leaves match results in (**B**). Point size indicates FDR, and ‘+’ indicates FDR < 5%. **D** Scatter plot of CLR-transformed naive CD8^+^ αβ T-cell frequency versus donor age. A point indicates a single donor, and the color indicates the standard error from limited measurement precision. A subset of points is labeled with the observed cell type fraction with, for example, 1/1205 indicating 1 CD8^+^ αβ T cell out of 1205 total cells. Regression trends are shown for a linear fit (blue) and quadratic fit (green) modeling the varying measurement precision using *crumblr*, and a quadratic fit (purple) ignoring measurement precision.

The biology of the human immune system depends on age, with changes in both gene expression programs and cellular composition over time^21^. However, a detailed understanding of the dynamics of high-resolution cell types has been limited by the ability to measure cellular composition across a cohort with a broad age range. Yazar et al.^22^ generated single-cell transcriptome data for 1.2 M peripheral blood mononuclear cells (PBMCs) from 982 donors in the OneK1K cohort with ages ranging from 19 to 97 years, and identified 29 data-derived clusters with 23 having at least 1000 cells. Variance partitioning analysis showed substantial variation in cell type frequency across the 75 batches, but little variation between males and females (Fig. 3A). Age explained 35.9% of the variation in frequency for naive CD8^+^ αβ T cells and greater than 5% for five other cell clusters. Univariate analysis identified the strongest decreases in frequency of CD8^+^ αβ T cells (*β* = −0.0297, *P* = 3.6e-93, FDR <1e-10) and mucosal invariant T cells (*β* = −0.0120, *P* = 2.8e-14, FDR = <1e-10) with age, along with significant decreases in two additional clusters and significant increases in ten clusters (Fig. 3B). Hierarchical clustering of gene expression profiles and multivariate testing of cellular composition shows widespread changes in immune cell frequency throughout human aging (Fig. 3C). Examining the trajectory of CD8^+^ αβ T-cell frequency shows a marked decrease over age, and also highlights the importance of modeling the measurement error with *crumblr* (Fig. 3D). Since the frequency of CD8^+^ αβ T cells decreases with age, measurement precision also decreases with age. While the *crumblr* model supports a linear trend between transformed frequency and age, a model ignoring the varying measurement precision supports a non-linear trend with an accelerated decrease in frequency after age ~70 (*P* = 5.20e-3).

T-cell populations are dynamic and known to change over the course of aging; our findings are consistent with previous studies. Thymic involution associated with aging results in decreased generation of naive T cells and, in particular, causes a marked decrease in naive CD8^+^ T cells in peripheral blood, a modest decrease in naive CD4^+^ T cells, and a compensatory increase in memory T cells^23,24^. Indeed, we observe a large decrease in naive CD8^+^ αβ T cells, a minimal change in naive CD4^+^ αβ T cells, and an increase in effector memory CD8+ αβ T cells and CD4^+^ αβ cytotoxic memory T cells. The loss of CD8^+^ T cells with age is a key factor in immunosenescence, resulting in a reduced ability to fight off infections and a reduced response to vaccination^23–25^.

### Decrease in CD4^+^ T_h_1 and T_h_17 T cells following tuberculosis

The immune response to infection can alter cellular composition at multiple levels. Nathan et al.^26^ studied the steady state of T cells following *Mycobacterium tuberculosis* (*M.* *tb*) infection and disease resolution, by isolating 500K T cells from 259 donors, including 128 who had progressed to active tuberculosis (TB) after infection. After isolating T cells from PBMCs and applying CITE-seq to measure gene expression and surface proteins for each cell, their analysis identified 31 data-derived T-cell clusters. The average frequency of these T-cell clusters ranged from 0.062 to 8.6%, with the frequency of each cluster varying by over tenfold across individuals (Supplementary Fig. 9). The original analysis found that the frequency of some clusters correlated with age, sex, ancestry, and the season of the blood draw and identified a cluster of CD4^+^ T_h_17 T cells showing a significant decrease in frequency in individuals who progressed to active TB after infection.

**Fig. 4:**
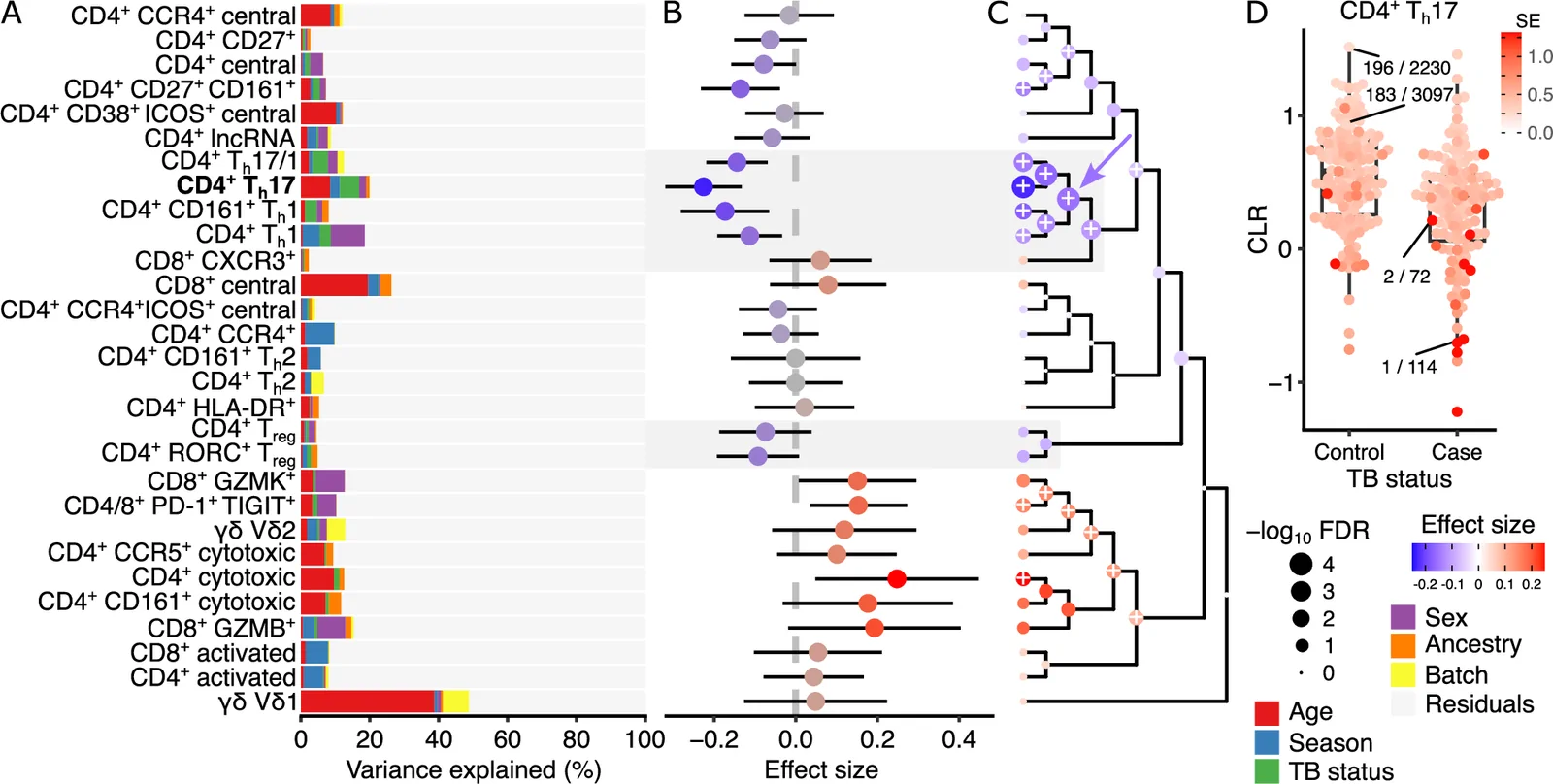
*crumblr* identifies compositional changes associated with tuberculosis infection in T-cell subpopulations in 259 donors. **A** Variance partitioning analysis quantifies the contribution of each variable to variation in frequency for each cell cluster. **B** Estimated effect size by comparing the composition of TB-inflected and controls. A positive coefficient estimate indicates an increase in the frequency of TB infection. Color indicates effect size, and error bars indicate a 95% confidence interval. **C** Hierarchical clustering of cell types with colored points indicating estimated effect sizes for each cell type at leaves and multivariate testing at internal nodes. Results on leaves match results in (**B**). Point size indicates FDR, “+” indicates FDR < 5%, and error bars indicate 95% confidence intervals. The parent node mentioned in the text is indicated by the blue arrow. **D** Transformed frequency of CD4^+^ T_h_17 T cells in healthy controls and donors with previous TB infection. A point indicates a single donor, and the color indicates the standard error from limited measurement precision using *crumblr*. A subset of points is labeled with the observed cell type fraction with, for example, 1/144, indicating 1 CD4^+^ T_h_17 T cell out of 114 total cells. Boxes indicate mean, first and third quartiles, whiskers indicate 1.5 interquartile range.

We applied *crumblr* to identify changes in cell type composition associated with TB status for each of the 31 data-derived T-cell clusters, as well as hierarchical clusters. Variance partitioning analysis showed the substantial contribution of age to T-cell composition, while season and sex explained less variation (Fig. 4A). Univariate analysis of differential cell frequency based on TB status identified the same CD4^+^ T_h_17 cluster as having the strongest estimated effect (*β* = −0.226, *P *= 3.73e-6, FDR = 0.00011), but also identified three other T_h_1 or T_h_17 clusters with significant decreases in TB individuals (Fig. 4B). In addition, CD4^+^ cytotoxic T cells show the largest increases in frequency. Hierarchical clustering of the annotated cell types based on gene expression placed these the 4 T_h_1/T_h_17 cell clusters together, and multivariate differential frequency analysis showed that the parent node of these cell types had significantly decreased frequency following progression to active TB (*β* = −0.152, *P* = 3.04e-6, FDR = 1.06e-4) (Fig. 4C). Highlighting the CD4^+^ T_h_17 cluster shows the decrease in frequency following active TB, and also shows that samples with low frequency of this cell type also have high measurement error (Fig. 4D).

The finding of a decreased CD4^+^ T_h_17 frequency was then replicated in independent cohorts^26,27^. Although a low CD4^+^ T_h_17 frequency may precede progression to TB and thus increase susceptibility^26^, here we identify three additional T-cell clusters whose frequency covaries with CD4^+^ T_h_17 and also shows decreased frequency in TB progressors. In addition, we observe an increase in the frequency of CD4^+^ cytotoxic T cells, consistent with increased cytotoxicity of CD4^+^ T cells following active TB^28^.

### Cell type composition in bone metastases from prostate cancer

**Fig. 5:**
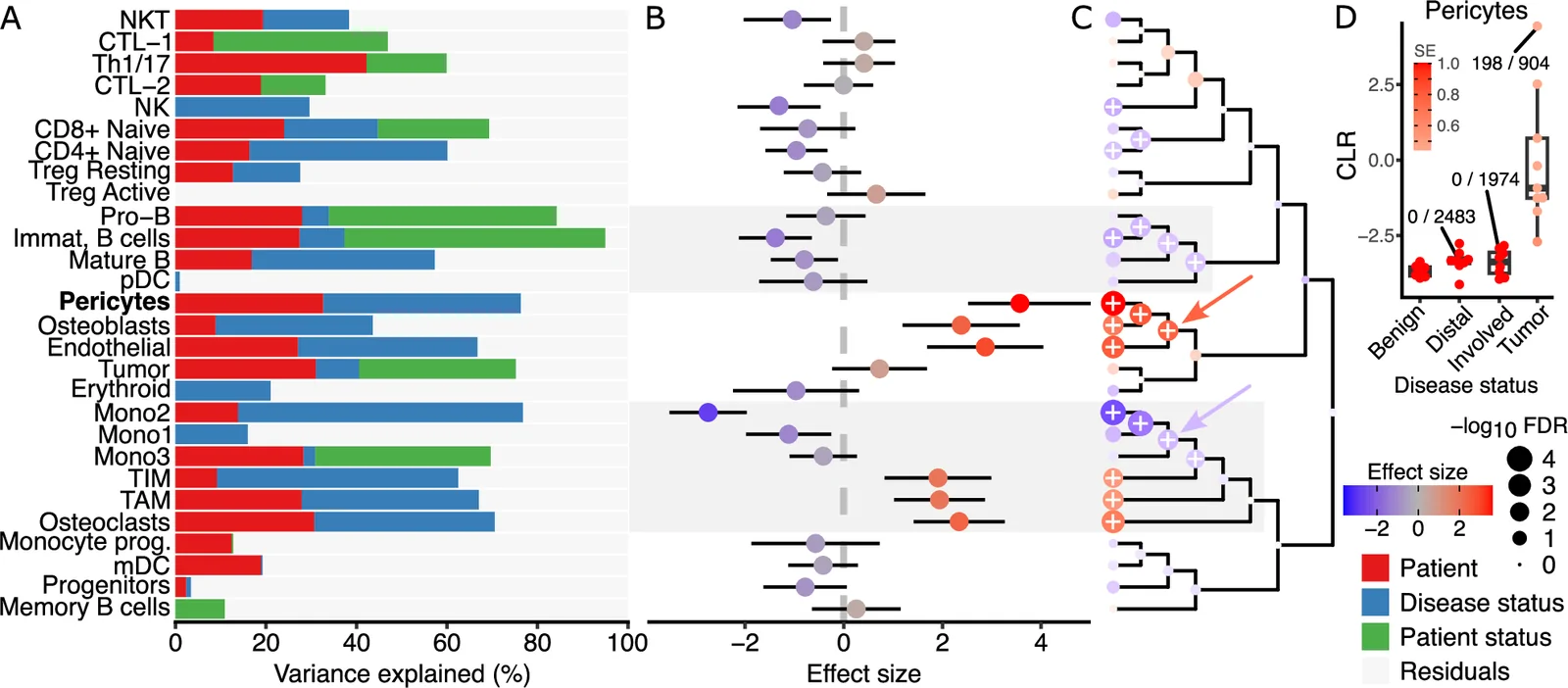
*crumblr* identifies composition changes in bone metastases from prostate cancer. **A** Variance partitioning analysis quantifies the contribution of each variable to variation in frequency for each cell type. **B** Estimated effect size by comparing the composition of solid tumors and involved bone marrow. The color indicates effect size, and error bars indicate a 95% confidence interval. **C** Hierarchical clustering of cell types with colored points indicating estimated effect sizes for each cell type at leaves and multivariate testing at internal nodes. Results on leaves match results in (**B**). Point size indicates FDR, and “+” indicates FDR < 5%. Parent nodes mentioned in the text are indicated by red and blue arrows. **D** Transformed frequency of pericytes for each disease state. Each point indicates a single sample, and the color indicates the standard error from limited measurement precision using *crumblr*. A subset of points is labeled with the observed cell type fraction with, for example, 198/904, indicating 198 pericytes out of 904 total cells. Boxes indicate mean, first and third quartiles, whiskers indicate 1.5 interquartile range.

Bone metastases from prostate cancer result in poor patient prognosis^29^. Single-cell transcriptomics has been applied to solid tumors, involved bone marrow, and distal bone marrow from 9 prostate cancer patients with bone metastases, as well as benign bone marrow from 7 patients without cancer to study changes in gene expression and cell composition^30^. We applied *crumblr* to identify compositional differences associated with disease status. Variance partitioning analysis shows substantial variation in cell composition across patients, disease status of each biospecimen (categories: tumor, involved bone marrow, distal bone marrow, benign), and status of the patient (categories: cancer, non-cancer) (Fig. 5A). Univariate analysis of composition differences between solid tumor and involved bone marrow identified significant changes in 10 of the 28 cell types (Fig. 5B). A subset of monocytes showed the strongest decrease in frequency, while pericytes, osteoblasts and endothelial cells showed the strongest increase. Hierarchical clustering of gene expression profiles and multivariate testing identified the parent node of the three monocyte subsets as having significantly decreased frequency (*β* = −1.10, *P* = 9.28e-4, FDR = 3.93e-3), and the parent node of pericytes, osteoblasts and endothelial cells having significantly increased frequency (*β* = 2.74, *P* = 9.15e-0, FDR = 3.92e-3) (Fig. 5C). Examining pericytes across the four disease states shows a substantial increase in tumor samples (Fig. 5D). Pericytes reside on the outside of capillaries, regulate blood flow, and play a key role in tumor vascularization and microenvironment, so an increase in frequency is expected in tumor compared to involved bone marrow^30,31^.

### Compositional changes associated with infection response

**Fig. 6:**
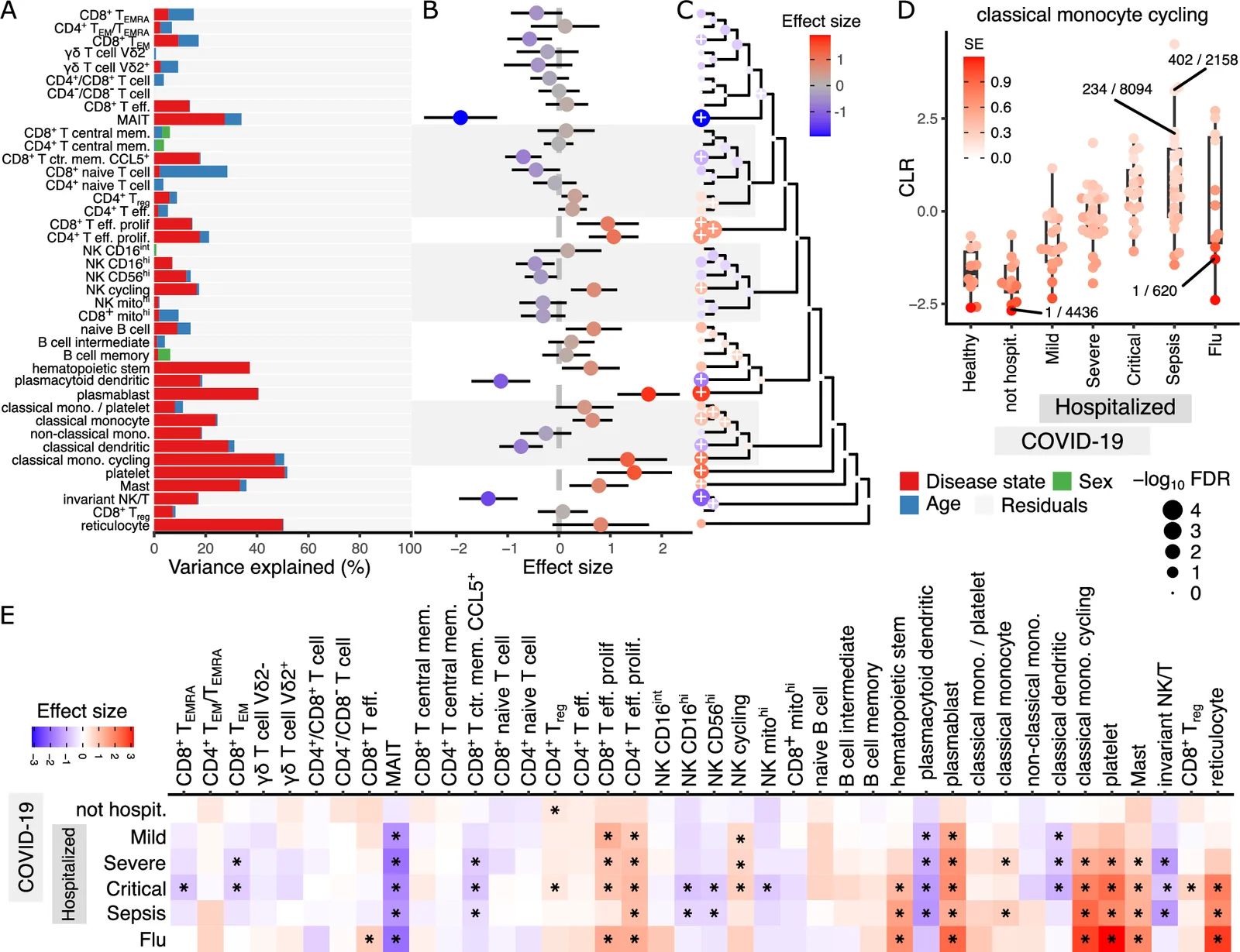
*crumblr* identifies compositional changes associated with infection. **A** Variance partitioning analysis quantifies the contribution of each variable to variation in frequency for each cell type. **B** Estimated effect size corresponding to leaves in (**A**) comparing composition between patients with severe COVID and healthy controls. A positive coefficient estimate indicates an increase in cell type frequency with infection. The color indicates effect size, and error bars indicate a 95% confidence interval. **C** Hierarchical clustering of cell types with colored points indicating estimated effect sizes for each cell type at leaves and multivariate testing at internal nodes. Results on leaves match results in (**B**). Point size indicates FDR, and “+” indicates FDR < 5%. **D** Transformed frequency of cycling classical monocytes for each disease state. Each point indicates a single donor, and the color indicates the standard error from limited measurement precision using *crumblr*. A subset of points is labeled with the observed cell type fraction with, for example, 1/620 indicating 1 cycling classical monocyte out of 620 total cells. Boxes indicate mean, first and third quartiles, whiskers indicate 1.5 interquartile range. **E** Effect size estimates comparing cell type frequency to healthy controls. Color indicates effect size, and ‘*’ indicates FDR < 5%.

Viral and bacterial infections trigger an immune response, causing changes in gene expression and cell composition in the blood. In order to study immune response to SARS-CoV-2 infection, the COMBAT Consortium^32^ collected blood from patients hospitalized with COVID-19, along with blood from non-hospitalized COVID-19 patients, healthy controls, and patients with sepsis or flu. They compiled a single-cell transcriptome resource of 787 K cells from 121 donors and identified 40 data-derived cell clusters. We applied *crumblr* to identify compositional differences associated with disease status. Variance partitioning analysis identified disease status as a major source of compositional variation, explaining more than 10% variance in 18 cell clusters (Fig. 6A). Notably, age explains 26.4% of the variance in CD8^+^ naive T cells, and this cluster decreases in frequency with age (*β* = −0.271, *P* = 6.2e-10, FDR = 2.5e-8). Univariate analysis of the compositional difference between patients with severe COVID and healthy controls identifies significant decreases in six cell clusters, with the largest decreases in MAIT, plasmacytoid dendritic cell, and invariant natural killer T cells (Fig. 6B). Meanwhile, eight cell clusters increased in frequency, with the largest seen in plasmablasts, platelets, and cycling classical monocytes. Hierarchical clustering using gene expression profiles and multivariate testing shows limited higher-order changes in cell type frequency (Fig. 6C). Examining classical cycling monocytes shows a baseline frequency shared between health donors and COVID-19 patients who have not been hospitalized, while frequency increases with COVID-19 severity in hospitalized patients to a level equal to that in sepsis patients (Fig. 6D). This increase in frequency with COVID-19 severity is observed across multiple cell types (Fig. 6E).

## Discussion

Here, we present the *crumblr* statistical framework for differential analysis of cellular composition to identify cell types that change frequency with a variable of interest. Across a broad range of simulation conditions, *crumblr* achieves high power while controlling the false positive rate. In addition to univariate tests of differential frequency, *crumblr* performs statistical testing at multiple levels of the cell lineage hierarchy using multivariate regression to increase power over tests of one cell component. The *crumblr* framework is fast, scalable to large single-cell datasets, and integrates with existing R/Bioconductor workflows from dreamlet^33^, variancePartition^17,34^, and limma^35^.

Applying *crumblr* to 4 published single-cell datasets identified biologically important changes in cellular composition. Analysis of T cells after tuberculosis infection identified multiple clusters of CD4^+^ T_h_1 and T_h_17 cells that decreased in frequency. Analysis of bone metastases from prostate cancer identified a decrease in monocyte frequency, and analysis of SARS-CoV-2 infection response identified an increase in cycling classical monocytes that tracked with COVID-19 severity. Finally, analysis of compositional changes in blood with aging in the OneK1K cohort identified a decrease in naive CD8^+^ αβ T cells and mucosal invariant T cells with age. This trend between frequency and age is also observed in the COMBAT cohort, and is consistent with recent work on dynamics of T-cell frequency with age^7,36^. Changes in the thymus over the human lifespan are known to result in decreased production of naive T cells with age^24^.

Here, we demonstrated the importance of modeling variation in measurement precision in count ratios when analyzing changes in cell frequency. Based on statistical theory, simulations, and observations from real data, we see that measurement precision increases with cell frequency and with the total number of cells observed. Modeling this variable precision is especially important when the total number of counts of the cell frequency varies widely across samples.

The open source *crumblr* package and documentation are available at DiseaseNeurogenomics.github.io/crumblr and Bioconductor and will enable powerful analysis of differences in cellular composition.

## Methods

This work complies with all relevant ethical regulations in accordance with the institutional review board (IRB) of the Icahn School of Medicine at Mount Sinai. No new data was generated as part of this work.

### Centered log-ratio transform for compositional data

Let the vector p store the proportions for each of D cell types. Then, the CLR transform of the proportion for cell type i is

1 $${{\mbox{clr}}}({p}_{i})=\log ({p}_{i})-\frac{1}{D}{\sum }_{j=1}^{D}\log ({p}_{j})$$

The CLR transform is widely used for compositional data analysis because it satisfies scale invariance, invariance to selection of the category used as a reference, and produces unbounded values that can be well approximated by a normal distribution^12,15^. Importantly, the CLR transform is a linear combination (i.e., weighted sum) of the log proportions. This property is central to the convenient form of the results described below.

Consider the observed proportions as random variables sampled from a Dirichlet-multinomial distribution with overdispersion parameter τ. As the number of counts, n, increases, the distribution of the estimated proportion converges to

2 $$\hat{{{{\bf{p}}}}}{\to }^{d}{{{\mathscr{N}}}}\left({{{\bf{p}}}},\frac{\tau }{n}\left[{{{\rm{diag}}}}({{{\bf{p}}}})-{{{{\bf{pp}}}}}^{T}\right]\right)$$

Setting $\tau=\,1$ corresponds to the multinomial distribution, and τ>1 indicating overdispersion corresponds to the more general Dirichlet-multinomial distribution.

Using the delta method to obtain an asymptotic normal approximation of the CLR-transformed proportion under the Dirichlet-multinomial model (Supplementary Methods), we can approximate the sampling variance of the CLR-transformed sample proportions:

3 $$\widehat{{{{\rm{var}}}}[{{{\rm{clr}}}}({\hat{p}}_{i})]}=\frac{\hat{\tau }}{n}\left[\frac{1}{{\hat{p}}_{i}}-\frac{2}{D{\hat{p}}_{i}}+\frac{1}{{D}^{2}}{\sum }_{j=1}^{D}\frac{1}{{\hat{p}}_{j}}\right]$$

Tests of the association are performed by fitting a linear or linear mixed model for each cell type using CLR-transformed proportions as the response and the inverse variances as precision weights. Importantly, the estimated coefficient values and their covariance are invariant to the scaling of the precision weights. Since the overdispersion is multiplicative and the results are scaling invariant, the value of τ can be set to 1 instead of being estimated from the data. This property enables our framework to be widely applicable to cases of overdispersion, including cases where the overdispersion factor varies across components.

For real data, the asymptotic variance formula can give weights that vary substantially across samples and give very high weights for a subset of samples. In order to address this, we regularize the weights to reduce the variation in the weights to have a maximum ratio (default of 5) between the maximum and specified quantile value (default of 5%). Performance is robust to changes in this parameter across a wide range of values, and default max.ratio=5 retains high power and controls the false positive rate in simulations (Supplementary Fig. 12). In addition, a pseudocount value of 0.5 is added to all counts, allowing *crumblr* to handle zero counts. Performance is robust to changes in this parameter across a wide range of values, and the default value retains high power and controls the false positive rate in simulations (Supplementary Fig. 13). We note that the defaults for both of these values were set during the software’s development, not as a result of these simulations.

Regression models are fit separately across each of the D cell types. An empirical Bayes moderated t-statistic^37^ is used by applying an inverse gamma prior to the residual variance for each cell type component. For linear mixed models, a recent extension is used to estimate the residual degrees of freedom for the empirical Bayes step^17,33^.

### Multivariate testing combining multiple cell-type components

Consider a multivariate regression with n samples, c variables, and m response values using design matrix ${X}_{n\times c}$ and responses stored as columns in the matrix ${Y}_{n\times m}$. Standard results from multivariate regression with no weights (i.e., all samples have equal weights) give coefficient estimates

4 $$\hat{B}={({X}^{T}X)}^{-1}{X}^{T}Y,$$

and variance–covariance matrix

5 $${{{\mathrm{var}}}}({{\mbox{vec}}}(\hat{B}))={{{\mathrm{cov}}}}(R)\otimes {({X}^{T}X)}^{-1}$$

where $R=Y-X\hat{B}$ is the matrix of residuals, the vec (.) operator converts a matrix to a column vector, and ⊗ is the Kronecker product.

These coefficients and the variance–covariance matrix can then be used in hypothesis testing across responses in the multivariate model. Here, we use a fixed effects meta-analysis to account for covariance between the coefficient estimates^20^. Letting ${\hat{B}}_{k}$ be the vector of coefficient estimates for variable k across m responses, $\hat{\varOmega }$ be the estimated covariance matrix of the coefficients from the multivariate regression, and **1** be a vector of 1’s, the test statistic

6 $$\frac{{{{{\bf{1}}}}}^{T}{\hat{\varOmega }}^{-1}{\hat{B}}_{k}}{{{{{\bf{1}}}}}^{T}{\hat{\varOmega }}^{-1}{{{\bf{1}}}}}$$

is asymptotically normally distributed under the null. We note that when the covariance $\hat{\varOmega }$ is diagonal, so there is no covariance between coefficient estimates; this reduces to standard fixed effects meta-analysis^38^.

### Multivariate hierarchical testing with treeTest()

In the field of single-cell transcriptomics, the number of cell types analyzed varies widely across studies. For example, Mathys et al.^39^ perform analysis on 12 major cell types and 54 higher-resolution clusters from human prefrontal cortex to identify gene expression changes in Alzheimer’s disease, while the BRAIN Initiative Cell Census Network identified 31 cell superclusters, 461 clusters, and 3313 subclusters across multiple brain regions^40^. In the brain, there is vast diversity in neuronal subtypes, but much less diversity in other cell types^40^. In practice, the number of cell type clusters used is determined by the tissue, the number of cells observed, and the biological question.

Yet in analyses of differential cell type composition, the number of cell clusters used can affect the analysis due to the tradeoff between resolution and statistical power. While increasing the number of cell clusters enhances cell type resolution and the biological specificity of each cluster, the frequency of each higher-resolution cluster decreases. All analysis methods we considered suffer from a loss in power as the cell-type frequency decreases. This fundamental tradeoff leaves the analyst to select the appropriate number of cell types to address their biological question.

Instead of selecting this number at the beginning of a project, the treeTest() analysis in the *crumblr* framework performs tests of differential abundance at multiple levels of cell type resolution. Given a hierarchical clustering of cell types, the method performs a multivariate hypothesis test on each internal node in the hierarchy. Given total cell clusters at the leaves of this hierarchy, consider an internal node ℓ with descendant leaves. Letting be the vector of estimated coefficients from descendant leaves of node ℓ, we want to test the null hypothesis that the mean effect size is zero. Since high-resolution cell clusters often have frequencies that covary, modeling covariance in the estimated coefficients increases power to reject the null hypothesis while controlling the false positive rate. This test of the mean effect of descendant leaf nodes is repeated for each internal node using the empirical Lin–Sullivan test.

By testing at internal nodes, this approach can accumulate power across multiple leaves where testing at a single leaf is underpowered. Rejecting the null hypothesis at an internal node where few or no leaves show a significant signal can indicate that high-resolution clustering caused loss of power at the leaves, which is then “rescued” by the multilevel analysis. However, note that a strong signal in one leaf can drive the result at an internal node where all other leaves have small effects, as we see in Fig. 3B. We also note that the results depend on the hierarchical clustering used.

### Extension to weighted multivariate regression

When weights are not equal to 1 and vary for each response, the coefficient estimates and their covariance have a more complicated form. Letting ${w}^{\left(i\right)}$ be the vector of weights for response i, ${W}^{\left(i\right)}={\mbox{diag}}\left({w}^{\left(i\right)}\right)$, and ${y}_{i}$ be the vector storing response i, the coefficient estimates for response i are

7 $${\hat{\beta }}_{i}={\left({\tilde{X}}_{i}^{T}{\tilde{X}}_{i}\right)}^{-1}\tilde{X}_{i}^{T}{\tilde{y}}_{i},$$

where ${\tilde{X}}_{i}$ and ${\tilde{y}}_{i}$ represent the weighted design and response according to ${\tilde{M}}_{i}=M{\mbox{diag}}\left(\sqrt{{w}^{\left(i\right)}}\right)$ for any matrix or vector M. The covariance between coefficient estimates for responses i and j is

8 $${{cov}}\left({\hat{\beta }}_{i},{\hat{\beta }}_{j}\right)={\tilde{C}}_{i,j}{\left({\tilde{X}}_{i}^{T}{\tilde{X}}_{i}\right)}^{-1}\left({\tilde{X}}_{i}^{T}{\tilde{X}}_{j}\right){\left({\tilde{X}}_{j}^{T}{\tilde{X}}_{j}\right)}^{-1}$$

where $\tilde{C}={\tilde{r}}_{i}^{T}\tilde{r}_{j}/\nu$, ${\tilde{r}}_{i}$ is the weight residuals for response i, and ν is the residual degrees of freedom. When all weights are 1, this reduces to the standard results above.

### Extension to weighted linear mixed models

For the linear mixed model, closed forms don’t exist. Instead, the coefficients and variance–covariance matrix are estimated using numerical optimization^41^. Letting ${\hat{\varSigma }}_{i}$ be the estimated variance–covariance matrix between coefficients for response i, the covariance between coefficients for responses i and j can be approximated as

9 $${{cov}}\left({\hat{\beta }}_{i},{\hat{\beta }}_{j}\right)={cor}({\tilde{r}}_{i},{\tilde{r}}_{j}){\hat{\varSigma }}_{i}\left({\hat{\varSigma }}_{i}^{-\frac{1}{2}}{\hat{\varSigma }}_{j}^{-\frac{1}{2}}\right){\hat{\varSigma }}_{j}.$$

When this formula is applied to the fixed effect model, it is equivalent to the previous formula when the weights vary across samples but are shared across responses so that ${w}^{\left(i\right)}$ and ${w}^{\left(j\right)}$ are equal (Supplementary Methods).

### Time complexity

Evaluating the CLR transform and the precision weights with *crumblr* is linear in the number of samples, n, and cell types, D. It is very fast and takes <1 s for 1000 samples and 100 cell types. Fixed effect linear regression with c covariates, is ${{{\mathscr{O}}}}({n}^{2}c+{c}^{3})$ and requires just a few seconds even for large datasets. Linear mixed models are linear in n, but the complexity as a function of c is more complicated because estimation uses sparse matrix encoding of random effects and uses iterative methods to maximize the log-likelihood^41^. Combining results across D cell types using the Lin-Sullivan statistic is ${{{\mathscr{O}}}}({D}^{3})$. Using the Monte Carlo sampling, combining effects along a tree of 30 leaves takes <30 s.

### Note on statistical modeling

The model we propose here considers the sampling variance of the CLR-transformed proportions, but does not consider the sampling *covariance* between cell types. The model performs weighted fixed or mixed-effects regression under a normal model, and analyses each response (i.e., cell type) separately. It is natural to consider the prospect of modeling this covariance in errors. In the case of a fixed effect model, this would involve modeling the data with a matrix-normal distribution. This type of model is called “seemingly unrelated regressions” or SUR to account for correlated errors^42^. SUR models should have increased statistical efficiency for correlated errors. Our current approach is a special case of the matrix-normal distribution, modeling responses as having independent errors.

This more sophisticated model is indeed an interesting research question, but its potential value and tractability require further thought. First, in empirical data, cell type proportions are indeed often correlated. Yet, this is likely due to the biology of these cell types rather than sampling covariance. More work to understand the importance of the error covariance term in realistic data is needed. Second, modeling the error covariance across D cell types would require inverting a D×D matrix and require ${{{\mathscr{O}}}}({D}^{3})$ time. Third, extending a SUR-type model to incorporate random effects is challenging. The *crumblr* model uses standard R packages to fit the regression models for each response, and it is unclear if this extension could be accommodated by existing software.

### Comparison with other methods

Statistical testing of differences in cell type frequency typically uses models based on frequencies, counts, normal approximations, or non-parametrics. Here, we summarize some of the assumptions and limitations of models in our comparison. Binomial models for frequency data can be fit with a generalized linear model (GLM), but do not model overdispersion or heteroskedasticity due to differences in total counts per sample. Beta-binomial models add an overdispersion term. Poisson models of counts can be fit with a GLM, but do not model overdispersion. Negative binomial models extend the Poisson model to accommodate overdispersion. DESeq2 and edgeR are negative binomial models designed for RNA-seq and use a Bayesian method to borrow information across features (i.e., either genes or cell clusters). However, this information borrowing was not designed for the relatively small number of cell clusters and can fail in the case of DESeq2 (see Supplementary Fig. 6). The multinomial and Dirichlet-multinomial distributions are often used to simulate count data. But performing parameter estimation and hypothesis testing for complex study designs with covariates is challenging.

For a normal approximation, the count data is transformed so that the sampling variance of each observation is approximately equal, and the new values can be fit with a linear model. Transformations include CLR, asin, log, logit, and identity (where regression is performed on the fractions directly).

The *crumblr* method uses a more sophisticated normal approximation using weighted regression based on the Dirichlet-multinomial distribution. This allows *crumblr* to retail high power and speed, while accommodating random effects and multivariate testing.

Tests of differential frequencies are also important in the microbiome field. For example, ANCOM-BC is designed for sparse microbiome datasets, models uncertainty in the observed counts, and uses a bias correction as an offset in the regression^43^. This bias correction accounts for variation in how well observed cell proportions from a small tissue sample are representative of the true cell proportion of the entire tissue. ALDEx2 models uncertainty in the observed counts by using a “multiple imputation” approach^44^. A Dirichlet model is first used to fit the observed data. Multiple sample datasets are drawn from this distribution, the CLR transform is applied, and a regression analysis is performed on each one. Summary statistics are then computed across the Monte Carlo samples.

Hypothesis testing for differential cell composition was performed with a range of existing methods that either modeled the cell fraction or directly modeled the number of observed counts (Supplementary Methods).

### Simulations of multivariate testing

In the first simulation, *m* = 15 responses were used and sample size ranges from 50 to 500 with increments of 50. In the second simulation, 200 samples were simulated with m in 2 to 22 responses in increments of 4. In both cases, 100k simulations were performed using a design matrix including an intercept term and a variable sampled for a standard normal, an effect size of 0.25 shared across response, error covariance sampled for a multivariate normal using clusterGeneration::genPositiveDefMat(m, ratioLambda = 100).

### Analysis of compositional data from single-cell datasets

Single-cell transcriptomics data were obtained for peripheral blood mononuclear cells^22^, T cells following tuberculosis infection^26^, bone metastases from prostate cancer^30^, and blood following SARS-CoV-2 infection^32^. For all datasets, no additional processing was performed, and cell type annotations provided by the original studies were used in our analysis.

### Software versions


R v.4.4.1crumblr v0.99.11remaCor 0.0.18variancePartition v1.36.3lme4 1.1.35.5scCODA v0.1.9.


### Reporting summary

Further information on research design is available in the Nature Portfolio Reporting Summary linked to this article.

## Supplementary information

Supplementary Information

Reporting Summary

Transparent Peer Review file

## Data Availability

The *crumblr* R package and documentation are available at DiseaseNeuroGenomics.github.io/crumblr and bioconductor.org/packages/crumblr. The multivariate regression analysis proposed here is implemented in our variancePartition package using the function mvTest() to post-process results from dream(). Multivariate hypothesis testing is implemented in our remaCor package, which includes the extension of the Lin-Sullivan test for small sample sizes. Code for analysis and simulations is available at https://github.com/GabrielHoffman/crumblr_analysis. Code and analysis are also available from Zenodo (https://doi.org/10.5281/zenodo.18176987, https://doi.org/10.5281/zenodo.18176993).

## References

[1] Zeng, H. What is a cell type and how to define it? *Cell* **185**, 2739–2755 (2022). 10.1016/j.cell.2022.06.031 PMC9342916 35868277

[2] Rood, J. E., Maartens, A., Hupalowska, A., Teichmann, S. A. & Regev, A. Impact of the Human Cell Atlas on medicine. *Nat. Med.* **28**, 2486–2496 (2022). 10.1038/s41591-022-02104-7 36482102

[3] Emani, P. S. et al. Single-cell genomics and regulatory networks for 388 human brains. *Science* **384**, eadi5199 (2024). 10.1126/science.adi5199 PMC11365579 38781369

[4] Rood, J. E. et al. The Human Cell Atlas from a cell census to a unified foundation model. *Nature* **637**, 1065–1071 (2025). 10.1038/s41586-024-08338-4 39566552

[5] Kumasaka, N. et al. Mapping interindividual dynamics of innate immune response at single-cell resolution. *Nat. Genet.* **55**, 1066–1075 (2023). 10.1038/s41588-023-01421-y PMC10260404 37308670

[6] Braun, E. et al. Comprehensive cell atlas of the first-trimester developing human brain. *Science* **382**, eadf1226 (2023). 10.1126/science.adf1226 37824650

[7] Terekhova, M. et al. Single-cell atlas of healthy human blood unveils age-related loss of NKG2C + GZMB-CD8+ memory T cells and accumulation of type 2 memory T cells. *Immunity* **56**, 2836–2854.e9 (2023). 10.1016/j.immuni.2023.10.013 37963457

[8] Lee, D. et al. Single-cell atlas of transcriptomic vulnerability across multiple neurodegenerative and neuropsychiatric diseases. *Nature* 10.1101/2024.10.31.24316513 (2025).

[9] Yang, H. et al. A single-cell transcriptomic atlas of the prefrontal cortex across the human lifespan. *Nature* 10.1101/2024.11.06.24316592 (2025). PMC13600939 42778698

[10] Law, C. W., Chen, Y., Shi, W. & Smyth, G. K. Voom: precision weights unlock linear model analysis tools for RNA-seq read counts. *Genome Biol.* **15**, R29 (2014). 10.1186/gb-2014-15-2-r29 PMC4053721 24485249

[11] Love, M. I., Huber, W. & Anders, S. Moderated estimation of fold change and dispersion for RNA-seq data with DESeq2. *Genome Biol*. **15**, 550 (2014). 10.1186/s13059-014-0550-8 PMC4302049 25516281

[12] Aitchson, J. *The Statistical Analysis of Compositional Data* (Chapman and Hall, 1986).

[13] Phipson, B. et al. Propeller: Testing for differences in cell type proportions in single cell data. *Bioinformatics* **38**, 4720–4726 (2022). 10.1093/bioinformatics/btac582 PMC9563678 36005887

[14] Büttner, M., Ostner, J., Müller, C. L., Theis, F. J. & Schubert, B. scCODA is a Bayesian model for compositional single-cell data analysis. *Nat. Commun.* **12**, 6876 (2021). 10.1038/s41467-021-27150-6 PMC8616929 34824236

[15] van den Boogaart, K. G. & Tolosana-Delgado, R. *Analyzing Compositional Data with R* (Springer Berlin Heidelberg, 2013).

[16] Greenacre, M. Compositional data analysis. *Annu. Rev. Stat. Appl.* **8**, 271–299 (2021).

[17] Hoffman, G. E. & Roussos, P. Dream: powerful differential expression analysis for repeated measures designs. *Bioinformatics* **37**, 192–201 (2021). 10.1093/bioinformatics/btaa687 PMC8055218 32730587

[18] Liu, Y. et al. ACAT: a fast and powerful p value combination method for rare-variant analysis in sequencing studies. *Am. J. Hum. Genet.* **104**, 410–421 (2019). 10.1016/j.ajhg.2019.01.002 PMC6407498 30849328

[19] Wilson, D. J. The harmonic mean p-value for combining dependent tests. *Proc. Natl. Acad. Sci. USA* **116**, 1195–1200 (2019). 10.1073/pnas.1814092116 PMC6347718 30610179

[20] Lin, D.-Y. & Sullivan, P. F. Meta-analysis of genome-wide association studies with overlapping subjects. *Am. J. Hum. Genet.* **85**, 862–872 (2009). 10.1016/j.ajhg.2009.11.001 PMC2790578 20004761

[21] Mogilenko, D. A., Shchukina, I. & Artyomov, M. N. Immune ageing at single-cell resolution. *Nat. Rev. Immunol.* **22**, 484–498 (2022). 10.1038/s41577-021-00646-4 PMC8609266 34815556

[22] Yazar, S. et al. Single-cell eQTL mapping identifies cell type-specific genetic control of autoimmune disease. *Science* **376**, eabf3041 (2022). 10.1126/science.abf3041 35389779

[23] Zhang, H., Weyand, C. M. & Goronzy, J. J. Hallmarks of the aging T-cell system. *FEBS J.* **288**, 7123–7142 (2021). 10.1111/febs.15770 PMC8364928 33590946

[24] Mittelbrunn, M. & Kroemer, G. Hallmarks of T cell aging. *Nat. Immunol.* **22**, 687–698 (2021). 10.1038/s41590-021-00927-z 33986548

[25] Sun, L., Su, Y., Jiao, A., Wang, X. & Zhang, B. T cells in health and disease. *Signal Transduct. Target. Ther.* **8**, 235 (2023). 10.1038/s41392-023-01471-y PMC10277291 37332039

[26] Nathan, A. et al. Multimodally profiling memory T cells from a tuberculosis cohort identifies cell state associations with demographics, environment and disease. *Nat. Immunol.* **22**, 781–793 (2021). 10.1038/s41590-021-00933-1 PMC8162307 34031617

[27] Sun, M. et al. Specific CD4 + T cell phenotypes associate with bacterial control in people who ‘resist’ infection with Mycobacterium tuberculosis. *Nat. Immunol.* **25**, 1411–1421 (2024). 10.1038/s41590-024-01897-8 PMC11291275 38997431

[28] Flores-Gonzalez, J. et al. The presence of cytotoxic CD4 and exhausted-like CD8 + T-cells is a signature of active tuberculosis. *Biochim. Biophys. Acta Mol. Basis Dis.* **1870**, 167219 (2024). 10.1016/j.bbadis.2024.167219 38734321

[29] Halabi, S. et al. Meta-analysis evaluating the impact of site of metastasis on overall survival in men with castration-resistant prostate cancer. *J. Clin. Oncol.* **34**, 1652–1659 (2016). 10.1200/JCO.2015.65.7270 PMC4872320 26951312

[30] Kfoury, Y. et al. Human prostate cancer bone metastases have an actionable immunosuppressive microenvironment. *Cancer Cell* **39**, 1464–1478.e8 (2021). 10.1016/j.ccell.2021.09.005 PMC8578470 34719426

[31] Jiang, Z. et al. Pericytes in the tumor microenvironment. *Cancer Lett.* **556**, 216074 (2023). 10.1016/j.canlet.2023.216074 36682706

[32] COMBAT Consortium A blood atlas of COVID−19 defines hallmarks of disease severity and specificity. *Cell* **185**, 916–938.e58 (2022). 10.1016/j.cell.2022.01.012 PMC8776501 35216673

[33] Hoffman, G. E. et al. Efficient differential expression analysis of large-scale single cell transcriptomics data using Dreamlet. *Nat. Commun.*10.1038/s41467-026-75680-8 (2025).42778542

[34] Hoffman, G. E. & Schadt, E. E. variancePartition: interpreting drivers of variation in complex gene expression studies. *BMC Bioinformatics* **17**, 483 (2016). 10.1186/s12859-016-1323-z PMC5123296 27884101

[35] Ritchie, M. E. et al. limma powers differential expression analyses for RNA-sequencing and microarray studies. *Nucleic Acids Res.* **43**, e47 (2015). 10.1093/nar/gkv007 PMC4402510 25605792

[36] Filippov, I., Schauser, L. & Peterson, P. An integrated single-cell atlas of blood immune cells in aging. *NPJ Aging* **10**, 59 (2024). 10.1038/s41514-024-00185-x PMC11606963 39613786

[37] Smyth, G. K. Linear models and empirical bayes methods for assessing differential expression in microarray experiments. *Stat. Appl. Genet. Mol. Biol.* **3**, 1544–6115 (2004). 10.2202/1544-6115.1027 16646809

[38] Han, B. et al. A general framework for meta-analyzing dependent studies with overlapping subjects in association mapping. *Hum. Mol. Genet.* **25**, 1857–1866 (2016). 10.1093/hmg/ddw049 PMC4986332 26908615

[39] Mathys, H. et al. Single-cell multiregion dissection of Alzheimer’s disease. *Nature* **632**, 858–868 (2024). 10.1038/s41586-024-07606-7 PMC11338834 39048816

[40] Siletti, K. et al. Transcriptomic diversity of cell types across the adult human brain. *Science* **382**, eadd7046 (2023). 10.1126/science.add7046 37824663

[41] Bates, D., Mächler, M., Bolker, B. & Walker, S. Fitting linear mixed-effects models using lme4. *J. Stat. Softw*. **67**, 1–48 (2015).

[42] Zellner, A. An efficient method of estimating seemingly unrelated regressions and tests for aggregation bias. *J. Am. Stat. Assoc.* **57**, 348–368 (1962).

[43] Lin, H. & Peddada, S. D. Analysis of compositions of microbiomes with bias correction. *Nat. Commun.* **11**, 3514 (2020). 10.1038/s41467-020-17041-7 PMC7360769 32665548

[44] Fernandes, A. D., Macklaim, J. M., Linn, T. G., Reid, G. & Gloor, G. B. ANOVA-like differential expression (ALDEx) analysis for mixed population RNA-Seq. *PLoS ONE* **8**, e67019 (2013). 10.1371/journal.pone.0067019 PMC3699591 23843979

