# Supplementary material for: Fast, flexible analysis of differences in cellular composition with crumblr
Source: Nat Commun. 2026 Sep 23;17:9598. doi: 10.1038/s41467-026-75681-7 (PMC13601534; doi:10.1038/s41467-026-75681-7)
Supplement: Supplementary file 1 [file 41467_2026_75681_MOESM1_ESM.pdf]

Supplementary Information:
Fast, flexible analysis of differences in cellular composition with `crumblr`

Gabriel E. Hoffman[1,2,3,4,5,6]* and Panos Roussos[1,2,3,4,5,6]*

[1] Center for Disease Neurogenomics,
[2] Department of Psychiatry,
[3] Department of Genetics and Genomic Sciences,
[4] Friedman Brain Institute,
Icahn School of Medicine at Mount Sinai, New York, NY, USA
[5] Center for Precision Medicine and Translational Therapeutics,
[6] Mental Illness Research, Education and Clinical Centers,
James J. Peters VA Medical Center, Bronx, New York, USA

*Correspondence to: G.E.H (gabriel.hoffman@mssm.edu) and P.R. (panagiotis.roussos@mssm.edu)

Contents

1 Count ratio uncertainty modeling with linear regression **12**
- 1.1 Isometric log ratio . . . 13
- 1.2 Modeling sampling variance in clr coordinates . . . 13
  - 1.2.1 Asymptotic normal approximation of multinomial counts . . . 14
  - 1.2.2 Asymptotic normal approximation to a Dirichlet-multinomial . . . 16
- 1.3 Multivariate modeling of cell type components . . . 17
  - 1.3.1 Weighted linear model . . . 18
  - 1.3.2 Weighted linear mixed model . . . 19
  - 1.3.3 Multivariate hypothesis testing . . . 20
  - 1.3.4 An adaptation for unknown variance . . . 20

2 Simulations **23**
- 2.1 Univariate hypothesis testing . . . 23
- 2.2 Multivariate hypothesis testing . . . 23

3 Implementation of other methods for comparison **23**

4 Additional proofs **24**

Supplementary Figures

Method	High power for univariate test	Controls false positive rate	Models varying measurement precision	Models random effects	Robust to overdispersion	Variance partitioning analysis	Multivariate testing	Fast (runtime < 10s)
`crumblr`	✓	✓	✓	✓	✓	✓	✓	✓
CLR		✓		✓		✓	✓	✓
Linear Model (frac)		✓		✓		✓	✓	✓
Linear Model (log)		✓		✓		✓	✓	✓
Linear Model (logit)		✓		✓		✓	✓	✓
Linear Model (asin)		✓		✓		✓	✓	✓
Binomial	✓							✓
Beta-binomial					✓			
NB	✓	✓	✓	✓	✓			✓
Poisson	✓		✓	✓				✓
DESeq2	✓	✓	✓		✓			✓
edgeR	✓	✓	✓		✓			✓
`scCODA`			✓		✓			

Supplementary Figure 1: Comparison of properties of each method. We note that incorporation of random effects, variance partitioning analysis and multivariate testing applies to all methods using a linear model, since these approaches are quite general. They could in principle be applied to generalized linear models as well, but this would require substantial methodological development. For 'Linear model', 'frac' indicates using the cell fraction, f, as the response, 'log' indicates $log(f)$, 'logit' indicates $logit(f) = log(f) - log(1-f)$, and 'asin' indicates $arcsin(\sqrt{f})$.

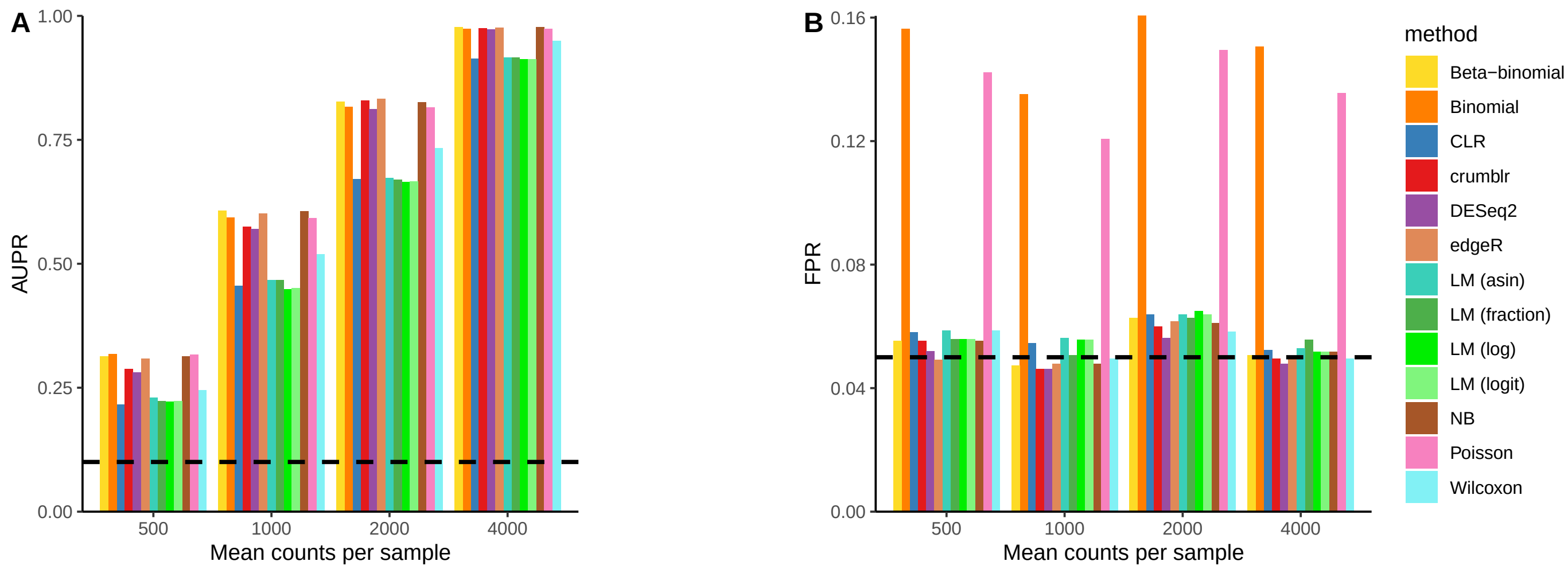

Supplementary Figure 2: Comparing performance for increasing cell counts A) Area under the precision recall curve. Dashed line indicates the performance of a random method. **B)** False positive rate computed as the fraction of p-values less than the target of 0.05 under a null model. Dashed line indicates the target value. Simulations were performed with 200 samples, 15 cell clusters, effect size of 0.05, mean of 2000 cells per sample, batch effect of zero, overdispersion $\tau = 10$, and 10K simulated datasets.

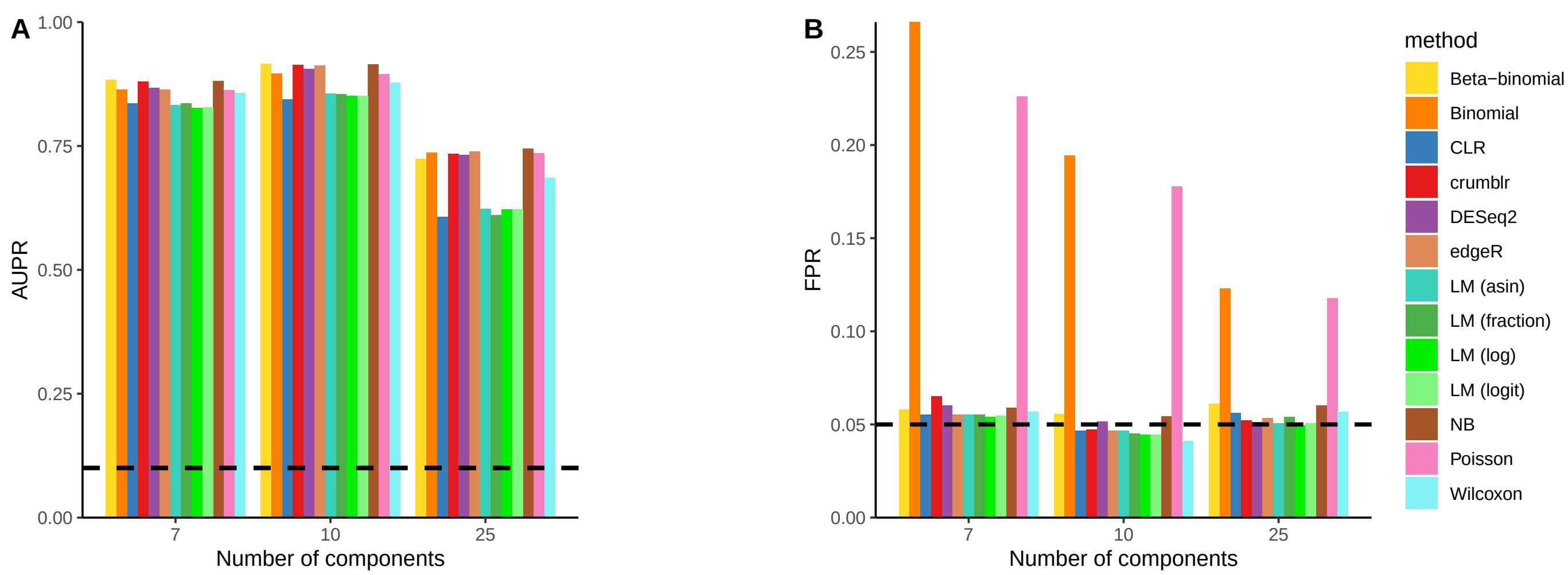

Supplementary Figure 3: Comparing performance for increasing cell cluster components. A) Area under the precision recall curve. Dashed line indicates the performance of a random method. **B)** False positive rate computed as the fraction of p-values less than the target of 0.05 under a null model. Dashed line indicates the target value. Simulations were performed with 200 samples, effect size of 0.05, mean of 2000 cells per sample, batch effect of zero, overdispersion $\tau = 10$, and 10K simulated datasets.

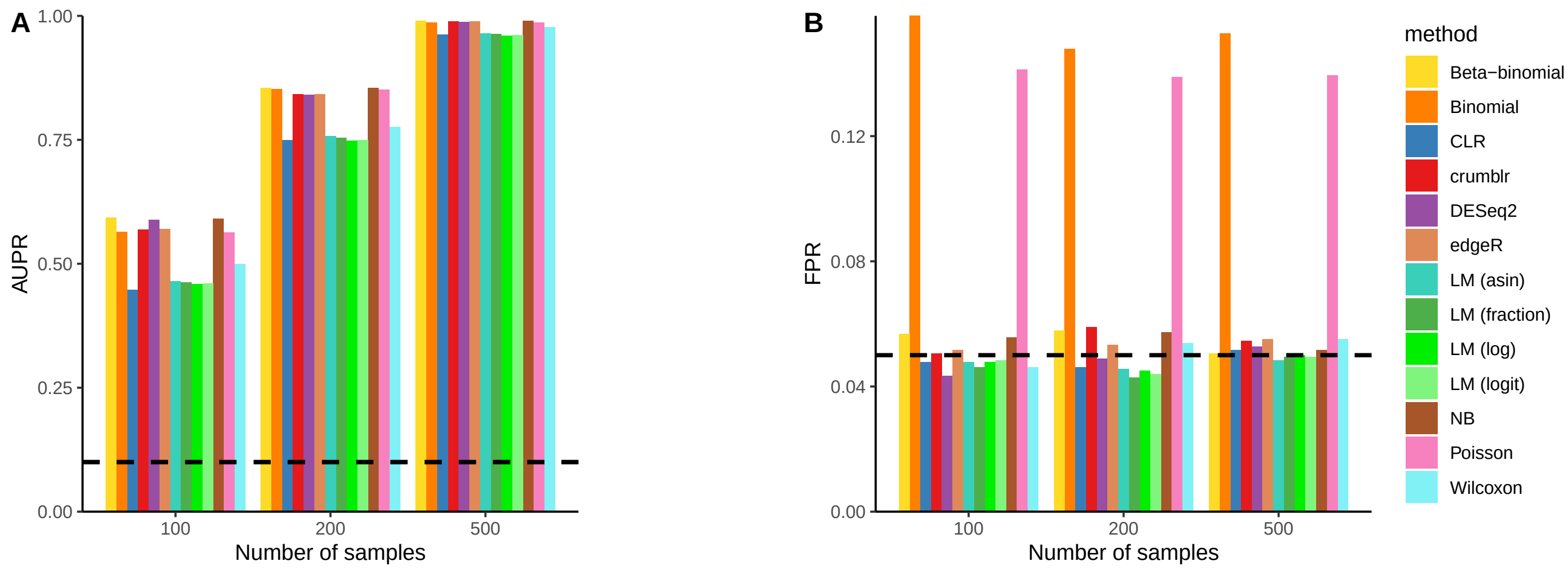

Supplementary Figure 4: Comparing performance for increasing sample size. A) Area under the precision recall curve. Dashed line indicates the performance of a random method. **B)** False positive rate computed as the fraction of p-values less than the target of 0.05 under a null model. Dashed line indicates the target value. Simulations were performed with 15 cell clusters, effect size of 0.05, mean of 2000 cells per sample, batch effect of zero, overdispersion $\tau = 10$, and 10K simulated datasets.

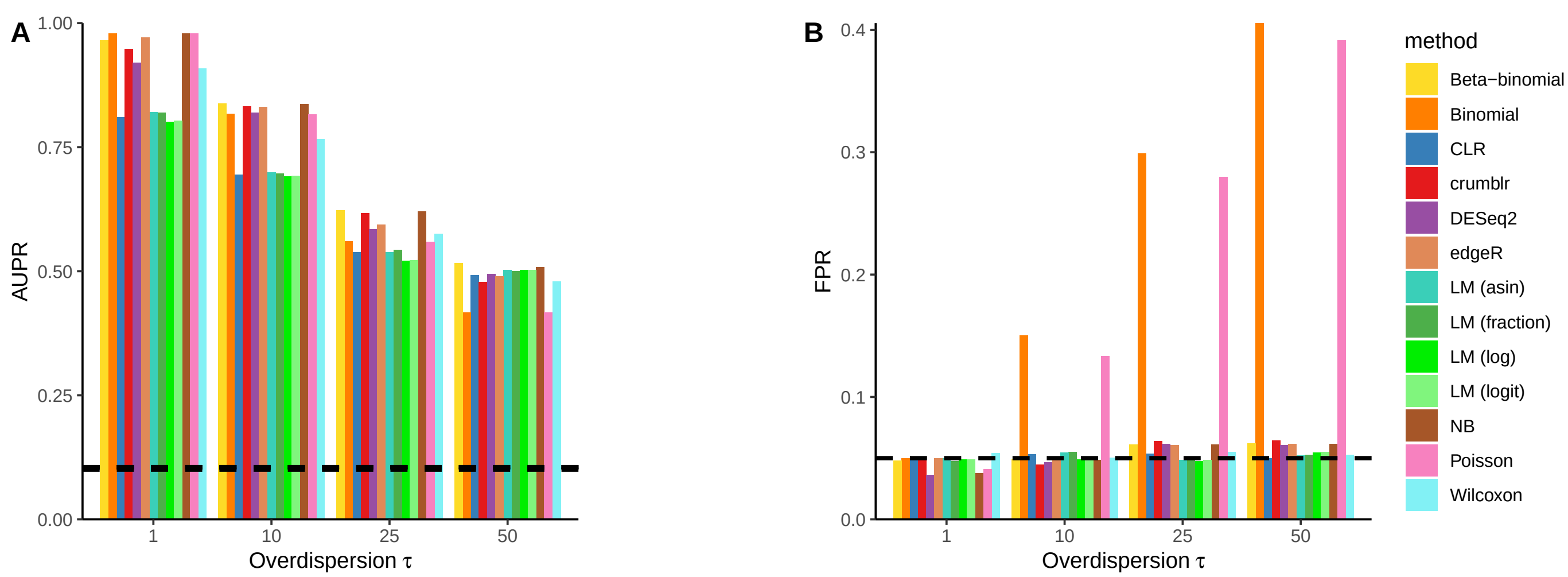

Supplementary Figure 5: Comparing performance for increasing overdispersion. A) Area under the precision recall curve. Dashed line indicates the performance of a random method. **B)** False positive rate computed as the fraction of p-values less than the target of 0.05 under a null model. Dashed line indicates the target value. Simulations were performed with 200 samples, 15 cell clusters, effect size of 0.05, mean of 2000 cells per sample, batch effect of zero, and 10K simulated datasets.

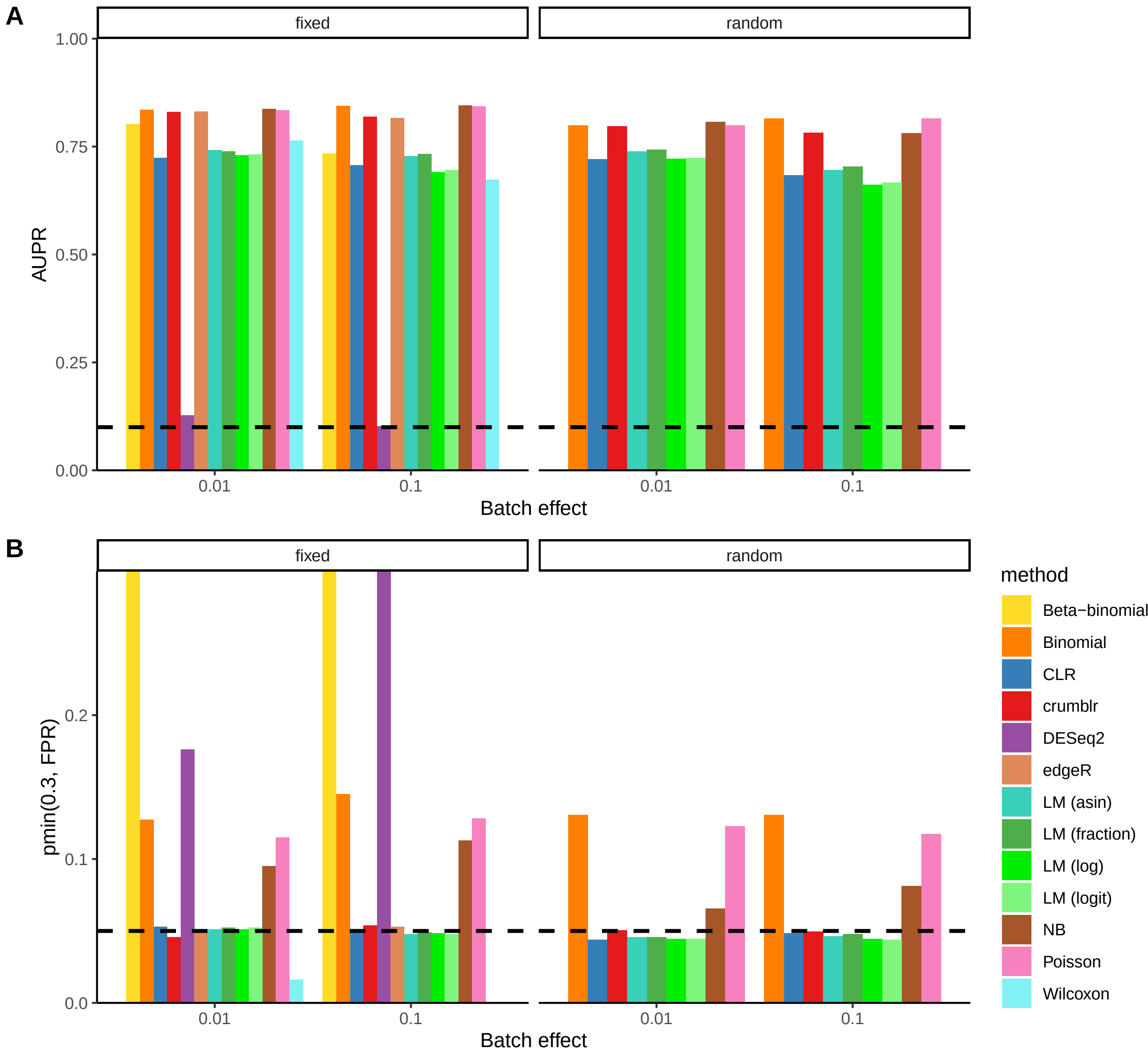

Supplementary Figure 6: Comparing performance for increasing batch effect Results are shown when accounting for batch as a fixed effect (left) or a random effect (right). **A)** Area under the precision recall curve. Dashed line indicates the performance of a random method. **B)** False positive rate computed as the fraction of p-values less than the target of 0.05 under a null model. Dashed line indicates the target value. Simulations were performed with 200 samples, 15 cell clusters, effect size of 0.05, mean of 2000 cells per sample, overdispersion $\tau = 10$, 50 batches, and 10K simulated datasets.

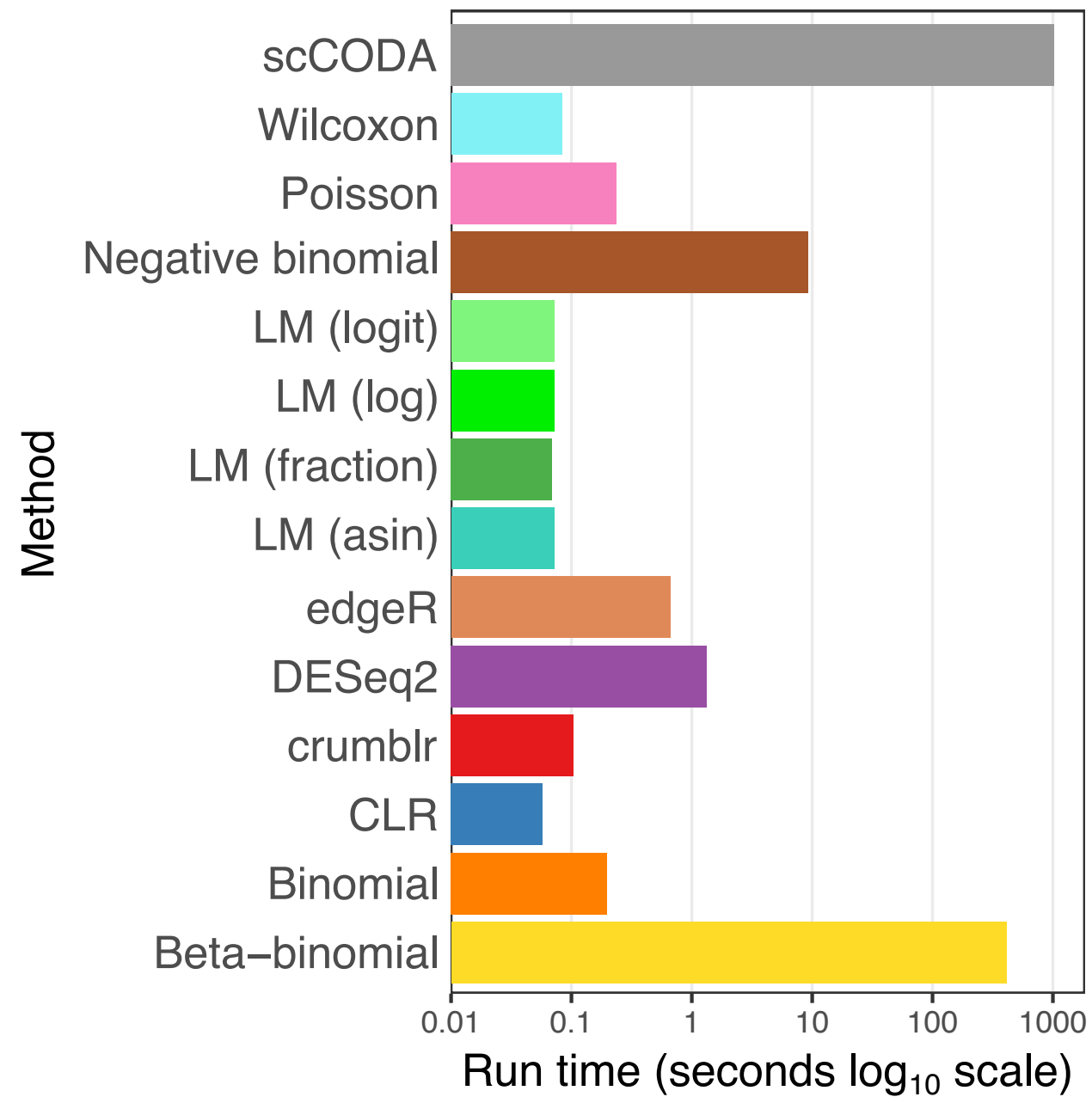

Supplementary Figure 7: Comparing compute times across methods. Run times for fixed effect models were computed using a dataset of 500 samples, 20 clusters, 3000 cell counts per dataset, and overdispersion $\tau = 10$.

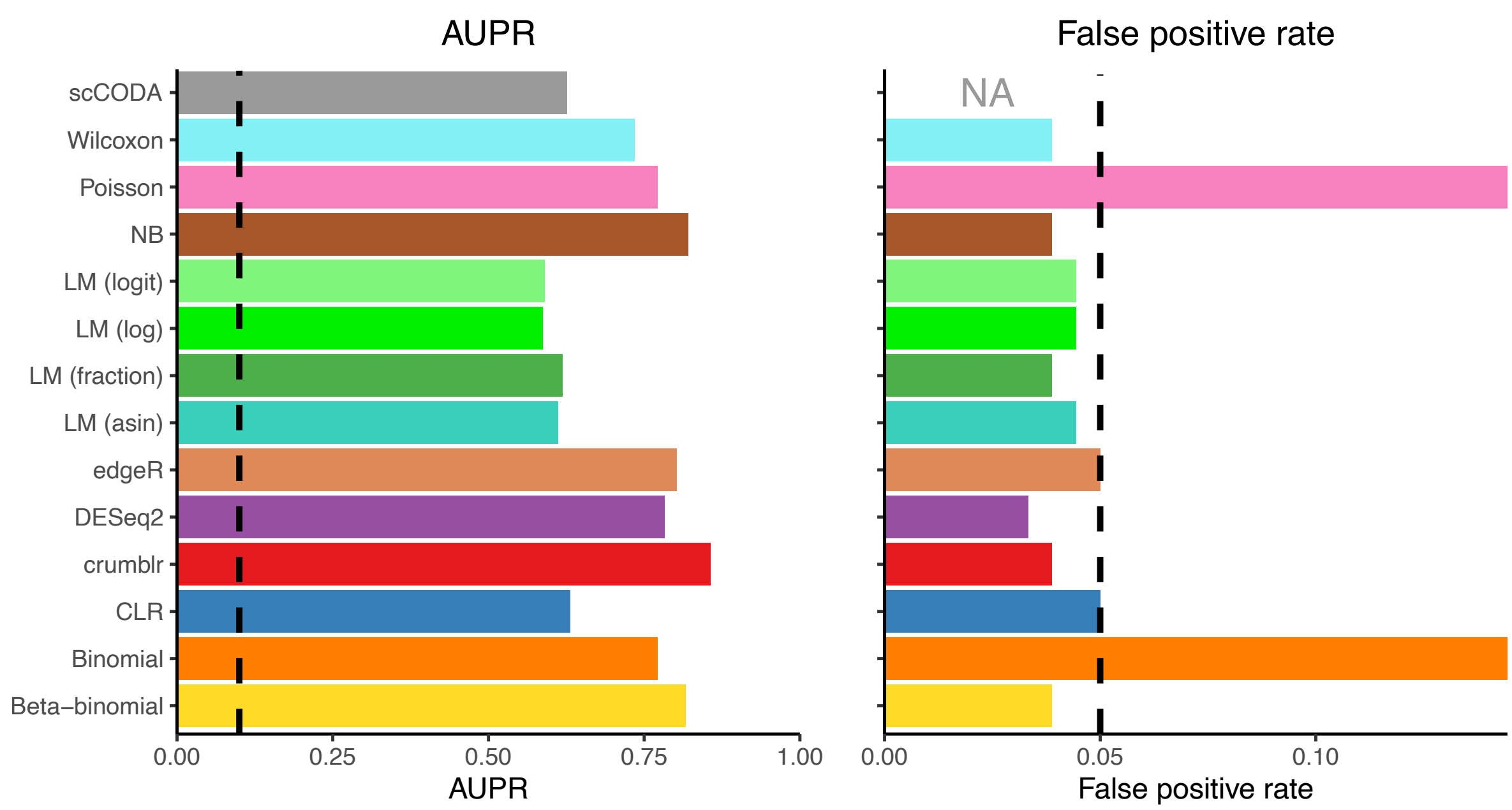

Supplementary Figure 8: Simulations using parameters identical to Figure 1A except using only 100 simulated datasets. scCODA is not competitive with the top methods based on area under the precision recall curve (left). Because scCODA is a Bayesian model, it does not report p-values and therefore the false positive rate under the null is not applicable here. The long run time prevented a more thorough investigation of the behavior of this model.

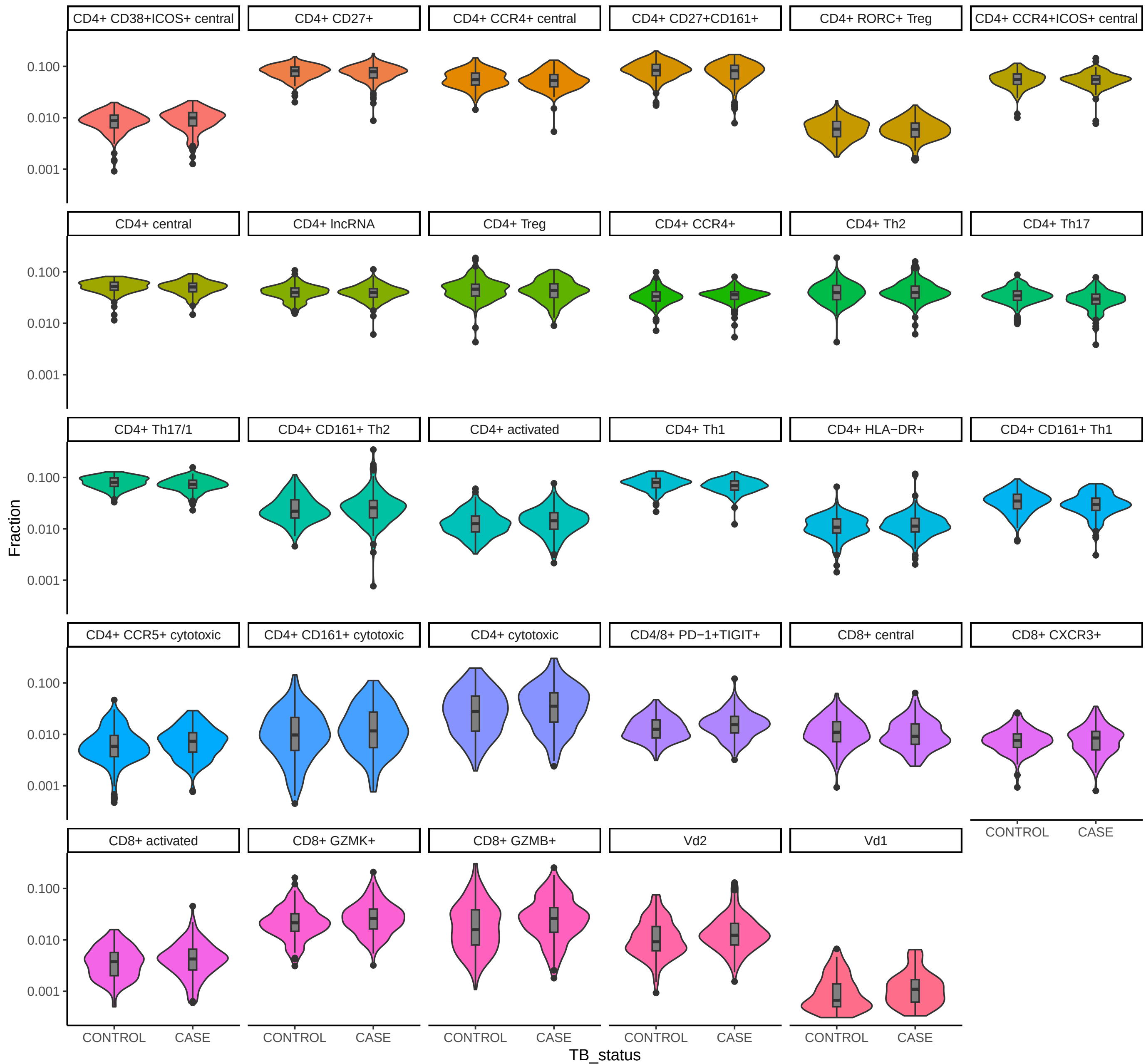

Supplementary Figure 9: Tuberculosis infection in T cell subpopulations. Observed fractions for each cell subpopulation stratified by case vs control status. Boxplot indicates mean, 25% and 75% quantile, and +/− 1.5 * interquartile range.

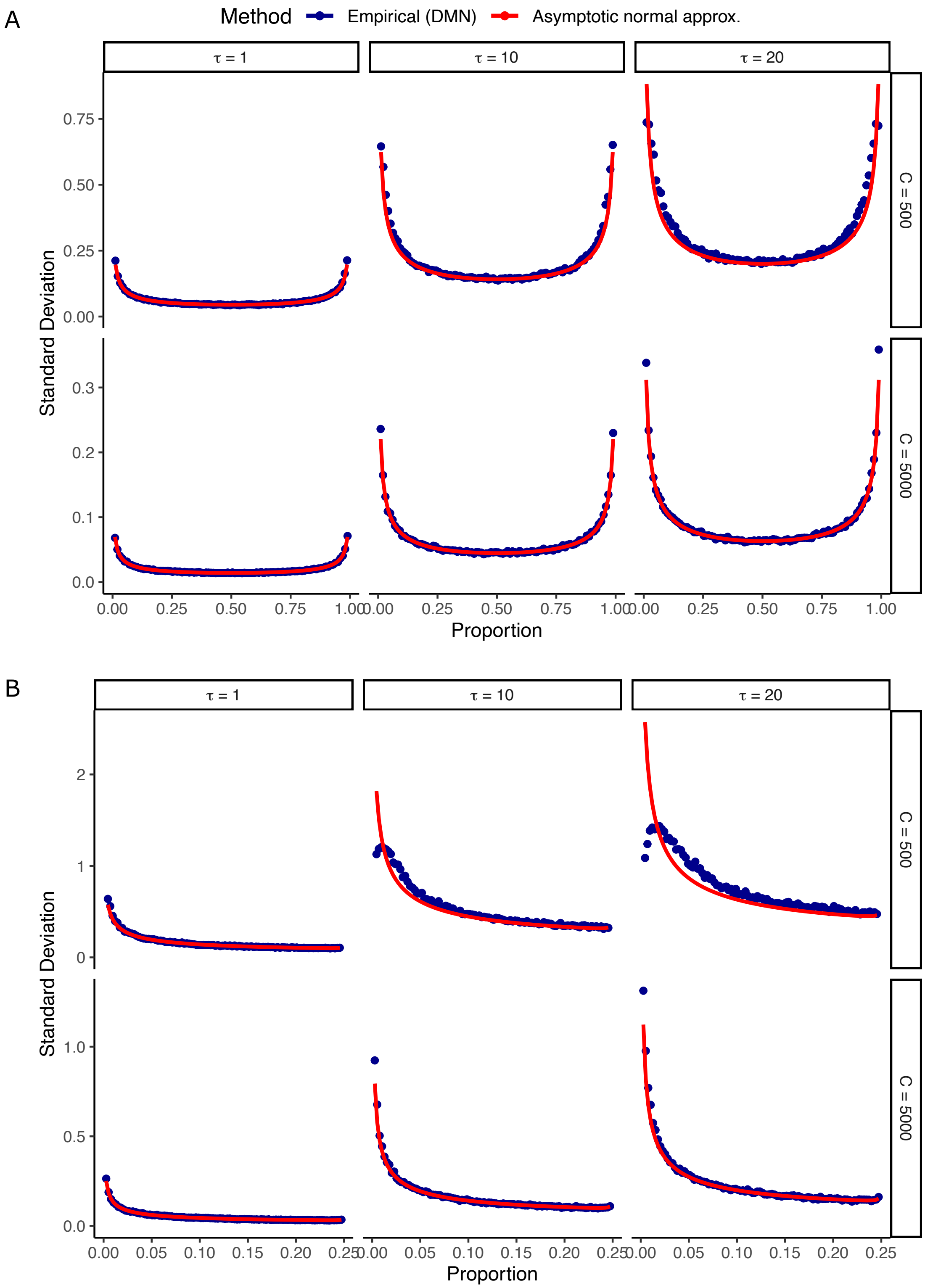

Supplementary Figure 10: Performance of asymptotic normal approximation. Standard deviation of the CLR transformed proportions from 1000 samples drawn from a Dirichlet multinomial model (blue points) are compared to the normal approximation developed here (red line) when at least 2 counts are observed. Data was simulated using an overdispersion $\tau \in \{1, 10, 20\}$ and total counts $C \in \{500, 5000\}$. Results are shown for **A)** $D = 2$ categories and **B)** $D = 15$ categories.

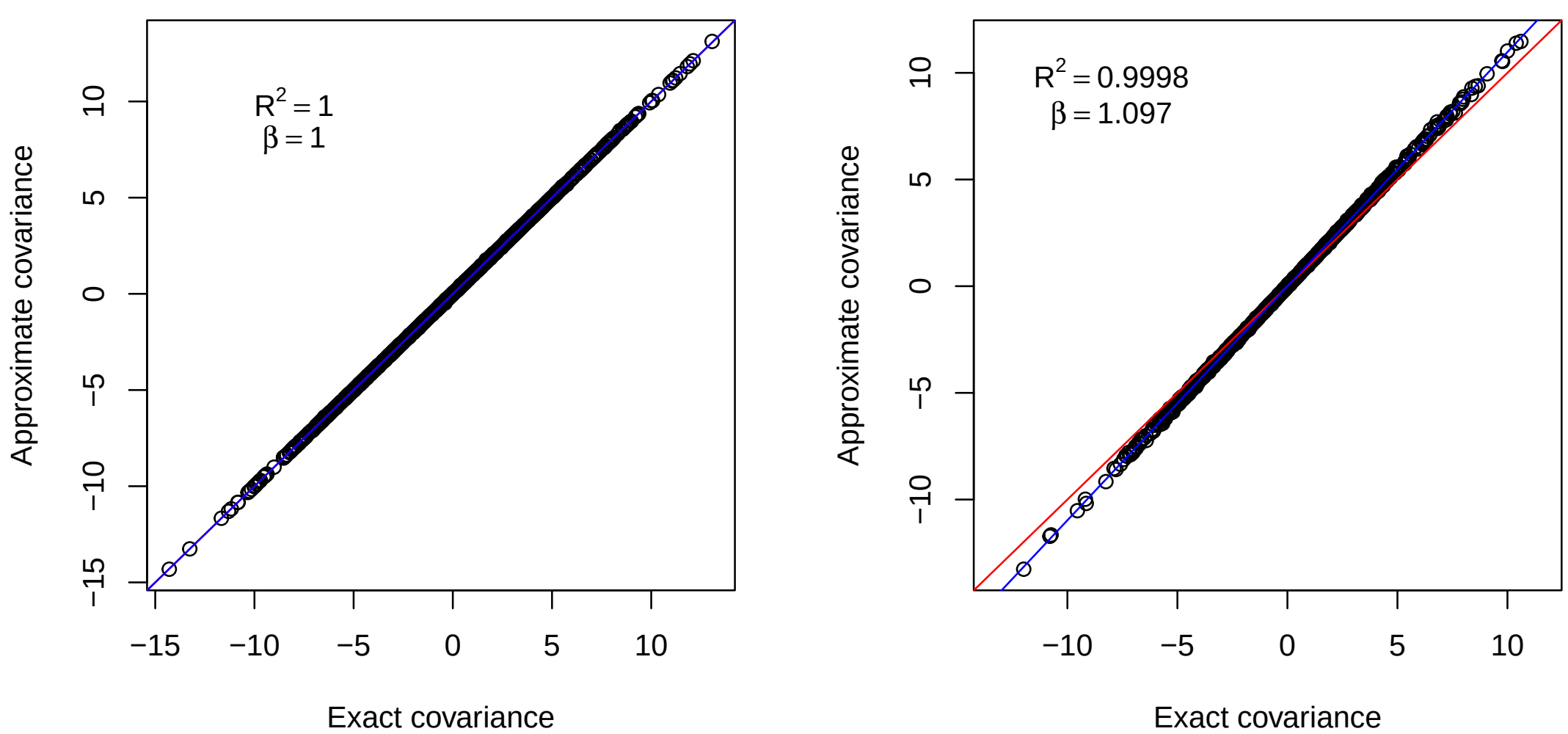

Supplementary Figure 11: Comparison of variance methods. Compare exact versus approximate methods to compute covariance for a fixed effects model using 200 samples and 1000 simulations. Left: weights are shared across responses. Right: weights vary across responses. Red line is diagonal, blue line is the least squares fit with coefficient β.

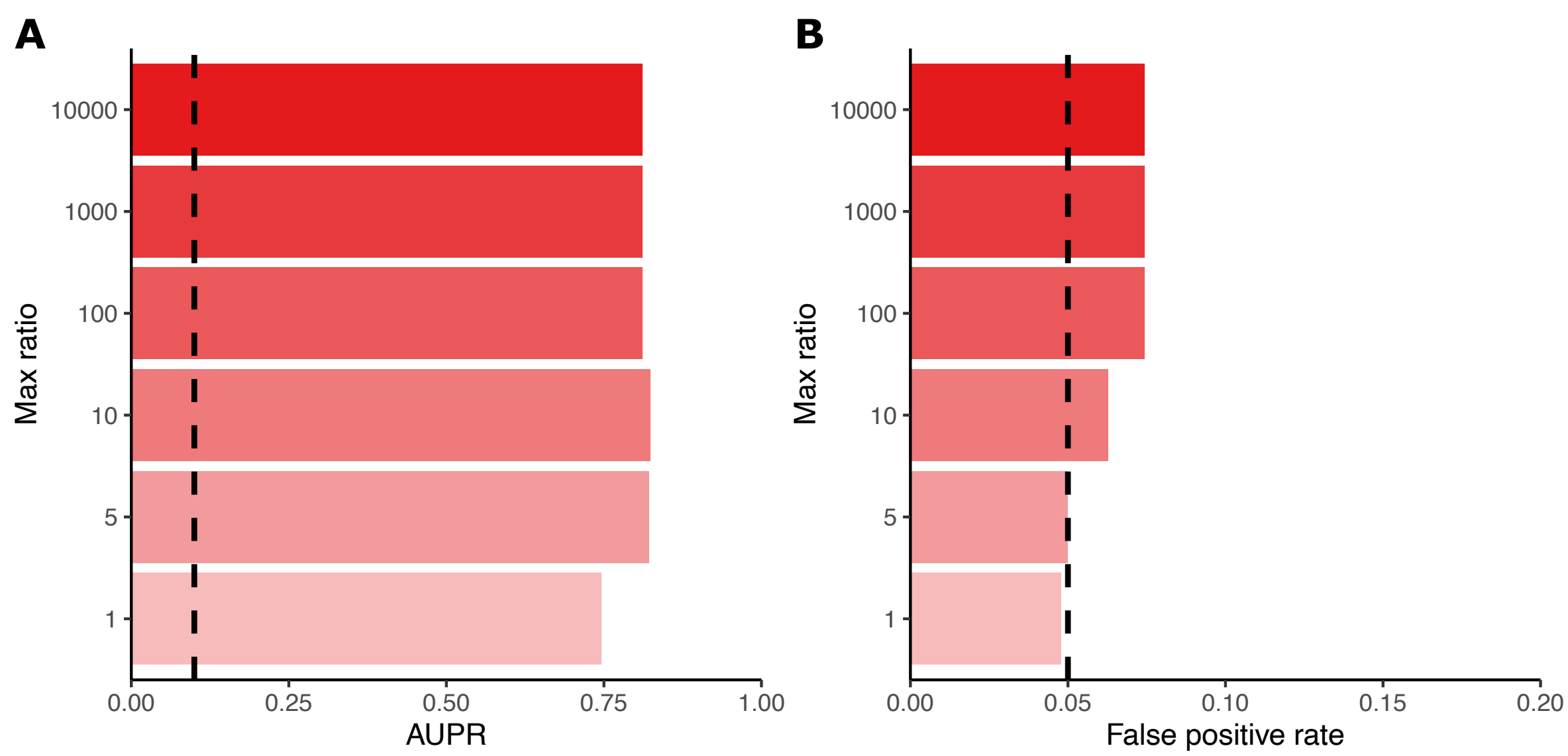

Supplementary Figure 12: Sensitivity analysis: weight regularization. Simulations from Fig 2A were repeated changing the weight regularization parameter value for crumblr. This parameter caps the ratio between the maximum value and specified quantile value (default of 5%). **A)** AUPR and **B)** false positive rate are shown for 10k simulations. With max.ratio set to 1, there is very little variation in the precision weights and the method behaves like standard CLR. With large values for max.ratio, stochastic variation in the precision weights can give very high weights to a small subset of samples. This can increase the false positive rate and underscores the need to regularize the weights.

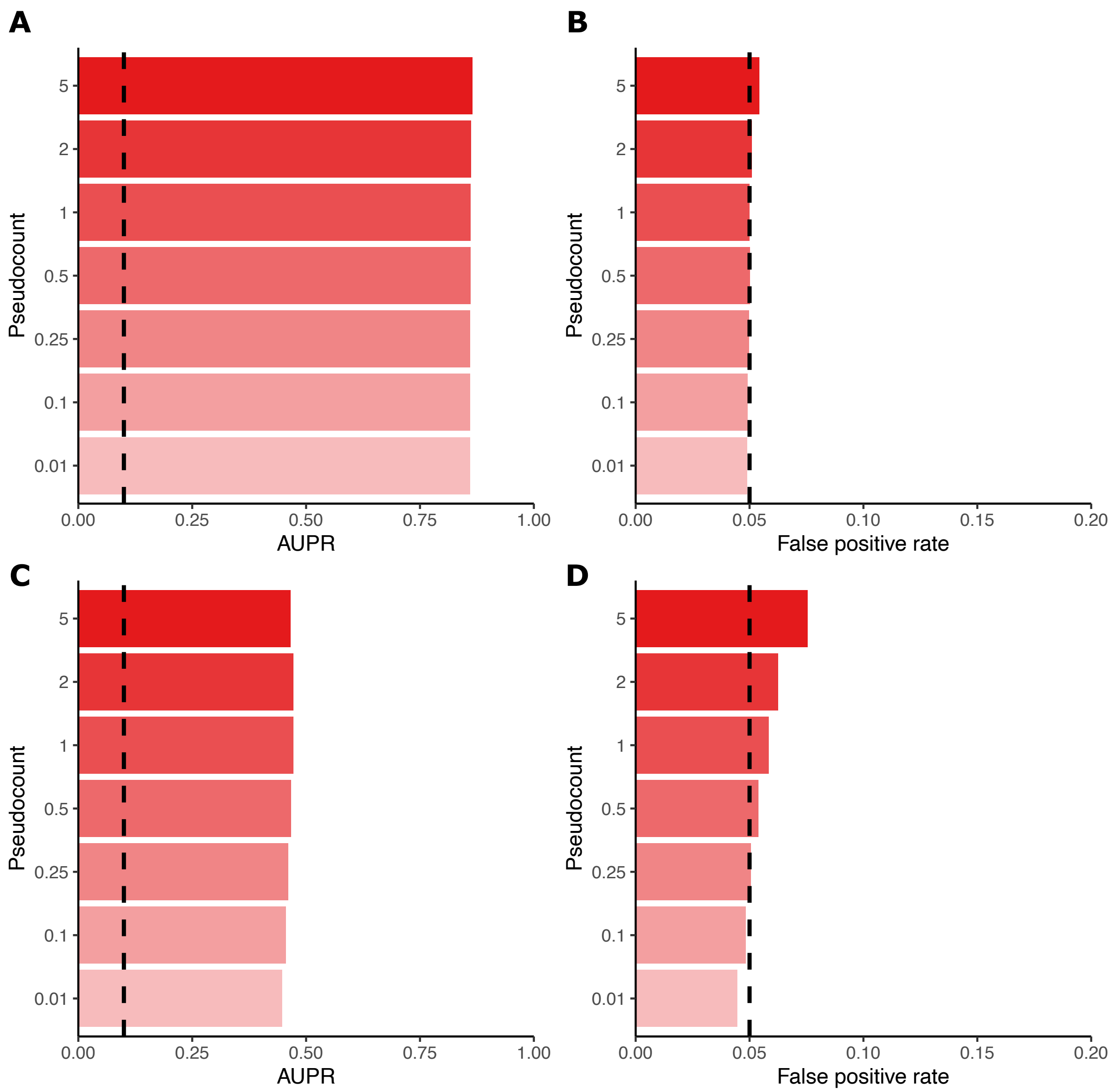

Supplementary Figure 13: Sensitivity analysis: pseudocount. Simulations from Fig 2A were repeated, changing the pseudocount value for crumblr. Results are shown for 10k simulations. **A,B)** AUPR and false positive rate. **C,D)** AUPR and false positive rate for simulations, reducing the total number of cells from 2000 to 200 to consider conditions where the pseudocount has a larger effect on results.

Supplementary Methods

1 Count ratio uncertainty modeling with linear regression

Here we propose count ratio uncertainty modeling based linear regression (`crumblr`). When considering count ratios for multiple variables, it is desirable to compare all variables to the same baseline. Selecting the last variable as the baseline is simple, but the count ratios will depend on the ordering of the variables. Instead, Aitchison (1986) proposes the centered log ratio (clr) using the geometric mean of the counts across all variables as the common baseline:

$$\mathrm{clr}(p_i) = \log(p_i) - \frac{1}{D}\sum_{j=1}^{D}\log(p_j) \tag{1}$$

where p_i is the proportion of the ith out of D components.

In any count ratio data, there are two sources of heteroskedasticity:

1) for proportions, the sample variance is related to the value. This is clearly seen with the beta distribution
2) the proportions have different count totals in the denominator.

Here, we approximate the sampling variance of the proportions after the clr transform.

1.1 Isometric log ratio

Since proportions are constrained to sum to 1, proportions divided into D variables actually span a $D-1$ dimensional space. This is also true for clr transformed proportions. This degenerate set of variables can be problematic in statistical modeling since the covariance matrix across clr transformed proportions is not invertible. To address this Egozcue et al. (2003) proposed the isometric log ratio (ilr) to map the clr transformed variables into $D-1$ variables. Importantly, the mapping between clr and ilr coordinates is a linear operation using an orthonormal transform.

Egozcue et al. (2003) construct the orthonormal matrix V and define the ilr coordinates as

$$\text{ilr}(\mathbf{p}) = \mathbf{V}^T \text{clr}(\mathbf{p}), \tag{2}$$

so that distances in clr space are preserved in the ilr space. By construction $\mathbf{V}$ is a $D \times D-1$ matrix thats satisfies properties

$$\mathbf{V}^T\mathbf{V} = \mathbf{I}_{D-1} \tag{3}$$

$$\mathbf{V}\mathbf{V}^T = \mathbf{I}_D - \frac{1}{D}\mathbf{1}\mathbf{1}^T \tag{4}$$

$$\mathbf{V}^+ = \mathbf{V}^T \tag{5}$$

where $\mathbf{I}_D$ is the $D \times D$ identify matrix, $\mathbf{1}$ is a vector of D 1's, and $\mathbf{V}^+$ indicates the Moore–Penrose inverse. It follows that the inverse projection from ilr coordinates back to clr coordinates is

$$\text{clr}(\mathbf{p}) = \mathbf{V}\,\text{ilr}(\mathbf{p}) \tag{6}$$

Populating $\mathbf{V}$ with numeric values that satisfy these properties is quite complicated and is described in detail by Egozcue et al. (2003). But understanding these details doesn't add to the intuition about the current work. Importantly, we note that our work uses $\mathbf{V}$ in derivations, but, due to its special properties, all instances of the matrix cancel out so it never needs to be instantiated.

1.2 Modeling sampling variance in clr coordinates

Regression models fit using clr transformed variables as responses are easily interpretable since each clr variable corresponds to an original variable. Conversely, ilr transformed variables have nice mathematical properties, but are challenging to interpret because they are linear combinations of multiple clr variables (Pawlowsky-Glahn et al., 2015). It is common to include clr and ilr transformed variables as responses in regression models. However, standard application does not account for the substantial difference in measurement precision across observations. The standard assumption of equal measurement precision in regression models can cause loss of power and poor control of the false positive rate.

Here, we take advantage of the interpretability of the clr transform and the mathematical properties of the ilr transform described above to explicitly model measurement uncertainty in observed counts with precision weights.

1.2.1 Asymptotic normal approximation of multinomial counts

Let $\mathbf{p}$ be the true vector of proportions for D components with p_i being the true proportion for the ith component. Let $\mathbf{c}$ be the vector of observed counts drawn from a multinomial distribution with n total counts. The expected number of counts is $E[\mathbf{c}] = n\mathbf{p}$, the maximum likelihood estimate of the proportions is $\hat{\mathbf{p}} = \mathbf{c}/n$, and the variance of the counts is $n\left[\text{diag}(\mathbf{p}) - \mathbf{p}\mathbf{p}^T\right]$. Based asymptotic properties of multinomial distribution, for large n the distribution of the estimated proportions converges to a multivariate normal distribution

$$\hat{\mathbf{p}} \xrightarrow{d} \mathcal{N}\left(\mathbf{p}, \frac{1}{n}\left[\text{diag}(\mathbf{p}) - \mathbf{p}\mathbf{p}^T\right]\right). \tag{7}$$

Following Egozcue et al. (2020) and Graffelman et al. (2015), we use the delta method to obtain the asymptotic normal distribution of the ilr transformed estimated proportions. Consider a transformation $g(.)$ applied to any vector of random variables $\mathbf{x}$ with covariance $\boldsymbol{\Sigma}_{\mathbf{x}}$, then the asymptotic variance of the transformed random variable is

$$\left(\frac{\partial g(\mathbf{x})}{\partial \mathbf{x}}\right) \boldsymbol{\Sigma}_x \left(\frac{\partial g(\mathbf{x})}{\partial \mathbf{x}}\right)^T. \tag{8}$$

Applying this to the ilr transform gives

$$\begin{aligned} \frac{\partial\,\text{ilr}(\mathbf{p})}{\partial \mathbf{p}} &= \frac{\partial \mathbf{V}^T \log(\mathbf{p})}{\partial \mathbf{p}} & (9) \\ &= \mathbf{V}^T \text{diag}(1/\mathbf{p}) & (10) \end{aligned}$$

so that

$$\begin{aligned} \text{var}[\text{ilr}(\hat{\mathbf{p}})] &= n^{-1}\left[\mathbf{V}^T\text{diag}(1/\mathbf{p})\right]\left[\text{diag}(\mathbf{p}) - \mathbf{p}\mathbf{p}^T\right]\left[\text{diag}(1/\mathbf{p})\mathbf{V}\right] & (11) \\ &= n^{-1}\mathbf{V}^T\text{diag}(1/\mathbf{p})\mathbf{V} - \mathbf{V}^T\mathbf{1}\mathbf{1}^T\mathbf{V} & (12) \\ &= n^{-1}\mathbf{V}^T\text{diag}(1/\mathbf{p})\mathbf{V} & (13) \end{aligned}$$

since $\mathbf{V}^T\mathbf{1} = 0$. The asymptotic normal approximation of the ilr transform is then

$$\text{ilr}(\hat{\mathbf{p}}) \rightarrow \mathcal{N}\left(\text{ilr}(n\mathbf{p}), n^{-1}\mathbf{V}^T\text{diag}(1/\mathbf{p})\mathbf{V}\right). \tag{14}$$

Given the particular form of the variance-covariance matrix in ilr space, we derive the variance-covariance matrix in clr space using the projection matrix $\mathbf{V}$ mapping between the two spaces. Using

the properties of linear algebra, variance, and the special form of $\mathbf{V}$, we see that

$$\begin{aligned}
\text{var}[\text{clr}(\hat{\mathbf{p}})] &= \mathbf{V}\,\text{var}[\text{ilr}(\hat{\mathbf{p}})]\mathbf{V}^T && (15)\\
&= n^{-1}\mathbf{V}\mathbf{V}^T\text{diag}(1/\mathbf{p})\mathbf{V}\mathbf{V}^T && (16)\\
&= n^{-1}\left(\mathbf{I}_D - D^{-1}\mathbf{1}\mathbf{1}^T\right)\text{diag}(1/\mathbf{p})\left(\mathbf{I}_D - D^{-1}\mathbf{1}\mathbf{1}^T\right)^T && (17)\\
&= n^{-1}\left(\text{diag}(1/\sqrt{\mathbf{p}}) - D^{-1}\mathbf{1}\mathbf{1}^T\text{diag}(1/\sqrt{\mathbf{p}})\right)\left(\text{diag}(1/\sqrt{\mathbf{p}}) - D^{-1}\mathbf{1}\mathbf{1}^T\text{diag}(1/\sqrt{\mathbf{p}})\right)^T && (18)\\
&= n^{-1}\left[\text{diag}(1/\mathbf{p}) - 2D^{-1}\text{diag}(1/\sqrt{\mathbf{p}})\mathbf{1}\mathbf{1}^T\text{diag}(1/\sqrt{\mathbf{p}}) + D^{-2}\mathbf{1}\mathbf{1}^T\text{diag}(1/\mathbf{p})\mathbf{1}\mathbf{1}^T\right]. && (19)
\end{aligned}$$

This describes the full $D \times D$ asymptotic variance-covariance matrix of the clr-transformed proportions. The sampling variance of the ith component is

$$\text{var}[\text{clr}(\hat{p}_i)] = \frac{1}{n}\left[\frac{1}{p_i} - \frac{2}{Dp_i} + \frac{1}{D^2}\sum_{j=1}^{D}\frac{1}{p_j}\right]. \tag{20}$$

For observed data, the maximum likelihood estimate of the proportion can be substituted for the true value to obtain an estimate of the variance

$$\widehat{\text{var}[\text{clr}(\hat{p}_i)]} = \frac{1}{n}\left[\frac{1}{\hat{p}_i} - \frac{2}{D\hat{p}_i} + \frac{1}{D^2}\sum_{j=1}^{D}\frac{1}{\hat{p}_j}\right]. \tag{21}$$

Note that the variance is a function of the total number of counts, number of components, and the proportions for each component, but the projection matrix $\mathbf{V}$ cancels out. This gives a simple formula for the sampling variance of each value in clr space.

An alternative derivation

During revision, we found a simpler derivation of the same result that avoids the ilr transform. Using vector notation for the proportions, we can restate the clr function from Equation (1) and as

$$\text{clr}(\mathbf{p}) = \left(\mathbf{I}_D - \frac{1}{D}\mathbf{1}\mathbf{1}^T\right)\log(\mathbf{p}). \tag{22}$$

Applying the delta method directly to to this transform, we see that

$$\begin{aligned}
\frac{\partial\,\text{clr}(\mathbf{p})}{\partial\mathbf{p}} &= \left(\mathbf{I}_D - \frac{1}{D}\mathbf{1}\mathbf{1}^T\right)\frac{\partial\log(\mathbf{p})}{\partial\mathbf{p}} && (23)\\
&= \left(\mathbf{I}_D - \frac{1}{D}\mathbf{1}\mathbf{1}^T\right)\text{diag}(1/\mathbf{p}) && (24)
\end{aligned}$$

so that

$$\text{var}[\text{clr}(\hat{\mathbf{p}})] = n^{-1}\left(\mathbf{I}_D - \frac{1}{D}\mathbf{1}\mathbf{1}^T\right)\text{diag}(1/\mathbf{p})\left(\mathbf{I}_D - \frac{1}{D}\mathbf{1}\mathbf{1}^T\right)^T, \tag{25}$$

which is equivalent to Equation (17).

1.2.2 Asymptotic normal approximation to a Dirichlet-multinomial

The Dirichlet-multinomial (DMN) distribution is a more flexible model of multi-class count data that generalizes the multinomial distribution. While the multinomial distribution assumes the underlying proportions are fixed, in the DMN the proportions are random variables drawn from a Dirichlet distribution. Counts from a DMN model can be drawn according to

$$\mathbf{c} \sim Multinomial(n, \mathbf{p}) \tag{26}$$
$$\mathbf{p} \sim Dirichlet(\boldsymbol{\alpha}) \tag{27}$$

where $\boldsymbol{\alpha}$ is the vector of parameters for Dirichlet distribution so that the expected proportion of category i is α_i/α_0 where $\alpha_0 = \sum_k \alpha_k$. Following standard results for the DMN, the sampling variance of the observed counts is

$$\text{var}(\mathbf{c}) = n \left[\text{diag}(\mathbf{p}) - \mathbf{p}\mathbf{p}^T\right] \left(\frac{n + \alpha_0}{1 + \alpha_0}\right). \tag{28}$$

Letting $\tau = (n + \alpha_0)/(1 + \alpha_0)$, then

$$\text{var}(\mathbf{c}) = n \left[\text{diag}(\mathbf{p}) - \mathbf{p}\mathbf{p}^T\right] \tau. \tag{29}$$

Based on this, $\tau = 1$ corresponds to the multinomial distribution, and τ can be interpreted as the overdispersion parameter that increases the variance of the counts while keeping their expected values unchanged.

Since the overdispersion τ is a scalar parameter shared by all categories, the derivation from the previous section can be adapted to show that the proportions are asymptotically normal according to

$$\hat{\mathbf{p}} \xrightarrow{d} \mathcal{N}\left(\mathbf{p}, \frac{\tau}{n}\left[\text{diag}(\mathbf{p}) - \mathbf{p}\mathbf{p}^T\right]\right), \tag{30}$$

so that the asymptotic variance of the clr-transformed proportions is

$$\widehat{\text{var}[\text{clr}(\hat{p}_i)]} = \frac{\hat{\tau}}{n}\left[\frac{1}{\hat{p}_i} - \frac{2}{D\hat{p}_i} + \frac{1}{D^2}\sum_{j=1}^{D}\frac{1}{\hat{p}_j}\right]. \tag{31}$$

This approximation is quite accurate even for small fractions when a sufficient number of total cells are observed (**Supplementary Figure 10**).

On the special form of the variance in DMN model
In the DMN model the overdispersion parameter, τ, is constant across samples and components. Moreover, the variance of the clr-transformed proportions is a multiplicative function of the overdispersion parameter. While this can have some drawbacks compared to more flexible models where overdispersion can vary across samples and components, the form of the variance of the clr-transformed proportions allows a uniquely simple way to model it with a weighted regression model.

In the case of heteroskedastic errors (see Section 1.3.1 below), fitting a regression weighting samples proportional to the inverse of the variance for each sample gives consistent estimates of the coefficients (Carroll and Ruppert, 2017). Considering a single response variable, let w_k indicate the relative weight and y_k be the observed value of the kth sample and so that

$$w_k \;\; \propto \;\; 1/\mathrm{var}(y_k). \tag{32}$$

In the case of clr-transformed proportions, let $w_{i,k}$ be the weight and $v_{i,k}$ be the sample variance of the ith component for the kth sample computed from Eqn (31), so that

$$w_{i,k} \;\; \propto \;\; 1/v_{i,k}. \tag{33}$$

Since the weights must only be *proportional* to the inverse variance, only the *relative* variances matter. If the value of $\hat{\tau}$ were estimated from the data, it would cancel out in computing the relative weights. It follows that the relative weights are thus invariant to the value of $\hat{\tau}$. Therefore, estimates of the coefficients and their covariance, as well as hypothesis testing does not depend on the value of $\hat{\tau}$, and it does not need to be estimated from the data.

Combining the single overdispersion term of the DMN model shared across samples with the scale-invariant weighted regression removes the need to estimate $\hat{\tau}$ from the data. Of course, the *true* value of τ affects the observed data, coefficient estimates, etc., but these are invariant to the *estimated* value of τ.

According to Theorem 4.1 (see Section 4), in a weighted least squares regression model the coefficient estimates and their variances are invariant to the scale of the weights. Therefore, by convention, the weights are scaled to sum to 1.

1.3 Multivariate modeling of cell type components

Consider a multivariate regression with n samples, c variables, and m response values using design matrix $\mathbf{X}_{n\times c}$ and responses stored as column in the matrix $\mathbf{Y}_{n\times m}$. In the typical homoskedastic model, where for a given response vector the error variance is the same for each sample, an unweighted regression gives consistent estimates of the coefficients. This corresponds to the case of regression weights all equal to one, and standard results give estimated coefficients

$$\hat{\mathbf{B}} = \left(\mathbf{X}^T\mathbf{X}\right)^{-1}\mathbf{X}^T\mathbf{Y}. \tag{34}$$

Letting the matrix of residuals be

$$\mathbf{R} = \mathbf{Y} - \mathbf{X}\hat{\mathbf{B}}, \tag{35}$$

the covariance matrix of the coefficient estimates is

$$\mathrm{var}(\mathrm{vec}(\hat{\mathbf{B}})) = \mathbf{C} \otimes \left(\mathbf{X}^T\mathbf{X}\right)^{-1} \tag{36}$$

where $\mathbf{C} = \mathbf{R}^T\mathbf{R}/\nu$, ν is the residual degrees of freedom, the vec(.) operator converts a matrix to a column vector, and $\otimes$ is the Kronecker product.

Examining covariance between coefficients from response i and j gives

$$\text{cov}\left(\hat{\beta}_i, \hat{\beta}_j\right) = C_{i,j}\left(\mathbf{X}^T\mathbf{X}\right)^{-1} \tag{37}$$

$$= \text{cor}(r_i, r_j)\left(\mathbf{X}^T\mathbf{X}\right)^{-1}\hat{\sigma}_i\hat{\sigma}_j. \tag{38}$$

where $C_{i,j} = r_i^T r_j/\nu$, r_i is the ith column of $\mathbf{R}$, $\hat{\sigma}_i$ and $\hat{\sigma}_j$ are the estimated standard deviations of r_i and r_j, respectively, and $\hat{\beta}_i$ is the ith column of $\hat{\mathbf{B}}$.

1.3.1 Weighted linear model

In the heteroskedastic model, where for a given response vector the error variance varies across samples, an unweighted regression no longer gives consistent estimates of the coefficients. Instead, a weighted regression gives consistent estimates if the weights are set to be proportional to the inverse of the variance for each sample (Carroll and Ruppert, 2017). This is often termed 'precision-weighting'. Letting $w^{(i)}$ be the vector of weights for response i, the consistency condition is satisfied when

$$w^{(i)} \propto 1/var(y_i). \tag{39}$$

This means that only the *relative* weights enter the model. Knowing the correct scale of the weights does not provide additional information for the purpose of estimating regression coefficients or performing hypothesis testing. Therefore, we use the relative weights $w^{(i)}/\sum_i w^{(i)}$ in all models but for notational simplicity we use $w^{(i)}$ to refer to these normalized weights throughout.

Letting $\mathbf{W}^{(i)} = \text{diag}\left(w^{(i)}\right)$ and y_i be the vector storing response i, then

$$\hat{\beta}_i = \left(\mathbf{X}^T\mathbf{W}^{(i)}\mathbf{X}\right)^{-1}\mathbf{X}^T\mathbf{W}^{(i)}y_i. \tag{40}$$

Creating a weighted version of any matrix or vector according to $\tilde{\mathbf{M}}_i = \mathbf{M}\text{diag}\left(\sqrt{w^{(i)}}\right)$, simplifies the notation so that the estimated coefficients are

$$\hat{\beta}_i = \left(\tilde{\mathbf{X}}_i^T\tilde{\mathbf{X}}_i\right)^{-1}\tilde{\mathbf{X}}_i^T\tilde{y}_i, \tag{41}$$

and the covariance between coefficients estimates is now

$$\text{cov}\left(\hat{\beta}_i, \hat{\beta}_j\right) = \tilde{\mathbf{C}}_{i,j}\left(\tilde{\mathbf{X}}_i^T\tilde{\mathbf{X}}_i\right)^{-1}\left(\tilde{\mathbf{X}}_i^T\tilde{\mathbf{X}}_j\right)\left(\tilde{\mathbf{X}}_j^T\tilde{\mathbf{X}}_j\right)^{-1}. \tag{42}$$

where $\tilde{\mathbf{C}} = \tilde{r}_i^T\tilde{r}_j/\nu$. When all weights equal 1, these reduce to the standard equations (34) and (34).

1.3.2 Weighted linear mixed model

For the linear mixed model, closed form estimates don't exist and parameters are estimated using numerical optimization in `lme4` (Bates et al., 2015). Here we build up to the covariance estimates starting at the unweighted linear model. Let $\hat{\mathbf{\Sigma}}_i$ be the estimated covariance matrix between coefficients for response i. In the case of standard, unweighted linear regression, $\hat{\mathbf{\Sigma}}_i$ has the standard form

$$\hat{\mathbf{\Sigma}}_i = \left(\mathbf{X}^T\mathbf{X}\right)^{-1}\hat{\sigma}_e^2. \tag{43}$$

Incorporating weights and using the notation as above, the estimator becomes

$$\hat{\mathbf{\Sigma}}_i = \left(\tilde{\mathbf{X}}_i^T\tilde{\mathbf{X}}_i\right)^{-1}\tilde{r}_i^T\tilde{r}_i/\nu, \tag{44}$$

and equals the previous form when all weights are 1.

In the unweighted linear mixed model,

$$y = \mathbf{X}\beta + \mathbf{Z}u + \varepsilon \tag{45}$$
$$u = \mathcal{N}(0, \mathbf{G}) \tag{46}$$
$$\varepsilon = \mathcal{N}(0, \mathbf{I}\sigma^2), \tag{47}$$

with the variance parameters in $\mathbf{G}$, the covariance between estimated coefficient is

$$\hat{\mathbf{\Sigma}}_i = \left(\mathbf{X}^T\mathbf{V}_i^{-1}\mathbf{X}\right)^{-1} \tag{48}$$
$$\mathbf{V}_i = \mathbf{Z}\mathbf{G}_i\mathbf{Z}^T + \mathbf{I}\hat{\sigma}_i^2. \tag{49}$$

Extending this to the case of the weighted linear mixed model gives

$$\hat{\mathbf{\Sigma}}_i = \left(\tilde{\mathbf{X}}_i^T\mathbf{V}_i^{-1}\tilde{\mathbf{X}}_i\right)^{-1} \tag{50}$$
$$\mathbf{V}_i = \tilde{\mathbf{Z}}_i\mathbf{G}_i\tilde{\mathbf{Z}}_i^T + \mathbf{I}\hat{\sigma}_i^2. \tag{51}$$

In Equation (42), we stated the covariance in terms of $\tilde{\mathbf{X}}_i$ and $\tilde{\mathbf{X}}_j$ by implicitly using the result of Equation (44). But in the case of the weighted linear mixed model, $\hat{\mathbf{\Sigma}}_i$ is more complicated.

Using the form of Equation (38), the covariance in coefficient estimates between responses i and j can be stated in terms of the covariance for each response and the correlation between residuals when all weights are 1. When weights vary, the covariance between coefficients estimates for fixed effects in the linear mixed model is approximated as

$$\text{cov}\left(\hat{\beta}_i, \hat{\beta}_j\right) = \text{cor}(\tilde{r}_i, \tilde{r}_j)\hat{\mathbf{\Sigma}}_i\left(\hat{\mathbf{\Sigma}}_i^{-\frac{1}{2}}\hat{\mathbf{\Sigma}}_j^{-\frac{1}{2}}\right)\hat{\mathbf{\Sigma}}_j \tag{52}$$

When $i = j$, this formula reduces to the $\hat{\mathbf{\Sigma}}_i$. In the case of linear regression, when the weights vary across samples but are shared across responses, this method is equivalent to Equation (38).

With linear regression and weights varying across samples and responses, we can compare the exact covariance from Equation (38) with our approximation in Equation (52). In the case of general weights, simulations indicate that this approximation produces accurate estimates of the covariance in coefficient estimates across responses (**Supplementary Figure 11**). This suggests that the approximation is also good in the case of linear mixed model, even though we can't compare against the exact covariance in that case.

1.3.3 Multivariate hypothesis testing

Here, we develop a test statistic assuming an idealized case where the covariance matrix between parameter estimates is *known*. In the next section, we extend this to the realistic case of an *estimated* covariance matrix. Given a vector of coefficient values with one estimated for each response, we propose a hypothesis test comparing the mean coefficient value to a null value of zero. Let $\hat{\boldsymbol{\gamma}} = (\hat{\gamma}_1, ..., \hat{\gamma}_K)$ be the vector of estimated coefficients from across K responses and $\boldsymbol{\Omega}$ be the *known* covariance matrix, so that

$$\hat{\boldsymbol{\gamma}} \sim \mathcal{N}\left(\mu_\gamma, \boldsymbol{\Omega}\right). \tag{53}$$

Following Lin and Sullivan (2009), we test the null hypothesis that $\mu_\gamma = 0$ using maximum likelihood to obtain

$$\hat{\mu}_\gamma = \frac{\mathbf{1}^T \boldsymbol{\Omega}^{-1} \hat{\boldsymbol{\gamma}}}{\mathbf{1}^T \boldsymbol{\Omega}^{-1} \mathbf{1}} \tag{54}$$

$$\text{var}(\hat{\mu}_\gamma) = \frac{1}{\mathbf{1}^T \boldsymbol{\Omega}^{-1} \mathbf{1}}, \tag{55}$$

with the corresponding z-score

$$\frac{\hat{\mu}_\gamma}{\sqrt{\text{var}(\hat{\mu}_\gamma)}} = \frac{\mathbf{1}^T \boldsymbol{\Omega}^{-1} \hat{\boldsymbol{\gamma}}}{\sqrt{\mathbf{1}^T \boldsymbol{\Omega}^{-1} \mathbf{1}}}. \tag{56}$$

This method combines effect size estimates across multiple responses while modeling their dependency structure using the covariance matrix $\boldsymbol{\Omega}$. When the covariance matrix $\boldsymbol{\Omega}$ is known, rather than being estimated from the data, the test statistic is normally distributed. We apply this method to multivariate regression from above by setting $\hat{\boldsymbol{\gamma}}$ to the vector of estimated coefficients across responses from linear or linear mixed model.

1.3.4 An adaptation for unknown variance

In real data, the covariance matrix between coefficients must be estimated from the data according to Equations (42) or (52). Here, we extend the test statistic to the case of *estimated* covariance.

Consider the simple linear model with n samples and only a mean term,

$$\begin{aligned} y &= \mu + \varepsilon \quad (57) \\ \varepsilon &\sim \mathcal{N}(0, \sigma^2), \quad (58) \end{aligned}$$

where the estimated residual variance is

$$\hat{s}^2 = \sum_i (y_i - \hat{\mu})^2 / \nu, \quad (59)$$

with $\nu = n - 1$ residual degrees of freedom. Standard theory shows that

$$\frac{\nu \hat{s}^2}{\sigma^2} \sim \chi^2_\nu, \quad (60)$$

and the z-score has a Student-t distribution

$$\frac{\hat{\mu}}{\hat{s}/\sqrt{n}} \sim t_\nu. \quad (61)$$

This statistic has a Student-t distribution since the residual variance is unknown and is estimated from the data.

Extending this result to the multivariate case, the residual sum of squares matrix, $\mathbf{R}^T\mathbf{R}$, is drawn from a Wishart distribution according to

$$\mathbf{R}^T\mathbf{R} \sim Wishart(\mathbf{\Sigma}, \nu), \quad (62)$$

where $\mathbf{\Sigma}$ is the true error covariance matrix, and ν is the residual degrees of freedom.

We can now approximate the null distribution by sampling covariance matrices from a Wishart distribution centered at the observed residual sum of squares matrix according to

$$\begin{aligned} \hat{\mathbf{\Omega}} &\sim Wishart(\mathbf{R}^T\mathbf{R}, \nu) \quad (63) \\ \hat{\gamma} &\sim \mathcal{N}(0, \hat{\mathbf{\Omega}}). \quad (64) \end{aligned}$$

We use these sampled values to evaluate the test statistic in Equation (56) using Algorithm 1.

Algorithm 1 Monte Carlo sampling of the Lin-Sullivan statistic under the null

1) Sample residual sum of squares matrix

$\mathbf{\Omega}_1 \sim Wishart(\mathbf{R}^T\mathbf{R}, \nu)$

2) Sample vector of regression coefficients using an independent sample of the residual sum of squares matrix

$\mathbf{\Omega}_2 \sim Wishart(\mathbf{R}^T\mathbf{R}, \nu)$

$\boldsymbol{\gamma} \sim \mathcal{N}(0, \mathbf{\Omega}_2)$

3) Compute z-statistic

$$z = \frac{\mathbf{1}^T\mathbf{\Omega}_2^{-1}\hat{\boldsymbol{\gamma}}}{\sqrt{\mathbf{1}^T\mathbf{\Omega}_1^{-1}\mathbf{1}}} \tag{65}$$

Let z_i be the ith of a total of κ values of the test statistic drawn from the null distribution, and z_{obs} be the observed values of the test statistic from the data. The p-value for a two-sided test can be computed by comparing z_{obs}^2 to z_i^2 according to

$$\frac{1 + \sum_{i=1}^{\kappa} z_{obs}^2 > z_i^2}{\kappa + 1}, \tag{66}$$

adding 1 to the numerator and denominator to avoid p-values of zero (Phipson and Smyth, 2010).

However, this raises two issues: 1) the smallest empirical p-value that can be computed is $1/(\kappa + 1)$, and 2) the sampling error of the p-value can be substantial for small p-values. This means that κ must be very large in order to produce small precise p-values. This can be very computationally expensive. In order to reduce the number of Monte Carlo samples needed, a gamma distribution is then fit to these draws from the null distribution, and an empirical p-value is computed from the cumulative distribution function of this gamma.

Residual degrees of freedom

In the case of a linear model, the residual degrees of freedom, ν, is computed from the sample size and the number of coefficients estimated. In the case of the a linear mixed model, the form of the residual degrees of freedom is more complicated. We use an approximation we developed previously (Hoffman et al., 2024). See Supplementary Methods Section 2.3.1 of that work for the full derivation. It is implemented in `variancePartition::rdf.merMod()` and computes the residuals degrees of freedom from a linear mixed model fit with `lme4::lmer()`.

2 Simulations

2.1 Univariate hypothesis testing

Counts were simulated from a Dirichlet-multinomial with a specified sample size, number of classes, overdispersion, effect size of the trait variable, and size of the batch effect. The trait of interest was normally distributed and samples were randomly assigned discrete batches. The coefficients for the effect of each batch were normally distributed with a mean of zero and a variance of $\gamma \in (0, 0.5, 1, 3)$. The linear predictor was created by multiplying the design matrix storing the trait and batch variables by the coefficient vector. The linear predictor for each sample was then exponentiated to obtain the fractions for each cell component. For each sample, the total number cell counts was drawn from a negative binomial with specified mean and $\theta = 3$ to give substantial variation in total cell counts as seen in real data. Counts were drawn from a Dirichlet-multinomial based on the probabilities, total count number per sample, and overdispersion.

2.2 Multivariate hypothesis testing

Results are shown for the fixed effect model incorporating correlation between test statistics (Lin and Sullivan, 2009), Fisher's method for combining p-values across tests ($-2\sum_{i=1}^{k} \log p_i \sim \chi^2_{2k}$ for k p-values), and Sidak's method for computing the minimum p-value corrected for multiple testing ($p_{\text{Sidak}} = 1 - (1 - \min(p))^k$). Simulations were performed 100k times with with 200 samples, 2 to 12 responses, heteroskedastic measurement error, and an error correlation between response variables of 0.8. 20k simulations used an effect sizes of 0.8 shared across responses, while the rest were simulated under the null model of no effect.

3 Implementation of other methods for comparison

Hypothesis testing for differential cell composition was performed with a range of existing methods that either model the cell fraction or directly modeled the number of observed counts. Let `y` be a vector storing the observed counts for a given cell type across all samples, `C` be a vector storing the total number of cells observed for each sample, and `x` be the variable of interest for hypothesis testing. Most models were fit in `R` as described in **Table S1** for fixed effects and **Table S2** for mixed effect modeling of batches. We note that the beta-binomial model does not supported mixed effects.

The centered log ratio (CLR) method was fit by performing the CLR transform using `clr()` in the `compositions` package available on CRAN and then fitting the regression models for each cell

type using `dream()` in the `variancePartition` package (Hoffman and Roussos, 2021, Hoffman and Schadt, 2016). Transformations involving log, logit and asin were computed this way too. The `crumblr()` function from the `crumblr` package was used to transform the count data and estimate the measurement precision of the transformed abundance values before being fed into `dream()`.

`scCODA` is a Bayesian method implemented in python (Büttner et al., 2021) and was called with default parameters using the observed cell counts and design matrix.

4 Additional proofs

Theorem 4.1. *In weighted least squares, the coefficient estimates and their variances are invariant to the scale of the weights.*

Consider a response y drawn from a multivariate normal distribution with mean determined by design matrix X of n samples and p variables, coefficients β and error term ε with zero mean. Consider a heteroskedastic model

$$y = X\beta + \varepsilon, \tag{67}$$

where error variance varies across samples, and let

$$\text{var}(\varepsilon_i) = 1/w_i \tag{68}$$

be the *true* error variance for sample i.

First, consider estimating the coefficients using weighted least squares. Using inverse variance weights by setting $W = \text{diag}(\vec{w})$ gives

$$\hat{\beta} = (X^T W^{-1} X)^{-1} X^T W^{-1} y. \tag{69}$$

Instead of using the *true* weights, consider scaling the weights by c and let β_c indicate that corresponding coefficient estimates. It follows that

$$\begin{aligned}
\hat{\beta}_c &= (X^T (cW)^{-1} X)^{-1} X^T (cW)^{-1} y & (70)\\
&= (X^T c^{-1} W^{-1} X)^{-1} X^T c^{-1} W^{-1} y & (71)\\
&= cc^{-1} (X^T W^{-1} X)^{-1} X^T W^{-1} y & (72)\\
&= (X^T W^{-1} X)^{-1} X^T W^{-1} y & (73)\\
&= \hat{\beta}. & (74)
\end{aligned}$$

Therefore the coefficient estimates are invariant to the scale of the weights.

Now consider the variance of the coefficient estimates. Letting $r = y - X\beta$, then

$$\hat{\sigma}^2 = r^T W^{-1} r/(n-p) \tag{75}$$

so that

$$\begin{aligned} \widehat{\text{var}}(\hat{\beta}) &= \hat{\sigma}^2 (X^T W^{-1} X)^{-1} && (76) \\ &= r^T W^{-1} r (X^T W^{-1} X)^{-1}/(n-p) && (77) \end{aligned}$$

Now consider scaling the weights by c, so that

$$\begin{aligned} \widehat{\text{var}}(\hat{\beta}) &= r^T (cW)^{-1} r (X^T (cW)^{-1} X)^{-1}/(n-p) && (78) \\ &= r^T c^{-1} W^{-1} r (X^T c^{-1} W^{-1} X)^{-1}/(n-p) && (79) \\ &= r^T c^{-1} W^{-1} r c (X^T W^{-1} X)^{-1}/(n-p) && (80) \\ &= r^T W^{-1} r (X^T W^{-1} X)^{-1}/(n-p) && (81) \end{aligned}$$

Therefore, the estimated covariance between coefficient estimates is invariant to the scaling of the weights.

■

Supplementary Tables

Method	Function	Formula	Arguments
Poisson	`glm()`	`y ~ offset(log(C)) + x`	`family=poisson()`
Negative binomial	`glm.nb()`	`y ~ offset(log(C)) + x`	
LM (fraction)	`lm()`	`y / C ~ x`	
LM (log)	`lm()`	`log(y / C) ~ x`	
LM (logit)	`lm()`	`logit(y / C) ~ x`	
LM (asin)	`lm()`	`asin(sqrt(y / C)) ~ x`	
Binomial	`glm()`	`cbind(y, C - y) ~ x`	`family=binomial()`
Beta-binomial	`betabin()`	`cbind(y, C - y) ~ x`	

Supplementary Table 1: Implementation of each method for fixed effects models

<table>
<tr><th>Method</th><th>Function</th><th>Formula</th><th>Arguments</th></tr>
<tr><td>Poisson</td><td><code>glmer()</code></td><td><code>y ~ offset(log(C)) + (1|Batch) + x</code></td><td><code>family=poisson()</code></td></tr>
<tr><td>Negative binomial</td><td><code>glmer.nb()</code></td><td><code>y ~ offset(log(C)) + (1|Batch)+ x</code></td><td></td></tr>
<tr><td>LM (fraction)</td><td><code>lmer()</code></td><td><code>y / C ~ (1|Batch) + x</code></td><td></td></tr>
<tr><td>LM (log)</td><td><code>lmer()</code></td><td><code>log(y / C) ~ (1|Batch) + x</code></td><td></td></tr>
<tr><td>LM (logit)</td><td><code>lmer()</code></td><td><code>logit(y / C) ~ (1|Batch) + x</code></td><td></td></tr>
<tr><td>LM (asin)</td><td><code>lmer()</code></td><td><code>asin(sqrt(y / C)) ~ (1|Batch) + x</code></td><td></td></tr>
<tr><td>Binomial</td><td><code>glmer()</code></td><td><code>cbind(y, C - y) ~ (1|Batch) + x</code></td><td><code>family=binomial(</code></td></tr>
</table>

Supplementary Table 2: Implementation of each method for mixed effects models

References

Aitchison, J. (1986). *The Statistical Analysis of Compositional Data.* Chapman and Hall, New York.

Bates, D., Mächler, M., Bolker, B., and Walker, S. (2015). Fitting Linear Mixed-Effects Models Using lme4. *Journal of Statistical Software* **67,** 1–48.

Büttner, M., Ostner, J., Müller, C. L., Theis, F. J., and Schubert, B. (2021). scCODA is a Bayesian model for compositional single-cell data analysis. *Nature Communications* **12,** 1–27.

Carroll, R. J. and Ruppert, D. (2017). *Transformation and Weighting in Regression.* Chapman and Hall/CRC, New York.

Egozcue, J. J., Graffelman, J., Ortego, M. I., and Pawlowsky-Glahn, V. (2020). Some thoughts on counts in sequencing studies. *NAR Genomics and Bioinformatics* **2,** lqaa094.

Egozcue, J. J., Pawlowsky-Glahn, V., Mateu-Figueras, G., and Barceló-Vidal, C. (2003). Isometric Logratio Transformations for Compositional Data Analysis. *Mathematical Geology* **35,** 279–300.

Graffelman, J., Egozcue, J. J., and M. I., O. (2015). On the asymptotic distribution of proportions of multinomial count data. In *Proceedings of the 6th International Workshop on Compositional Data Analysis.*

Hoffman, G. E., Lee, D., Bendl, J., Prashant, N., Hong, A., Casey, C., Alvia, M., Shao, Z., Argyriou, S., Therrien, K., Venkatesh, S., Voloudakis, G., Haroutunian, V., Fullard, J. F., and Roussos, P. (2024). Efficient differential expression analysis of large-scale single cell transcriptomics data using dreamlet.

Hoffman, G. E. and Roussos, P. (2021). Dream: Powerful differential expression analysis for repeated measures designs. *Bioinformatics* **37,** 192–201.

Hoffman, G. E. and Schadt, E. E. (2016). variancePartition: interpreting drivers of variation in complex gene expression studies. *BMC Bioinformatics* **17,** 483.

Lin, D. Y. and Sullivan, P. F. (2009). Meta-Analysis of Genome-wide Association Studies with Overlapping Subjects. *American Journal of Human Genetics* **85,** 862–872.

Pawlowsky-Glahn, V., Egozcue, J. J., and Tolosana-Delgado, R. (2015). *Modelling and Analysis of Compositional Data.* Wiley.

Phipson, B. and Smyth, G. K. (2010). Permutation P-values Should Never Be Zero: Calculating Exact P-values When Permutations Are Randomly Drawn. *Statistical Applications in Genetics and Molecular Biology* **9,**.
