# Supplementary material for: Fast, flexible analysis of differences in cellular composition with crumblr
Source: Nat Commun. 2026 Sep 23;17:9598. doi: 10.1038/s41467-026-75681-7 (PMC13601534; doi:10.1038/s41467-026-75681-7)
Supplement: Supplementary file 3 [file 41467_2026_75681_MOESM3_ESM.pdf]

nature portfolio

Peer Review File

Fast, flexible analysis of differences in cellular composition with crumblr

Corresponding Author: Dr Gabriel Hoffman

This file contains all reviewer reports in order by version, followed by all author rebuttals in order by version.

Version 0:

Reviewer comments:

Reviewer #1

(Remarks to the Author)
I have carefully evaluated the manuscript titled “Fast Flexible Analysis of Differences in Cellular Composition with Crumblr.” While the study introduces innovative computational approaches, several aspects require clarification and further development to meet the journal's standards. My detailed comments are provided below.

1. The manuscript introduces the `treeTest()` function within the multivariate testing framework; however, its function, theoretical underpinnings, and interpretation are not adequately described. A detailed explanation of its algorithm, purpose, computational steps, and output interpretation is necessary.

2. The current comparative analysis in multivariate testing is limited to Sidak, Fisher, and Lin-Sullivan methods. It is recommended that the authors compare their approach with other established methods such as the Cauchy Combination Test and the Harmonic Mean p-Value. For further guidance, the following references may be useful:
- Liu, Y., & Xie, J. (2020). “Cauchy combination test: a powerful test with analytic p-value calculation under arbitrary dependency structures.” Journal of the American Statistical Association, 115(529), 393–402.
- Wilson, D. J. (2019). “The harmonic mean p-value for combining dependent tests.” Proceedings of the National Academy of Sciences, 116(4), 1195–1200.
- Liu, Y., Chen, S., Li, Z., Morrison, A. C., Boerwinkle, E., & Lin, X. (2019). “ACAT: A fast and powerful p value combination method for rare-variant analysis in sequencing studies.” American Journal of Human Genetics, 104(3), 410–421.
- Liu, Y., & Xie, J. (2022). “Powerful and robust rare variant association testing by integrating multiple functional annotation data.” Biometrics, 78(1), 120–132.

3. The manuscript’s implementation of the Lin-Sullivan method differs from that originally described in the literature. The authors should detail the modifications, provide the corresponding derivations, and justify these deviations to clarify the methodological adjustments.

4. In the extended Lin-Sullivan approach, the empirical finite-sample null distribution is generated via Monte Carlo simulation, and p-values are calculated by estimating gamma distribution parameters. The following points need clarification:
- Why is the gamma distribution fitted instead of directly using the empirical null distribution for p-value calculation?
- How is the degrees of freedom (v) in the Wishart distribution defined in this context, and how does it differ from the residual degrees of freedom (d) in the weighted multivariate regression (formula 29)?

5. The manuscript claims that "The properties of the CLR transform make this formula simpler than for other transformations." Is this simplification attributed to asymptotic normality or other analytical properties? A more explicit explanation or reference to a derivation would support this assertion.

6. The derivation of the test statistic used for combining multiple cell-type components (Equation 29) requires further detail:
- How is this test statistic derived?
- What is the role of $\hat{\Omega}$, and how is it computed?
- Clarify the interpretation and estimation of the variance-covariance matrix of $\hat{B}_k$.
- Figure S11 is referenced in this context; please provide a clear description of how the “exact covariance” was derived and

interpret the findings shown in this figure.

7. In the weighted multivariate regression extension, the covariance of \(\beta\) is estimated using matrix \(C\) and weight residuals, with weights derived from \(\mathrm{var}[CLR_i(p)]\). It is not clear why the full covariance of \(CLR(p)\) is not incorporated. The authors should justify this choice and discuss the assumptions underlying the weighting strategy.

8. The formulation for the weighted linear mixed models appears to analogously follow from the weighted multivariate regression. However, more detailed derivations are needed, particularly:
- How are random effects parameters treated in the model?
- Are there additional adjustments or considerations for the random effects?

9. The results indicate that Crumblr does not clearly outperform negative-binomial methods—apart from computational speed—with the current implementation based on `glm.nb`. It is recommended that:
- The authors compare Crumblr with more widely used differential expression analysis methods that leverage the negative-binomial distribution (e.g., edgeR, DESeq2).
- Results should also be contrasted with non-parametric approaches like the Wilcoxon rank-sum test under simple conditions to contextualize performance.

10. The supplementary material provides only an expression for the CLR (Equation 3) and does not give an explicit analytical form for the ILR transformation, including the definition of the \(V\) matrix. Additionally:
- What is the derivative corresponding to Equation (7)?
- Why is the delta method applied using the ILR transform to derive the variance of CLR rather than directly applying the delta method to CLR?

11. The derivation of Equation (18) appears to be based on expressions for conditional and marginal variance. More explicit detail is needed regarding:
- The derivation and estimation of the parameter \(\tau\).
- Whether \(\tau = 1\) reduces the model to a multinomial special case. (noting that if $\tau=1$, then $n=1$ by the formula.)
- Clarification on whether \(x\) in Equation (16) corresponds to the count vector \(c\) used elsewhere.

12. Single-cell data often exhibit zero-inflation. The current manuscript does not address this issue. The authors should discuss potential adjustments or methodological extensions that account for zero-inflated distributions.

13. The manuscript presents four case studies; however, none provide sufficient details regarding the data processing workflow (e.g., cell annotation procedures) or in-depth interpretation of the biological results.
- A detailed description of the single-cell preprocessing steps is vital, as these can substantially influence the analysis outcomes.
- The inclusion of appropriate biological interpretations will help demonstrate the method's practical impact.

14. In Case Study 1, the authors report a decrease in the proportion of naive CD8\(^+\) T cells with age (Fig. 3D).
- Is this finding biologically significant?
- I recommend citing relevant literature to contextualize this observation.
Furthermore, the study's results regarding different cell types (e.g., central memory CD4\(^+\) T cells and regulatory T cells) should be discussed in terms of their biological relevance.

15. In Case Study 2, the observed decrease in Th1 or Th17 clusters among TB-infected individuals needs a discussion on its biological implications.

16. In Case Study 3, clarification is required on what "Disease Status" refers to (e.g., TNM stage) and the biological significance of pericyte upregulation in tumors.

While the Crumblr framework offers an innovative approach to analyzing differences in cellular composition, the manuscript requires substantial revisions to address the methodological and biological concerns detailed above. Enhancing the clarity of model derivations, providing comprehensive data processing details, and contextualizing the biological findings will considerably improve the robustness and overall impact of the study.

I look forward to your revised submission addressing these points.

Sincerely

(Remarks on code availability)
I evaluated the provided R package and associated code. Overall, the package installs correctly and the core code functions as expected, with no major issues during installation or execution. However, the additional analysis scripts rely primarily on the code itself for guidance. Due to the absence of certain datasets, I was unable to fully execute some of the supplementary analyses, which limits direct validation of all the results presented in the paper.

It would be helpful if the authors could provide a more comprehensive README file or additional documentation that includes detailed instructions on installing, running the application, and performing the associated analyses.

Reviewer #2

(Remarks to the Author)
I co-reviewed this manuscript with one of the reviewers who provided the listed reports. This is part of the Nature Communications initiative to facilitate training in peer review and to provide appropriate recognition for Early Career Researchers who co-review manuscripts.

(Remarks on code availability)

Reviewer #3

(Remarks to the Author)
Comments:

This manuscript introduces crumblr, a novel statistical framework for differential composition analysis of single-cell data, addressing the critical challenge of accurately identifying changes in cell type frequencies while accounting for variable measurement precision. By integrating centered log-ratio transformation with precision-weighted linear models and incorporating multivariate testing for hierarchical cell relationships, the authors propose a potentially powerful and flexible tool. The manuscript presents compelling simulation studies suggesting improved performance over existing methods and demonstrates practical utility through applications to diverse biological datasets. This work represents a valuable methodological advancement for the single-cell field.
While the manuscript is a strong contribution, the following points could be addressed to further enhance its clarity and impact:

Specific Points for Consideration:
1. Handling of Zero Counts: The manuscript should explicitly detail the strategy employed to handle zero counts for cell types within samples, which is a known issue for CLR-based methods. Was a pseudocount implemented? If so, justification for the chosen value and an analysis of sensitivity to this choice should be provided. While Figure 1B mentions the approximation works "when at least two counts are observed", the practical implementation for zero values needs clarification.
2. Overdispersion Parameter (τ): The rationale for setting the Dirichlet-multinomial overdispersion parameter τ to 1, based on the invariance of coefficient estimates to weight scaling, requires further justification. While coefficient estimates may be invariant, does fixing $\tau=1$ potentially impact the accuracy of the variance estimation itself (used for precision weights), particularly when true overdispersion might vary substantially between cell types or samples? Please elaborate on the implications of not estimating or incorporating τ.
3. Weight Regularization: The manuscript describes regularization of precision weights to mitigate the influence of samples with extremely high weights. Please provide further rationale for the specific regularization approach (limiting the max/quantile ratio) and the selection of default parameters (ratio=5, quantile=5%). A sensitivity analysis exploring the impact of these parameters on results would be informative.
4. Empirical Null for Multivariate Test: The extension of the Lin-Sullivan test using an empirical null distribution derived from Wishart sampling is an interesting approach to control false positives in small samples. Clarification is needed on how the degrees of freedom (v) for the Wishart sampling distribution are determined, particularly within the context of linear mixed models where degrees of freedom estimation can be complex.
5. Scalability and Performance: While the manuscript indicates favorable computational performance compared to scCODA, a more formal analysis of computational complexity would be beneficial. How does the runtime scale with the number of samples (n), cell types (D), covariates (c), and the complexity of random effects structures? Is the multivariate testing step computationally intensive compared to the univariate analyses?
6. Interpretation of Hierarchical Tests: When multivariate tests on internal nodes of the cell hierarchy yield significant results (e.g., Figures 4C, 5C), guidance on the biological interpretation is needed. Does significance indicate a coordinated change across child nodes, an average effect, or primarily reflect the strongest effect among children? How should researchers relate parent node significance back to individual cell types?
7. Simulation Comparisons: The comparison in simulations (Fig 2A) includes methods based on transformations (logit, arcsin-sqrt) suitable for proportions bounded [0,1], whereas CLR yields unbounded values. Please comment on the fairness of this comparison given the different data scales and underlying assumptions. Furthermore, discussion regarding how crumblr compares to other count-based compositional data analysis methods beyond those included (e.g., ALDEx2, ANCOM-BC) would strengthen the comparative analysis.
8. Modeling Non-Linear Effects: The aging dataset analysis (Fig 3D) raises an interesting point about linearity. While crumblr supported a linear trend where a standard model might suggest non-linearity, could the precision weighting potentially mask true biological non-linearities by down-weighting samples where frequencies are low (often at extremes of a biological range)? Please discuss how users might investigate or model potential non-linear associations within the crumblr framework.

(Remarks on code availability)
The provided vignette offers a clear, step-by-step demonstration of the crumblr package workflow using an example dataset, effectively illustrating its integration with established Bioconductor tools like variancePartition and limma/dream. The code appears well-structured and covers key functionalities including data transformation, variance decomposition, PCA, differential testing, and the novel multivariate tree analysis. To further enhance usability, consider adding more detailed explanations within the vignette regarding the interpretation of intermediate objects (like cobj) and the rationale for specific functions (e.g., standardize()). Additionally, explicitly showing or referencing the method used to generate the input hierarchy (hcl) for treeTest, and providing brief guidance on potential scalability considerations or parameter tuning for larger, more complex datasets would be beneficial for prospective users.

Version 1:

Reviewer comments:

Reviewer #1

(Remarks to the Author)
The authors have addressed many of the earlier concerns, and the paper is substantially improved in clarity, scope, and empirical evaluation. However, I have identified several remaining issues that require attention.

1. There are inconsistencies in the mathematical notation throughout the manuscript that may cause confusion for readers. Estimated statistics should be marked with hat notation (^), while true parameters should not. Specific examples include:

In Formula 56: Σ is described as a "known variance-covariance matrix," but the text subsequently indicates it is estimated through formulas 42-52. If estimated, it should be denoted as Σ.
In Formula 14: The term $\text{clr}(p)$ should be $\text{clr}(\hat{p})$ since p represents an estimate.
Similarly, in Formulas 15, 20, 21, and 25: $\text{clr}(p)$ should be $\text{clr}(\hat{p})$ to reflect that these are based on estimated proportions.
I recommend a comprehensive review of all mathematical expressions to ensure consistent notation distinguishing between parameters and their estimates.

2. Regarding the Dirichlet-multinomial (DMN) dispersion parameter τ and the weighting scheme, several points require clarification:

Based on Formula 33, weights $w_{i,k}$ are proportional to the inverse variance $v_{i,k}$. Is $v_{i,k}$ equivalent to $\text{var}[\text{clr}(\hat{p}_i)]$ from Formula 31?
The manuscript states that relative weights are invariant to the value of τ. Could you explain why this is the case? Are the weights scaled and standardized so that their sum equals 1?
A more detailed explanation of how the weights are derived and normalized would be beneficial.

3. Following up on my previous Comment 7, I note that $\text{var}[\text{clr}(\hat{p}_i)]$ from Formula 31 is used as weights for weighted multiple regressions. However, these appear to be only the diagonal entries of the complete variance-covariance matrix $\text{var}[\text{clr}(\hat{p})]$. The off-diagonal entries, representing covariances such as $\text{cov}(\text{clr}(\hat{p}_i), \text{clr}(\hat{p}_j))$, do not appear to be incorporated into subsequent regressions.

If this interpretation is correct, it suggests that some correlation information inherent in the clr transformation is being discarded. Please clarify:

Whether only diagonal elements are used as weights
The rationale for not incorporating the full covariance structure
Any potential implications of ignoring the off-diagonal covariance terms
Whether this simplification affects the validity or efficiency of the statistical inference

(Remarks on code availability)
The GitHub link provided in the response to Comment 4 appears to be broken (https://github.com/GabrielHoffman/crumblr_analysis/blob/main/simulations/remaCor_gamma.Rmd). This link returns an error and should be verified and corrected to ensure reproducibility of the results.

While the analysis code on GitHub is detailed and logically organized, I was unable to run it successfully in practice due to data-loading errors. Within the limited time available, I was not able to diagnose and resolve the issue.

Reviewer #3

(Remarks to the Author)

Thanks for the very thorough and thoughtful response to my previous comments. I've read through the revised manuscript, and I'm really impressed by the amount of work you put into it. The new simulations, the added details in the methods, and the improved biological context have made the paper much clearer and more convincing. All of my previous concerns have been fully addressed. All in all, this is an excellent piece of work. Congratulations!

(Remarks on code availability)
Yes, I've reviewed the code and the associated documentation. The R package installs without any issues, and the core functions work as described. I was particularly pleased to see the significant improvements made to the documentation following the last review. The new README files for each analysis on the GitHub repository are very helpful, and the updated vignette now provides much clearer instructions on how to run the analyses, interpret the results, and handle things like the hierarchical clustering input for treeTest . These changes have successfully addressed the previous concerns about reproducibility . I'm now confident that the code is a well-documented and valuable resource for the community.

Version 2:

Reviewer comments:

Reviewer #1

(Remarks to the Author)
I appreciate the authors'clarification in their response that only the diagonal elements of the variance–covariance matrix (i.e., var[clr(p_i)]) were used as weights, without incorporating the off-diagonal covariance terms. However, I must respectfully insist that this methodological choice requires explicit documentation in the manuscript itself, not merely in the response letter.This is essential for methodological transparency and reproducibility.

The authors should provide a brief rationale for this simplification. While I understand the authors consider full covariance incorporation to be "out of scope," readers need to understand whether this simplification is made for computational convenience or conceptual reasons.

The authors correctly note in their response that the generalized least squares (GLS) estimator incorporating the full variance-covariance matrix would be the Best Linear Unbiased Estimator (BLUE), and that weighted least squares (WLS) using only diagonal elements represents a special case of GLS. This point actually strengthens my concern: since the theoretical framework already accommodates GLS, and the full var[clr(p)] matrix is available from Formula 31, using the complete covariance structure would be straightforward within the existing framework and would yield more efficient estimates. The fact that the full GLS approach fits naturally into the authors' existing theoretical framework makes the omission of covariance terms more puzzling.

I recommend adding a brief paragraph in the Methods section and/or a note in the Discussion/Limitations section addressing these points. This addition would significantly enhance the methodological rigor and transparency of the work without requiring substantial revisions to the analysis itself.

(Remarks on code availability)
I have reviewed the section related to the code. I appreciate that authors have addressed the issues raised in the previous round of review. The overall structure of the code is coherent and adheres to established coding standards.However, due to time constraints, I was unable to run tests on all portions of the code.

Open Access This Peer Review File is licensed under a Creative Commons Attribution 4.0 International License, which permits use, sharing, adaptation, distribution and reproduction in any medium or format, as long as you give appropriate credit to the original author(s) and the source, provide a link to the Creative Commons license, and indicate if changes were made.
In cases where reviewers are anonymous, credit should be given to 'Anonymous Referee' and the source.
The images or other third party material in this Peer Review File are included in the article's Creative Commons license, unless indicated otherwise in a credit line to the material. If material is not included in the article's Creative Commons license and your intended use is not permitted by statutory regulation or exceeds the permitted use, you will need to obtain permission directly from the copyright holder.
To view a copy of this license, visit https://creativecommons.org/licenses/by/4.0/

Response to reviewers.

We thank the reviewers for providing valuable feedback on our manuscript. Our responses are shown below in black, indented text. Changes to the manuscript are shown in red text.

Reviewer #1 (Remarks to the Author):

I have carefully evaluated the manuscript titled "Fast Flexible Analysis of Differences in Cellular Composition with Crumblr." While the study introduces innovative computational approaches, several aspects require clarification and further development to meet the journal's standards. My detailed comments are provided below.

1. The manuscript introduces the `treeTest()` function within the multivariate testing framework; however, its function, theoretical underpinnings, and interpretation are not adequately described. A detailed explanation of its algorithm, purpose, computational steps, and output interpretation is necessary.

> We thank the Reviewer for this feedback. We have added a new section to the Methods:
>
> **Multivariate hierarchical testing with `treeTest()`**
>
> In the field of single-cell transcriptomics, the number of cell types analysed varies widely across studies. For example, Mathys, et al.[1] perform analysis on 12 major cell types and 54 higher resolution clusters from human prefrontal cortex to identify gene expression changes in Alzheimer's disease, while the BRAIN Initiative Cell Census Network identified 31 cell superclusters, 461 clusters, and 3313 subclusters across multiple brain regions[2]. In the brain, there is vast diversity in neuronal subtypes, but much less diversity in other cell types[2]. In practice, the number of cell type clusters used is determined by the tissue, the number of cells observed, and the biological question.
>
> Yet in analyses of differential cell type composition, the number of cell clusters used can affect the analysis due to the tradeoff between resolution and statistical power. While increasing the number of cell clusters enhances cell type resolution and the biological specificity of each cluster, the frequency of each higher-resolution cluster decreases. All analysis methods we considered suffer from a loss in power as the cell type frequency decreases. This fundamental tradeoff leaves the analyst to select the appropriate number of cell types to address their biological question.

Instead of selecting this number at the beginning of a project, the `treeTest()` analysis in the `crumblr` framework performs tests of differential abundance at multiple levels of cell type resolution. Given a hierarchical clustering of cell types, the method performs a multivariate hypothesis test on each internal node in the hierarchy. Given D total cell clusters at the leaves of this hierarchy, consider an internal node ℓ with d_ℓ descendant leaves. Letting $\hat{\vec{\beta}}_\ell$ be the vector of estimated coefficients from descendant leaves of node ℓ, we want to test the null hypothesis that the mean effect size is zero. Since high-resolution cell clusters often have frequencies that covary, modeling covariance in the estimated coefficients increases power to reject the null hypothesis while controlling the false positive rate. This test of the mean effect of descendant leaf nodes is repeated for each internal node using the empirical Lin-Sullivan test.

By testing at internal nodes, this approach can accumulate power across multiple leaves where testing at a single leaf is underpowered. Rejecting the null hypothesis at an internal node where few or no leaves show a significant signal can indicate that high-resolution clustering caused loss of power at the leaves, which is then 'rescued' by the multilevel analysis. However, note that a strong signal in one leaf can drive the result at an internal node where all other leaves have small effects, as we see in **Fig. 3B**. We also note that the results depend on the hierarchical clustering used.

2. The current comparative analysis in multivariate testing is limited to Sidak, Fisher, and Lin-Sullivan methods. It is recommended that the authors compare their approach with other established methods such as the Cauchy Combination Test and the Harmonic Mean p-Value. For further guidance, the following references may be useful:
- Liu, Y., & Xie, J. (2020). "Cauchy combination test: a powerful test with analytic p-value calculation under arbitrary dependency structures." Journal of the American Statistical Association, 115(529), 393–402.
- Wilson, D. J. (2019). "The harmonic mean p-value for combining dependent tests." Proceedings of the National Academy of Sciences, 116(4), 1195–1200.
- Liu, Y., Chen, S., Li, Z., Morrison, A. C., Boerwinkle, E., & Lin, X. (2019). "ACAT: A fast and powerful p value combination method for rare-variant analysis in sequencing studies." American Journal of Human Genetics, 104(3), 410–421.
- Liu, Y., & Xie, J. (2022). "Powerful and robust rare variant association testing by integrating multiple functional annotation data." Biometrics, 78(1), 120–132.

We thank the reviewer for this suggestion. We have added the aggregated Cauchy association test (ACAT) and the harmonic mean p-value to our simulation study. These methods are designed to be robust to deviations from

independence, and as expected, they control the false positive rate in our simulations of correlation responses. However, they appear to be less powerful than the Lin-Sullivan methods that explicitly model the known dependency structure between the tests.

We have updated Figure 2 and the text in the Results:

We also consider Fisher's method that combines p-values from all tests and compares a test statistic to a chi-squared null distribution, the aggregated Cauchy association test that transforms the p-values and compares a test statistic to a Cauchy distribution [3], and the harmonic mean of the p-values [4]. Yet these methods don't consider the effect size or model the known dependency structure between tests, resulting in lower power.

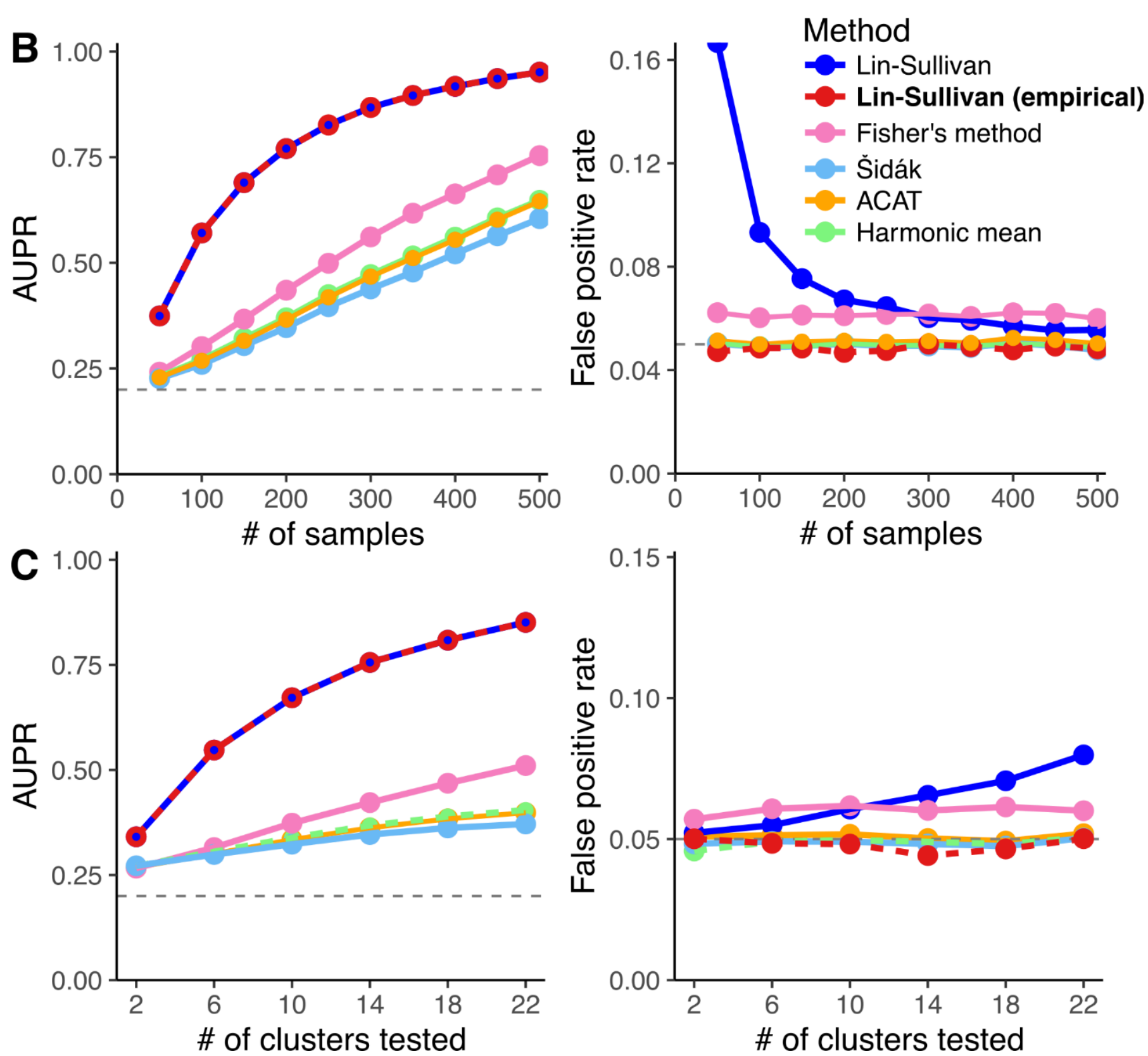

Figure 2: Performance on simulated cellar composition data.
B) Performance of multivariate testing in simulated data for increasing sample sizes. AUPR (left) and FPR (right) of 6 methods for multivariate testing. **C)** Performance of multivariate testing in simulated data for increasing number of cell clusters tested. AUPR (left) and FPR (right).

3. The manuscript's implementation of the Lin-Sullivan method differs from that originally described in the literature. The authors should detail the modifications, provide the corresponding derivations, and justify these deviations to clarify the methodological adjustments.

We thank the Reviewer for this feedback. We have added a new section to the Supplementary Methods with the full derivation of the Lin-Sullivan method and our novel contribution.

1.3.3 Multivariate hypothesis testing

Given a vector of coefficient values with one estimated for each response, we propose a hypothesis test comparing the mean coefficient value to a null value of zero. Let $\hat{\boldsymbol{\gamma}} = (\hat{\gamma}_1, ..., \hat{\gamma}_K)$ be the vector of estimated coefficients from across K responses and $\boldsymbol{\Omega}$ be the *known* covariance matrix, so that

$$\hat{\boldsymbol{\gamma}} \sim \mathcal{N}\left(\mu_\gamma, \boldsymbol{\Omega}\right). \tag{53}$$

Following Lin and Sullivan (2009), we test the null hypothesis that $\mu_\gamma = 0$ using maximum likelihood to obtain

$$\hat{\mu}_\gamma = \frac{\mathbf{1}^T \boldsymbol{\Omega}^{-1} \hat{\boldsymbol{\gamma}}}{\mathbf{1}^T \boldsymbol{\Omega}^{-1} \mathbf{1}} \tag{54}$$

$$\text{var}(\hat{\mu}_\gamma) = \frac{1}{\mathbf{1}^T \boldsymbol{\Omega}^{-1} \mathbf{1}}, \tag{55}$$

with the corresponding z-score

$$\frac{\hat{\mu}_\gamma}{\sqrt{\text{var}(\hat{\mu}_\gamma)}} = \frac{\mathbf{1}^T \boldsymbol{\Omega}^{-1} \hat{\boldsymbol{\gamma}}}{\sqrt{\mathbf{1}^T \boldsymbol{\Omega}^{-1} \mathbf{1}}}. \tag{56}$$

This method combines effect size estimates across multiple responses while modeling their dependency structure using the covariance matrix $\boldsymbol{\Omega}$. When the covariance matrix $\boldsymbol{\Omega}$ is known, rather than being estimated from the data, the test statistic is normally distributed. We apply this method to multivariate regression from above by setting $\hat{\boldsymbol{\gamma}}$ to the vector of estimated coefficients across responses from linear or linear mixed model, and $\boldsymbol{\Omega}$ to the covariance matrices from Equations (42) or (52).

1.3.4 An adaptation for unknown variance

Consider the simple linear model with n samples and only a mean term,

$$y = \mu + \varepsilon \tag{57}$$

$$\varepsilon \sim \mathcal{N}(0, \sigma^2), \tag{58}$$

where the estimated residual variance is

$$s^2 = \sum_i (y_i - \hat{\mu})^2 / \nu, \tag{59}$$

with $\nu = n - 1$ residual degrees of freedom. Standard theory shows that

$$\frac{\nu s^2}{\sigma^2} \sim \chi^2_\nu, \tag{60}$$

and the z-score has a Student-t distribution

$$\frac{\hat{\mu}}{s/\sqrt{n}} \sim t_\nu. \tag{61}$$

This statistic has a Student-t distribution since the residual variance is unknown and is estimated from the data.

Extending this result to the multivariate case, the residual sum of squares matrix, $\mathbf{R}^T\mathbf{R}$, is drawn from a Wishart distribution according to

$$\mathbf{R}^T\mathbf{R} \sim Wishart(\mathbf{\Sigma}, \nu), \tag{62}$$

where $\mathbf{\Sigma}$ is the true error covariance matrix, and ν is the residual degrees of freedom.

We can now approximate the null distribution by sampling covariance matrices from a Wishart distribution centered at the observed residual sum of squares matrix according to

$$\hat{\mathbf{\Omega}} \sim Wishart(\mathbf{R}^T\mathbf{R}, \nu) \tag{63}$$

$$\hat{\boldsymbol{\gamma}} \sim \mathcal{N}(0, \hat{\mathbf{\Omega}}). \tag{64}$$

We use these sampled values to evaluate the test statistic in Equation (56) using Algorithm 1.

Algorithm 1 Monte Carlo sampling of the Lin-Sullivan statistic under the null

1) Sample residual sum of squares matrix

   $\mathbf{\Omega}_1 \sim Wishart(\mathbf{R}^T\mathbf{R}, \nu)$

2) Sample vector of regression coefficients using an independent

   sample of the residual sum of squares matrix

   $\mathbf{\Omega}_2 \sim Wishart(\mathbf{R}^T\mathbf{R}, \nu)$

   $\boldsymbol{\gamma} \sim \mathcal{N}(0, \mathbf{\Omega}_2)$

3) Compute z-statistic

$$z = \frac{\mathbf{1}^T\mathbf{\Omega}_2^{-1}\hat{\boldsymbol{\gamma}}}{\sqrt{\mathbf{1}^T\mathbf{\Omega}_1^{-1}\mathbf{1}}} \tag{65}$$

4. In the extended Lin-Sullivan approach, the empirical finite-sample null distribution is generated via Monte Carlo simulation, and p-values are calculated by estimating gamma distribution parameters. The following points need clarification:
- Why is the gamma distribution fitted instead of directly using the empirical null distribution for p-value calculation?
- How is the degrees of freedom (v) in the Wishart distribution defined in this context, and how does it differ from the residual degrees of freedom (d) in the weighted multivariate regression (formula 29)?

We have changed the notation throughout so the residual degrees of freedom is denoted by ν.

We used a gamma approximation here in order to reduce the computational time needed to compute p-values. We have added the following to the Supplementary Methods.

Let z_i be the ith of a total of κ values of the test statistic drawn from the null distribution, and z_{obs} be the observed values of the test statistic from the data. The p-value for a two-sided test can be computed by comparing z_{obs}^2 to z_i^2 according to

$$\frac{1 + \sum_{i=1}^{\kappa} z_{obs}^2 > z_i^2}{\kappa + 1}, \tag{53}$$

adding 1 to the numerator and denominator to avoid p-values of zero (Phipson and Smyth, 2010).

However, this raises two issues: 1) the smallest empirical p-value that can be computed is $1/(\kappa+1)$, and 2) the sampling error of the p-value can be substantial for small p-values. This means that κ must be very large in order to produce small precise p-values. This can be very computationally expensive. In order to reduce the number of Monte Carlo samples needed, a gamma distribution is then fit to these draws from the null distribution, and an empirical p-value is computed from the cumulative distribution function of this gamma.

We have checked this gamma approximation empirically. Plotting the -log10 p-value from the empirical and gamma methods, we see that they are very similar for large p-values. For smaller p-values with fixed κ, the boundary of $1/(1+\kappa)$ becomes an issue.

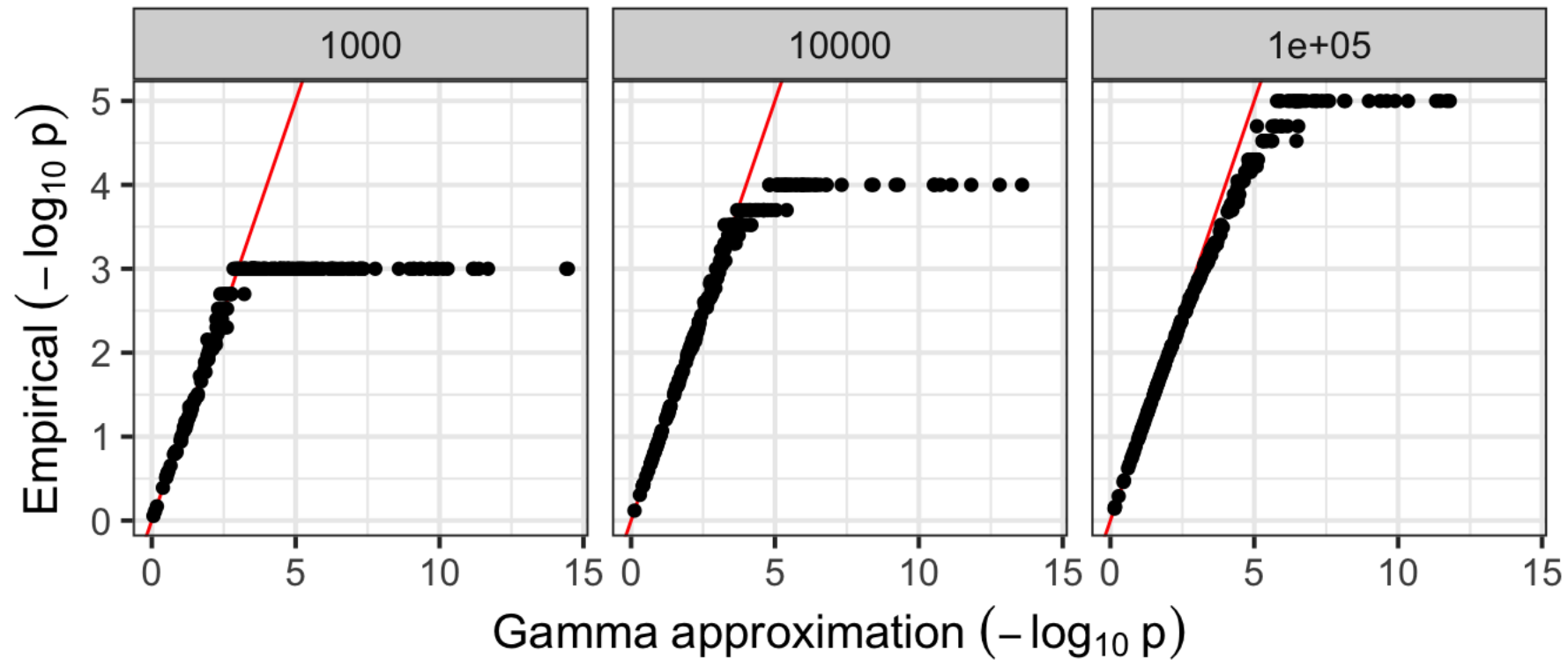

Comparison of p-values from empirical distribution and gamma approximation. Simulations were performed for 100 samples, 6 responses. Results are plotted for 1k (left), 10k (center), and 100k (right) Monte Carlo samples. Line with slope of 1 is indicated in red. See analysis code here.

We acknowledge that this assumes that the gamma distribution is a good approximation of the underlying empirical distribution. Across a range of simulations varying sample size, number of responses and error distribution, we find that a gamma is a good approximation.

In remaCor v0.0.20, we have added the flag `useGamma` to the function `LS.empirical()`, so the user can disable the gamma approximation.

5. The manuscript claims that "The properties of the CLR transform make this formula simpler than for other transformations." Is this simplification attributed to asymptotic normality or other analytical properties? A more explicit explanation or reference to a derivation would support this assertion.

We acknowledge this statement was imprecise, and we have removed it.

6. The derivation of the test statistic used for combining multiple cell-type components (Equation 29) requires further detail:
- How is this test statistic derived?
- What is the role of $\hat{\Omega}$, and how is it computed?
- Clarify the interpretation and estimation of the variance-covariance matrix of $\hat{B}_k$.
- Figure S11 is referenced in this context; please provide a clear description of how the "exact covariance" was derived and interpret the findings shown in this figure.

We thank the Reviewer for this feedback. Please see the derivation of the Lin-Sullian statistic above.

We have clarified the text in the Methods:

Letting $\hat{B}_k$ be the vector of coefficient estimates for variable k across m responses, $\hat{\Omega}$ be the estimated covariance matrix of the coefficients from the multivariate regression, and 1 be a vector of 1's, the test statistic

$$\frac{\mathbf{1}^T\hat{\Omega}^{-1}\hat{B}_k}{\mathbf{1}^T\hat{\Omega}^{-1}\mathbf{1}}$$

is asymptotically normally distributed under the null.

In the Supplementary Methods we have added the following text:

This method combines effect size estimates across multiple responses while modeling their dependency structure using the covariance matrix $\mathbf{\Omega}$. When the covariance matrix $\mathbf{\Omega}$ is known, rather than being estimated from the data, the test statistic is normally distributed. We apply this method to multivariate regression from above by setting $\hat{\boldsymbol{\gamma}}$ to the vector of estimated coefficients across responses from linear or linear mixed model, and $\mathbf{\Omega}$ to the covariance matrices from Equations (42) or (52).

We have improved the text describing how we assessed the approximate covariance in the Supplementary Methods.

With linear regression and weights varying across samples and responses, we can compare the exact covariance from Equation (38) with our approximation in Equation (52). In the case of general weights, simulations indicate that this approximation produces accurate estimates of the covariance in coefficient estimates across responses (**Figure S11**). This suggests that the approximation is also good in the case of linear mixed model, even though we can't compare against the exact covariance in that case.

7. In the weighted multivariate regression extension, the covariance of β is estimated using matrix C and weight residuals, with weights derived from $\text{var}[CLR_i(p)]$. It is not clear why the full covariance of $CLR(p)$ is not incorporated. The authors should justify this choice and discuss the assumptions underlying the weighting strategy.

This is not a modelling choice, it follows from first principles. Unrelated to cell type composition, consider a dataset with D response vectors and a multivariate regression fit on $k < D$ responses. The coefficient estimates and their covariances depend on the k responses fit in the model. But they are unrelated to the $D - k$ responses omitted from the model. In the case of compositional analysis with `crumblr`, the sampling variances of the CLR transformed proportions involve all cell clusters according to

$$\widehat{\text{var}[\text{clr}(\hat{p}_i)]} = \frac{\hat{\tau}}{n}\left[\frac{1}{\hat{p}_i} - \frac{2}{D\hat{p}_i} + \frac{1}{D^2}\sum_{j=1}^{D}\frac{1}{\hat{p}_j}\right].$$

But after using these values to compute the weights for the the regression model of each response, and fitting multivariate regression involving k responses, the estimating coefficients and covariance is unrelated to the $D - k$ responses omitted from the model.

8. The formulation for the weighted linear mixed models appears to analogously follow from the weighted multivariate regression. However, more detailed derivations are needed, particularly:
- How are random effects parameters treated in the model?
- Are there additional adjustments or considerations for the random effects?

We thank the Reviewer for this question. Random effects are estimated using REML in the linear mixed model as in the standard `lme4` R package, but hypothesis testing is only performed on fixed effects. Given the model fit from `lme4`, the covariance estimates from each response are combined across responses for multivariate testing.

We have added more detail to the <u>Supplementary Methods</u>:

Residual degrees of freedom

In the case of a linear model, the residual degrees of freedom, ν, is computed from the sample size and the number of coefficients estimated. In the case of the a linear mixed model, the form of the residual degrees of freedom is more complicated. We use an approximation we developed previously (Hoffman et al., 2024). See Supplementary Methods Section 2.3.1 of that work for the full derivation. It is implemented in `variancePartition::rdf.merMod()` and computes the residuals degrees of freedom from a linear mixed model fit with `lme4::lmer()`.

1.3.2 Weighted linear mixed model

For the linear mixed model, closed form estimates don't exist and parameters are estimated using numerical optimization in `lme4` (Bates et al., 2015). Here we build up to the covariance estimates starting at the unweighted linear model. Let $\hat{\mathbf{\Sigma}}_i$ be the estimated covariance matrix between coefficients for response i. In the case of standard, unweighted linear regression, $\hat{\mathbf{\Sigma}}_i$ has the standard form

$$\hat{\mathbf{\Sigma}}_i = \left(\mathbf{X}^T\mathbf{X}\right)^{-1}\hat{\sigma}_e^2. \tag{43}$$

Incorporating weights and using the notation as above, the estimator becomes

$$\hat{\mathbf{\Sigma}}_i = \left(\tilde{\mathbf{X}}_i^T\tilde{\mathbf{X}}_i\right)^{-1}\tilde{r}_i^T\tilde{r}_i/\nu, \tag{44}$$

and equals the previous form when all weights are 1.

In the unweighted linear mixed model,

$$\begin{aligned} y &= \mathbf{X}\beta + \mathbf{Z}u + \varepsilon && (45)\\ u &= \mathcal{N}(0, \mathbf{G}) && (46)\\ \varepsilon &= \mathcal{N}(0, \mathbf{I}\sigma^2), && (47) \end{aligned}$$

with the variance parameters in $\mathbf{G}$, the covariance between estimated coefficient is

$$\begin{aligned} \hat{\mathbf{\Sigma}}_i &= \left(\mathbf{X}^T\mathbf{V}_i^{-1}\mathbf{X}\right)^{-1} && (48)\\ \mathbf{V}_i &= \mathbf{Z}\mathbf{G}_i\mathbf{Z}^T + \mathbf{I}\hat{\sigma}_i^2. && (49) \end{aligned}$$

Extending this to the case of the weighted linear mixed model gives

$$\begin{aligned} \hat{\mathbf{\Sigma}}_i &= \left(\tilde{\mathbf{X}}_i^T\mathbf{V}_i^{-1}\tilde{\mathbf{X}}_i\right)^{-1} && (50)\\ \mathbf{V}_i &= \tilde{\mathbf{Z}}_i\mathbf{G}_i\tilde{\mathbf{Z}}_i^T + \mathbf{I}\hat{\sigma}_i^2. && (51) \end{aligned}$$

In Equation (42), we stated the covariance in terms of $\tilde{\mathbf{X}}_i$ and $\tilde{\mathbf{X}}_j$ by implicitly using the result of Equation (44). But in the case of the weighted linear mixed model, $\hat{\mathbf{\Sigma}}_i$ is more complicated.

Using the form of Equation (38), the covariance in coefficient estimates between responses i and j can be stated in terms of the covariance for each response and the correlation between residuals when all weights are 1. When weights vary, the covariance between coefficients estimates for fixed effects in the linear mixed model is approximated as

$$\text{cov}\left(\hat{\beta}_i, \hat{\beta}_j\right) = \text{cor}(\tilde{r}_i, \tilde{r}_j)\hat{\mathbf{\Sigma}}_i\left(\hat{\mathbf{\Sigma}}_i^{-\frac{1}{2}}\hat{\mathbf{\Sigma}}_j^{-\frac{1}{2}}\right)\hat{\mathbf{\Sigma}}_j \tag{52}$$

When $i = j$, this formula reduces to the $\hat{\mathbf{\Sigma}}_i$. In the case of linear regression, when the weights vary across samples but are shared across responses, this method is equivalent to Equation (38).

9. The results indicate that Crumblr does not clearly outperform negative-binomial methods—apart from computational speed—with the current implementation based on `glm.nb`. It is recommended that:
- The authors compare Crumblr with more widely used differential expression analysis methods that leverage the negative-binomial distribution (e.g., edgeR, DESeq2).
- Results should also be contrasted with non-parametric approaches like the Wilcoxon rank-sum test under simple conditions to contextualize performance.

We thank the reviewer for this valuable feedback. We have added edgeR, DESeq2 and Wilcoxon tests to the simulations. Our crumblr method matches the power of negative binomial methods for univariate tests with fixed. But our contribution is that crumblr can incorporate random effects and multivariate testing while being scalable to large datasets.

Based on this feedback and the comments from the other Reviewer, we have updated the simulations in **Fig 2A** and **Fig S2-8**, and the text in the Results:

Compared to 12 other methods for univariate testing of changes in cellular composition, `crumblr` matches the statistical performance of negative binomial methods having highest statistical power. Yet of these, only `crumblr` controls the false positive rate across conditions under a range of conditions, has the flexibility to incorporate random effects and enables multivariate testing while being scalable to large datasets (**Fig S1**).

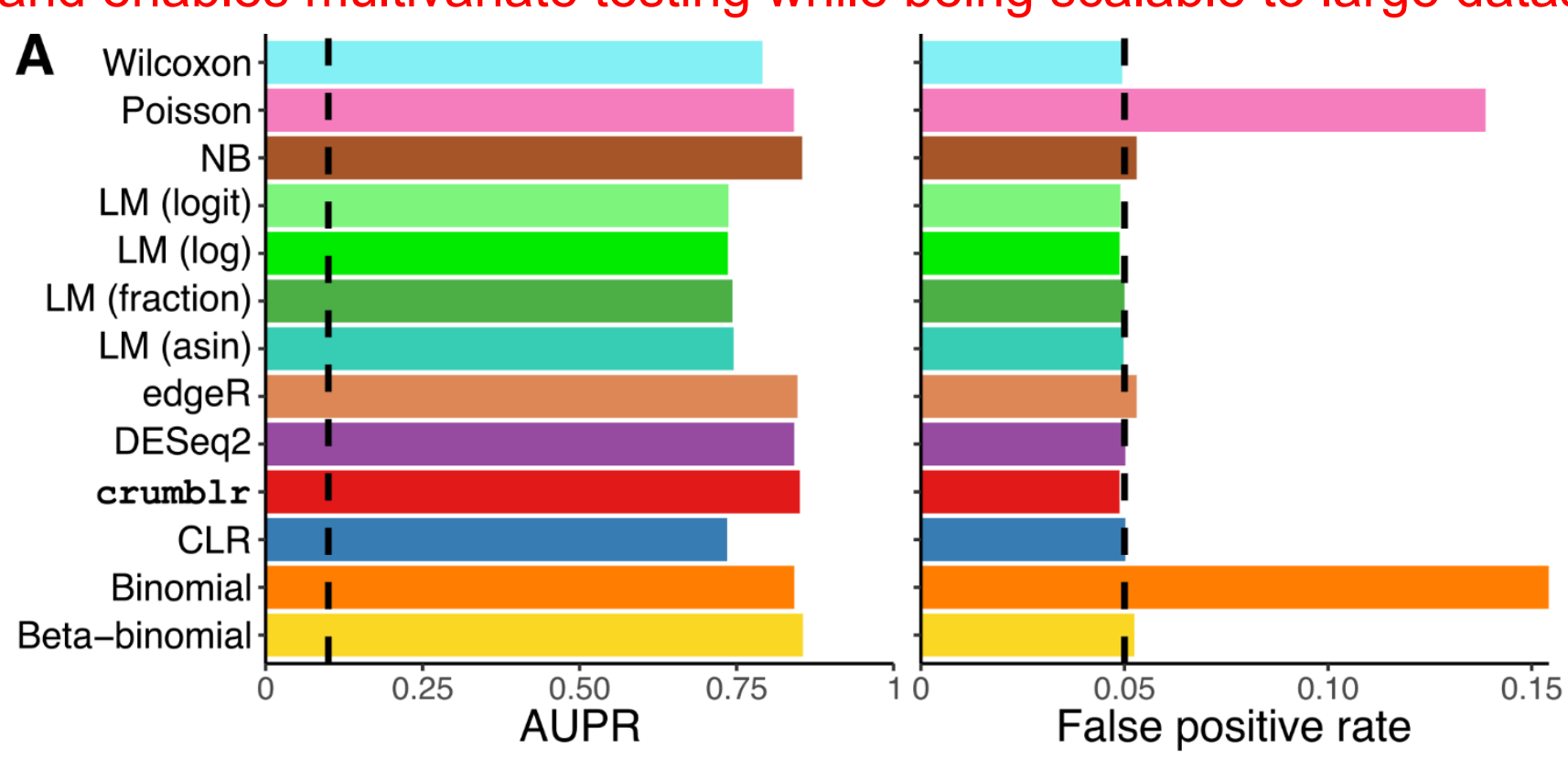

Figure 2: Performance on simulated cellar composition data. A) Performance of univariate tests across 10k simulated datasets under baseline parameters. For linear models indicated with `LM`, `fraction` indicates using the cell fraction, f, as the response, `log` indicates $log(f)$, `logit` indicates $logit(f) = log(f) - log(1-f)$, and `asin` indicates $arcsin(\sqrt{f})$. The area under the precision-recall curve (AUPR) (left) and false positive rate (FPR) at a 5% cutoff computed under the null model (right). For AUPR, the dashed line indicates the performance of the random method. For FPR, the dashed line indicates the target for a properly calibrated method.

10. The supplementary material provides only an expression for the CLR (Equation 3) and does not give an explicit analytical form for the ILR transformation, including the definition of the V matrix. Additionally:
- What is the derivative corresponding to Equation (7)?
- Why is the delta method applied using the ILR transform to derive the variance of CLR rather than directly applying the delta method to CLR?

We thank the Reviewer for this feedback. We have substantially expanded the Supplementary Methods to address these questions:

1.1 Isometric log ratio

Since proportions are constrained to sum to 1, proportions divided into D variables actually span a $D-1$ dimensional space. This is also true for clr transformed proportions. This degenerate set of variables can be problematic in statistical modeling since the covariance matrix across clr transformed proportions is not invertible. To address this Egozcue et al. (2003) proposed the isometric log ratio (ilr) to map the clr transformed variables into $D-1$ variables. Importantly, the mapping between clr and ilr coordinates is a linear operation using an orthonormal transform.

Egozcue et al. (2003) construct the orthonormal matrix V and define the ilr coordinates as

$$\text{ilr}(\mathbf{p}) = \mathbf{V}^T \text{clr}(\mathbf{p}), \tag{2}$$

so that distances in clr space are preserved in the ilr space. By construction $\mathbf{V}$ is a $D \times D-1$ matrix thats satisfies properties

$$\mathbf{V}^T\mathbf{V} = \mathbf{I}_{D-1} \tag{3}$$

$$\mathbf{V}\mathbf{V}^T = \mathbf{I}_D - \frac{1}{D}\mathbf{1}\mathbf{1}^T \tag{4}$$

$$\mathbf{V}^+ = \mathbf{V}^T \tag{5}$$

where $\mathbf{I}_D$ is the $D \times D$ identify matrix, $\mathbf{1}$ is a vector of D 1's, and $\mathbf{V}^+$ indicates the Moore–Penrose inverse. It follows that the inverse projection from ilr coordinates back to clr coordinates is

$$\text{clr}(\mathbf{p}) = \mathbf{V}\,\text{ilr}(\mathbf{p}) \tag{6}$$

Populating $\mathbf{V}$ with numeric values that satisfy these properties is quite complicated and is described in detail by Egozcue et al. (2003). But understanding these details doesn't add to the intuition about the current work. Importantly, we note that our work uses $\mathbf{V}$ in derivations, but, due to its special properties, all instances of the matrix cancel out so it never needs to be instantiated.

1.2.1 Asymptotic normal approximation of multinomial counts

Let $\mathbf{p}$ be the true vector of proportions for D components with p_i being the true proportion for the ith component. Let $\mathbf{c}$ be the vector of observed counts drawn from a multinomial distribution with n total counts. The expected number of counts is $E[\mathbf{c}] = n\mathbf{p}$, the maximum likelihood estimate of the proportions is $\hat{\mathbf{p}} = \mathbf{c}/n$, and the variance of the counts is $n\left[\text{diag}(\mathbf{p}) - \mathbf{p}\mathbf{p}^T\right]$. Based asymptotic properties of multinomial distribution, for large n the distribution of the estimated proportions converges to a multivariate normal distribution

$$\hat{\mathbf{p}} \xrightarrow{d} \mathcal{N}\left(\mathbf{p}, \frac{1}{n}\left[\text{diag}(\mathbf{p}) - \mathbf{p}\mathbf{p}^T\right]\right). \tag{7}$$

Following Egozcue et al. (2020) and Graffelman et al. (2015), we use the delta method to obtain the asymptotic normal distribution of the ilr transformed estimated proportions. Consider a transformation $g(.)$ applied to any vector of random variables $\mathbf{x}$ with covariance $\mathbf{\Sigma_x}$, then the asymptotic variance of the transformed random variable is

$$\left(\frac{\partial g(\mathbf{x})}{\partial \mathbf{x}}\right) \mathbf{\Sigma}_x \left(\frac{\partial g(\mathbf{x})}{\partial \mathbf{x}}\right)^T. \tag{8}$$

Applying this to the ilr transform gives

$$\frac{\partial\, \text{ilr}(\mathbf{p})}{\partial \mathbf{p}} = \frac{\partial \mathbf{V}^T \log(\mathbf{p})}{\partial \mathbf{p}} \tag{9}$$

$$= \mathbf{V}^T \text{diag}(1/\mathbf{p}) \tag{10}$$

so that

$$\text{var}[\text{ilr}(\mathbf{p})] = n^{-1}\left[\mathbf{V}^T\text{diag}(1/\mathbf{p})\right]\left[\text{diag}(\mathbf{p}) - \mathbf{p}\mathbf{p}^T\right]\left[\text{diag}(1/\mathbf{p})\mathbf{V}\right] \tag{11}$$

$$= n^{-1}\mathbf{V}^T\text{diag}(1/\mathbf{p})\mathbf{V} - \mathbf{V}^T\mathbf{1}\mathbf{1}^T\mathbf{V} \tag{12}$$

$$= n^{-1}\mathbf{V}^T\text{diag}(1/\mathbf{p})\mathbf{V} \tag{13}$$

since $\mathbf{V}^T\mathbf{1} = 0$. The asymptotic normal approximation of the ilr transform is then

$$\text{ilr}(\mathbf{p}) \to \mathcal{N}\left(\text{ilr}(n\mathbf{p}), n^{-1}\mathbf{V}^T\text{diag}(1/\mathbf{p})\mathbf{V}\right). \tag{14}$$

Given the particular form of the variance-covariance matrix in ilr space, we derive the variance-covariance matrix in clr space using the projection matrix $\mathbf{V}$ mapping between the two spaces. Using

the properties of linear algebra, variance, and the special form of $\mathbf{V}$, we see that

$$\begin{aligned}
\text{var}[\text{clr}(\mathbf{p})] &= \mathbf{V}\,\text{var}[\text{ilr}(\mathbf{p})]\mathbf{V}^T & (15)\\
&= n^{-1}\mathbf{V}\mathbf{V}^T\text{diag}(1/\mathbf{p})\mathbf{V}\mathbf{V}^T & (16)\\
&= n^{-1}\left(\mathbf{I}_D - D^{-1}\mathbf{1}\mathbf{1}^T\right)\text{diag}(1/\mathbf{p})\left(\mathbf{I}_D - D^{-1}\mathbf{1}\mathbf{1}^T\right)^T & (17)\\
&= n^{-1}\left(\text{diag}(1/\sqrt{\mathbf{p}}) - D^{-1}\mathbf{1}\mathbf{1}^T\text{diag}(1/\sqrt{\mathbf{p}})\right)\left(\text{diag}(1/\sqrt{\mathbf{p}}) - D^{-1}\mathbf{1}\mathbf{1}^T\text{diag}(1/\sqrt{\mathbf{p}})\right)^T & (18)\\
&= n^{-1}\left[\text{diag}(1/\mathbf{p}) - 2D^{-1}\text{diag}(1/\sqrt{\mathbf{p}})\mathbf{1}\mathbf{1}^T\text{diag}(1/\sqrt{\mathbf{p}}) + D^{-2}\mathbf{1}\mathbf{1}^T\text{diag}(1/\mathbf{p})\mathbf{1}\mathbf{1}^T\right]. & (19)
\end{aligned}$$

This describes the full $D \times D$ asymptotic variance-covariance matrix of the clr-transformed proportions. The sampling variance of the ith component is

$$\text{var}[\text{clr}(p_i)] = \frac{1}{n}\left[\frac{1}{p_i} - \frac{2}{Dp_i} + \frac{1}{D^2}\sum_{j=1}^{D}\frac{1}{p_j}\right]. \tag{20}$$

For observed data, the maximum likelihood estimate of the proportion can be substituted for the true value to obtain an estimate of the variance

$$\widehat{\text{var}[\text{clr}(p_i)]} = \frac{1}{n}\left[\frac{1}{\hat{p}_i} - \frac{2}{D\hat{p}_i} + \frac{1}{D^2}\sum_{j=1}^{D}\frac{1}{\hat{p}_j}\right]. \tag{21}$$

Note that the variance is a function of the total number of counts, number of components, and the proportions for each component, but the projection matrix $\mathbf{V}$ cancels out. This gives a simple formula for the sampling variance of each value in clr space.

An alternative derivation

During revision, we found a simpler derivation of the same result that avoids the ilr transform. Using vector notation for the proportions, we can restate the clr function from Equation (1) and as

$$\text{clr}(\mathbf{p}) = \left(\mathbf{I}_D - \frac{1}{D}\mathbf{1}\mathbf{1}^T\right)\log(\mathbf{p}). \tag{22}$$

Applying the delta method directly to to this transform, we see that

$$\begin{aligned}
\frac{\partial\,\text{clr}(\mathbf{p})}{\partial \mathbf{p}} &= \left(\mathbf{I}_D - \frac{1}{D}\mathbf{1}\mathbf{1}^T\right)\frac{\partial \log(\mathbf{p})}{\partial \mathbf{p}} & (23)\\
&= \left(\mathbf{I}_D - \frac{1}{D}\mathbf{1}\mathbf{1}^T\right)\text{diag}(1/\mathbf{p}) & (24)
\end{aligned}$$

so that

$$\text{var}[\text{clr}(\mathbf{p})] = n^{-1}\left(\mathbf{I}_D - \frac{1}{D}\mathbf{1}\mathbf{1}^T\right)\text{diag}(1/\mathbf{p})\left(\mathbf{I}_D - \frac{1}{D}\mathbf{1}\mathbf{1}^T\right)^T, \tag{25}$$

which is equivalent to Equation (17).

11. The derivation of Equation (18) appears to be based on expressions for conditional and marginal variance. More explicit detail is needed regarding:
- The derivation and estimation of the parameter τ.
- Whether $\tau = 1$ reduces the model to a multinomial special case. (noting that if τ=1, then n=1 by the formula.)
- Clarification on whether x in Equation (16) corresponds to the count vector c used elsewhere.

The x term refers to counts, but we have replaced it with $\mathbf{c}$ for clarity. We have substantially expanded the Supplementary Methods to address these questions:

1.2.2 Asymptotic normal approximation to a Dirichlet-multinomial

The Dirichlet-multinomial (DMN) distribution is a more flexible model of multi-class count data that generalizes the multinomial distribution. While the multinomial distribution assumes the underlying proportions are fixed, in the DMN the proportions are random variables drawn from a Dirichlet distribution. Counts from a DMN model can be drawn according to

$$\mathbf{c} \sim \mathit{Multinomial}(n, \mathbf{p}) \tag{26}$$
$$\mathbf{p} \sim \mathit{Dirichlet}(\boldsymbol{\alpha}) \tag{27}$$

where $\boldsymbol{\alpha}$ is the vector of parameters for Dirichlet distribution so that the expected proportion of category i is α_i/α_0 where $\alpha_0 = \sum_k \alpha_k$. Following standard results for the DMN, the sampling variance of the observed counts is

$$\mathrm{var}(\mathbf{c}) = n\left[\mathrm{diag}(\mathbf{p}) - \mathbf{p}\mathbf{p}^T\right]\left(\frac{n+\alpha_0}{1+\alpha_0}\right). \tag{28}$$

Letting $\tau = (n+\alpha_0)/(1+\alpha_0)$, then

$$\mathrm{var}(\mathbf{c}) = n\left[\mathrm{diag}(\mathbf{p}) - \mathbf{p}\mathbf{p}^T\right]\tau. \tag{29}$$

Based on this, $\tau = 1$ corresponds to the multinomial distribution, and τ can be interpreted as the overdispersion parameter that increases the variance of the counts while keeping their expected values unchanged.

Since the overdispersion τ is a scalar parameter shared by all categories, the derivation from the previous section can be adapted to show that the proportions are asymptotically normal according to

$$\hat{\mathbf{p}} \xrightarrow{d} \mathcal{N}\left(\mathbf{p}, \frac{\tau}{n}\left[\mathrm{diag}(\mathbf{p}) - \mathbf{p}\mathbf{p}^T\right]\right), \tag{30}$$

so that the asymptotic variance of the clr-transformed proportions is

$$\widehat{\mathrm{var}[\mathrm{clr}(p_i)]} = \frac{\hat{\tau}}{n}\left[\frac{1}{\hat{p}_i} - \frac{2}{D\hat{p}_i} + \frac{1}{D^2}\sum_{j=1}^{D}\frac{1}{\hat{p}_j}\right]. \tag{31}$$

This approximation is quite accurate even for small fractions when a sufficient number of total cells are observed (**Figure S10**).

On the special form of the variance in DMN model

In the DMN model the overdispersion parameter, τ, is constant across samples and components. Moreover, the variance of the clr-transformed proportions is a multiplicative function of the overdispersion parameter. While this can have some drawbacks compared to more flexible models where overdispersion can vary across samples and components, the form of the variance of the clr-transformed proportions allows a uniquely simple way to model it with a weighted regression model.

In the case of heteroskedastic errors (see Section 1.3.1 below), fitting a regression weighting samples proportional to the inverse of the variance for each sample gives consistent estimates of the coefficients (Carroll and Ruppert, 2017). Considering a single response variable, let w_k indicate the relative weight and y_k be the observed value of the kth sample and so that

$$w_k \propto 1/\text{var}(y_k). \tag{32}$$

In the case of clr-transformed proportions, let $w_{i,k}$ be the weight and $v_{i,k}$ be the sample variance of the ith component for the kth sample, so that

$$w_{i,k} \propto 1/v_{i,k}. \tag{33}$$

Since the weights must be *proportional* to the inverse variance, only the *relative* variances matter. If the value of $\hat{\tau}$ were estimated from the data, it would cancel out in computing the relative weights. It follows that the relative weights are thus invariant to the value of $\hat{\tau}$. Therefore, estimates of the coefficients and their covariance, as well as hypothesis testing does not depend on the value of $\hat{\tau}$, and it does not need to be estimated from the data.

Combining the single overdispersion term of the DMN model shared across samples with the scale-invariant weighted regression removes the need to estimate $\hat{\tau}$ from the data. Of course, the *true* value of τ affects the observed data, coefficient estimates, etc., but these are invariant to the *estimated* value of τ.

12. Single-cell data often exhibit zero-inflation. The current manuscript does not address this issue. The authors should discuss potential adjustments or methodological extensions that account for zero-inflated distributions.

Single cell gene expression data often have many genes with zero counts. Despite the many observations with zero values, recent work has argued that these can be explained by a low total read count and small rate under a Poisson model, rather than a "zero-inflated" model with a separate process that generates zero counts [5,6]. This distinction is important, because modeling a zero-inflated process is complicated, while counts with a high rate of zero's under a standard Poisson, multinomial, or Dirichlet multinomial due to a low total count or small rate parameter can be modeled with the CLR transform using a pseudocount. In the context of cell type composition, zero counts are much less common than in single cell gene expression data in our experience.

Using the CLR transform without precision weighting gives equal weight to all samples. In this case, the samples with zero counts have equal weights as all other samples in he dataset. However, `crumblr` uses a precision weighted regression that models the sampling variance of the CLR transformed proportions. Since the pseudocount is small, samples with zero observed values receive the smallest relative weights and contribute less to the model fit than other samples.

13. The manuscript presents four case studies; however, none provide sufficient details regarding the data processing workflow (e.g., cell annotation procedures) or in-depth interpretation of the biological results.
- A detailed description of the single-cell preprocessing steps is vital, as these can substantially influence the analysis outcomes.
- The inclusion of appropriate biological interpretations will help demonstrate the method's practical impact.

We thank the Reviewer for this feedback. We have added a new section to the Methods to clarify:

Analysis of compositional data from single cell datasets
Single cell transcriptomics data was obtained for peripheral blood mononuclear cells [7], T cells following tuberculosis infection [8], bone metastases from prostate cancer [9], and blood following SARS-CoV-2 infection [10]. For all datasets, no additional processing was performed and cell type annotations provided by the original studies were used in our analysis.

We have added additional interpretation of the biological results below.

14. In Case Study 1, the authors report a decrease in the proportion of naive CD8+ T cells with age (Fig. 3D).
- Is this finding biologically significant?
- I recommend citing relevant literature to contextualize this observation.

Furthermore, the study's results regarding different cell types (e.g., central memory CD4+ T cells and regulatory T cells) should be discussed in terms of their biological relevance.

We thank the Reviewer for this feedback. We have expanded text in the Results to address this concern.

T cell populations are dynamic and known to change over the course of aging; our findings are consistent with previous studies. Thymic involution associated with aging results in decreased generation of naive T cells and, in particular, causes a marked decrease in naive $CD8^+$ T cells in peripheral blood, a modest decrease in naive $CD4^+$ T cells, and a compensatory increase in memory T cells [11,12]. Indeed, we observe a large decrease in naive $CD8^+$ αβ T cells, a minimal change in naive $CD4^+$ αβ T cells, and an increase in effector memory CD8+ αβ T cells and $CD4^+$ αβ cytotoxic memory T cells. The loss of $CD8^+$ T cells with age is a key factor in immunosenescence, resulting in a reduced ability to fight off infections and a reduced response to vaccination [11–13].

15. In Case Study 2, the observed decrease in Th1 or Th17 clusters among TB-infected individuals needs a discussion on its biological implications.

We thank the Reviewer for this feedback. We have expanded text in the Results to address this concern.

The finding of a decreased $CD4^+$ T_h17 frequency was then replicated in independent cohorts [8,14]. Although a low $CD4^+$ T_h17 frequency may precede progression to TB and thus increase susceptibility [8], here we identify 3 additional T cell clusters whose frequency covaries with $CD4^+$ T_h17 and also shows decreased frequency in TB progressors. In addition, we observe an increase in the frequency of $CD4^+$ cytotoxic T cells, consistent with increased cytotoxicity of $CD4^+$ T cells following active TB [15].

16. In Case Study 3, clarification is required on what “Disease Status” refers to (e.g., TNM stage) and the biological significance of pericyte upregulation in tumors.

> We thank the Reviewer for this feedback. We have updated the text in the Results to address this concern.
>
> Variance partitioning analysis shows substantial variation in cell composition across patients, disease status of each biospecimen (categories: tumor, involved bone marrow, distal bone marrow, benign), and status of the patient (categories: cancer, non-cancer) (**Fig. 5A**)
>
> Pericytes reside on the outside of capillaries, regulate blood flow, and play a key role in tumor vascularization and microenvironment, so an increase in frequency is expected in tumor compared to involved bone marrow [9,16].

While the Crumblr framework offers an innovative approach to analyzing differences in cellular composition, the manuscript requires substantial revisions to address the methodological and biological concerns detailed above. Enhancing the clarity of model derivations, providing comprehensive data processing details, and contextualizing the biological findings will considerably improve the robustness and overall impact of the study.

I look forward to your revised submission addressing these points.

Sincerely

Reviewer #1 (Remarks on code availability):

I evaluated the provided R package and associated code. Overall, the package installs correctly and the core code functions as expected, with no major issues during installation or execution. However, the additional analysis scripts rely primarily on the code itself for guidance. Due to the absence of certain datasets, I was unable to fully execute some of the supplementary analyses, which limits direct validation of all the results presented in the paper.

It would be helpful if the authors could provide a more comprehensive README file or additional documentation that includes detailed instructions on installing, running the application, and performing the associated analyses.

> We thank the Reviewer for this valuable feedback. We have updated https://github.com/GabrielHoffman/crumblr_analysis so now each dataset has its down README.md file describing how to run the analysis.

Reviewer #2 (Remarks to the Author):

I co-reviewed this manuscript with one of the reviewers who provided the listed reports. This is part of the Nature Communications initiative to facilitate training in peer review and to provide appropriate recognition for Early Career Researchers who co-review manuscripts.

We thank the Reviewer for their feedback.

Reviewer #3 (Remarks to the Author):

This manuscript introduces crumblr, a novel statistical framework for differential composition analysis of single-cell data, addressing the critical challenge of accurately identifying changes in cell type frequencies while accounting for variable measurement precision. By integrating centered log-ratio transformation with precision-weighted linear models and incorporating multivariate testing for hierarchical cell relationships, the authors propose a potentially powerful and flexible tool. The manuscript presents compelling simulation studies suggesting improved performance over existing methods and demonstrates practical utility through applications to diverse biological datasets. This work represents a valuable methodological advancement for the single-cell field.

While the manuscript is a strong contribution, the following points could be addressed to further enhance its clarity and impact:

Specific Points for Consideration:

1. Handling of Zero Counts: The manuscript should explicitly detail the strategy employed to handle zero counts for cell types within samples, which is a known issue for CLR-based methods. Was a pseudocount implemented? If so, justification for the chosen value and an analysis of sensitivity to this choice should be provided. While Figure 1B mentions the approximation works "when at least two counts are observed", the practical implementation for zero values needs clarification.

> We thank the Reviewer for this feedback. We now describe the pseudocount included in the crumblr analysis and demonstrate through simulations that the results are robust to the particular value selected. We have added the following to the Methods and Supplementary Figures:
>
> Additionally, a pseudocount value of 0.5 is added to all counts, allowing crumblr to handle zero counts. Performance is robust to changes in this parameter across a wide range of values, and the default value retains high power and controls the false positive rate in simulations (**Fig S13)**. We note that the defaults for both of these values were set during the software's development, not as a result of these simulations.

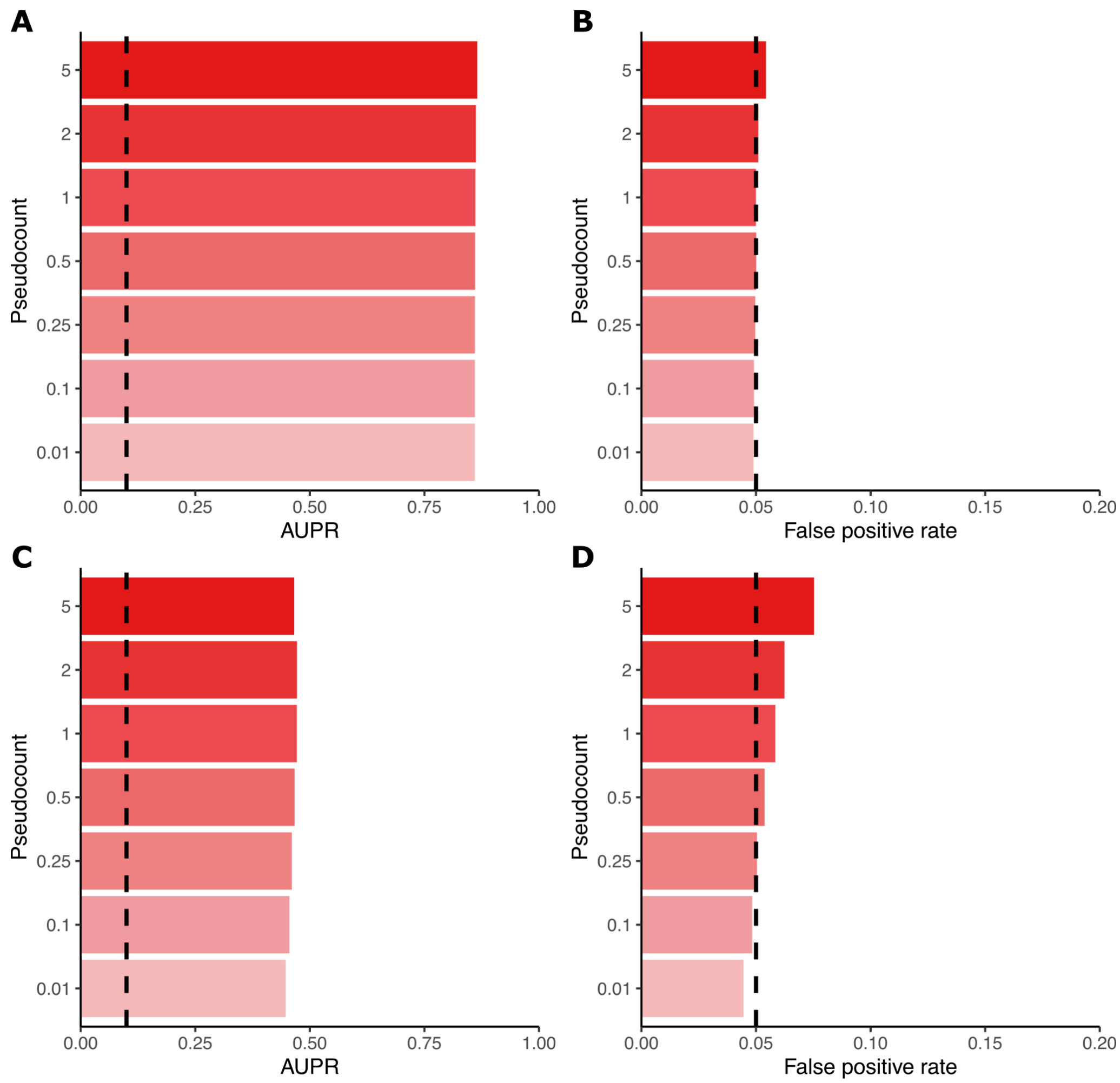

Figure S13: Sensitivity analysis: pseudocount. Simulations from Fig 2A were repeated, changing the pseudocount value for crumblr. Results are shown for 10k simulations. A,B) AUPR and false positive rate. C,D) AUPR and false positive rate for simulations, reducing the total number of cells from 2000 to 200 to consider conditions where the pseudocount has a larger effect on results.

2. Overdispersion Parameter (τ): The rationale for setting the Dirichlet-multinomial overdispersion parameter τ to 1, based on the invariance of coefficient estimates to weight scaling, requires further justification. While coefficient estimates may be invariant, does fixing τ=1 potentially impact the accuracy of the variance estimation itself (used for precision weights), particularly when true overdispersion might vary substantially between cell types or samples? Please elaborate on the implications of not estimating or incorporating τ.

We thank the Review for this question. Tau is an important parameter in simulating the dataset and fitting the DMN model. However, due to the special form of the DMN model and the weight regression we use here, it is not necessary to estimate τ from the data. We have substantially expanded the Supplementary Methods to clarify this point.

1.2.2 Asymptotic normal approximation to a Dirichlet-multinomial

The Dirichlet-multinomial (DMN) distribution is a more flexible model of multi-class count data that generalizes the multinomial distribution. While the multinomial distribution assumes the underlying proportions are fixed, in the DMN the proportions are random variables drawn from a Dirichlet distribution. Counts from a DMN model can be drawn according to

$$\mathbf{c} \sim \mathit{Multinomial}(n, \mathbf{p}) \tag{26}$$

$$\mathbf{p} \sim \mathit{Dirichlet}(\boldsymbol{\alpha}) \tag{27}$$

where $\boldsymbol{\alpha}$ is the vector of parameters for Dirichlet distribution so that the expected proportion of category i is α_i/α_0 where $\alpha_0 = \sum_k \alpha_k$. Following standard results for the DMN, the sampling variance of the observed counts is

$$\text{var}(\mathbf{c}) = n\left[\text{diag}(\mathbf{p}) - \mathbf{p}\mathbf{p}^T\right]\left(\frac{n+\alpha_0}{1+\alpha_0}\right). \tag{28}$$

Letting $\tau = (n+\alpha_0)/(1+\alpha_0)$, then

$$\text{var}(\mathbf{c}) = n\left[\text{diag}(\mathbf{p}) - \mathbf{p}\mathbf{p}^T\right]\tau. \tag{29}$$

Based on this, $\tau = 1$ corresponds to the multinomial distribution, and τ can be interpreted as the overdispersion parameter that increases the variance of the counts while keeping their expected values unchanged.

Since the overdispersion τ is a scalar parameter shared by all categories, the derivation from the previous section can be adapted to show that the proportions are asymptotically normal according to

$$\hat{\mathbf{p}} \xrightarrow{d} \mathcal{N}\left(\mathbf{p}, \frac{\tau}{n}\left[\text{diag}(\mathbf{p}) - \mathbf{p}\mathbf{p}^T\right]\right), \tag{30}$$

so that the asymptotic variance of the clr-transformed proportions is

$$\widehat{\text{var}[\text{clr}(p_i)]} = \frac{\hat{\tau}}{n}\left[\frac{1}{\hat{p}_i} - \frac{2}{D\hat{p}_i} + \frac{1}{D^2}\sum_{j=1}^{D}\frac{1}{\hat{p}_j}\right]. \tag{31}$$

This approximation is quite accurate even for small fractions when a sufficient number of total cells are observed (**Figure S10**).

On the special form of the variance in DMN model

In the DMN model the overdispersion parameter, τ, is constant across samples and components. Moreover, the variance of the clr-transformed proportions is a multiplicative function of the overdispersion parameter. While this can have some drawbacks compared to more flexible models where overdispersion can vary across samples and components, the form of the variance of the clr-transformed proportions allows a uniquely simple way to model it with a weighted regression model.

In the case of heteroskedastic errors (see Section 1.3.1 below), fitting a regression weighting samples proportional to the inverse of the variance for each sample gives consistent estimates of the coefficients (Carroll and Ruppert, 2017). Considering a single response variable, let w_k indicate the relative weight and y_k be the observed value of the kth sample and so that

$$w_k \propto 1/\mathrm{var}(y_k). \tag{32}$$

In the case of clr-transformed proportions, let $w_{i,k}$ be the weight and $v_{i,k}$ be the sample variance of the ith component for the kth sample, so that

$$w_{i,k} \propto 1/v_{i,k}. \tag{33}$$

Since the weights must be *proportional* to the inverse variance, only the *relative* variances matter. If the value of $\hat{\tau}$ were estimated from the data, it would cancel out in computing the relative weights. It follows that the relative weights are thus invariant to the value of $\hat{\tau}$. Therefore, estimates of the coefficients and their covariance, as well as hypothesis testing does not depend on the value of $\hat{\tau}$, and it does not need to be estimated from the data.

Combining the single overdispersion term of the DMN model shared across samples with the scale-invariant weighted regression removes the need to estimate $\hat{\tau}$ from the data. Of course, the *true* value of τ affects the observed data, coefficient estimates, etc., but these are invariant to the *estimated* value of τ.

3. Weight Regularization: The manuscript describes regularization of precision weights to mitigate the influence of samples with extremely high weights. Please provide further rationale for the specific regularization approach (limiting the max/quantile ratio) and the selection of default parameters (ratio=5, quantile=5%). A sensitivity analysis exploring the impact of these parameters on results would be informative.

We thank the Reviewer for this feedback. We now demonstrate through simulations that the results are robust to the particular value selected. We have added the following to the Methods and Supplementary Figures:

Performance is robust to changes in this parameter across a wide range of values, and default `max.ratio=5` retains high power and controls the false positive rate in simulations (**Fig S12)**. In addition, a pseudocount value of 0.5 is added to all counts so that crumblr can handle zero counts. Performance is robust to changes in this parameter across a wide range of values, and the default value retains high power and controls the false positive rate in simulations (**Fig S13)**. We note that the defaults for both of these values were set during development of the software, and not as a result of these simulations.

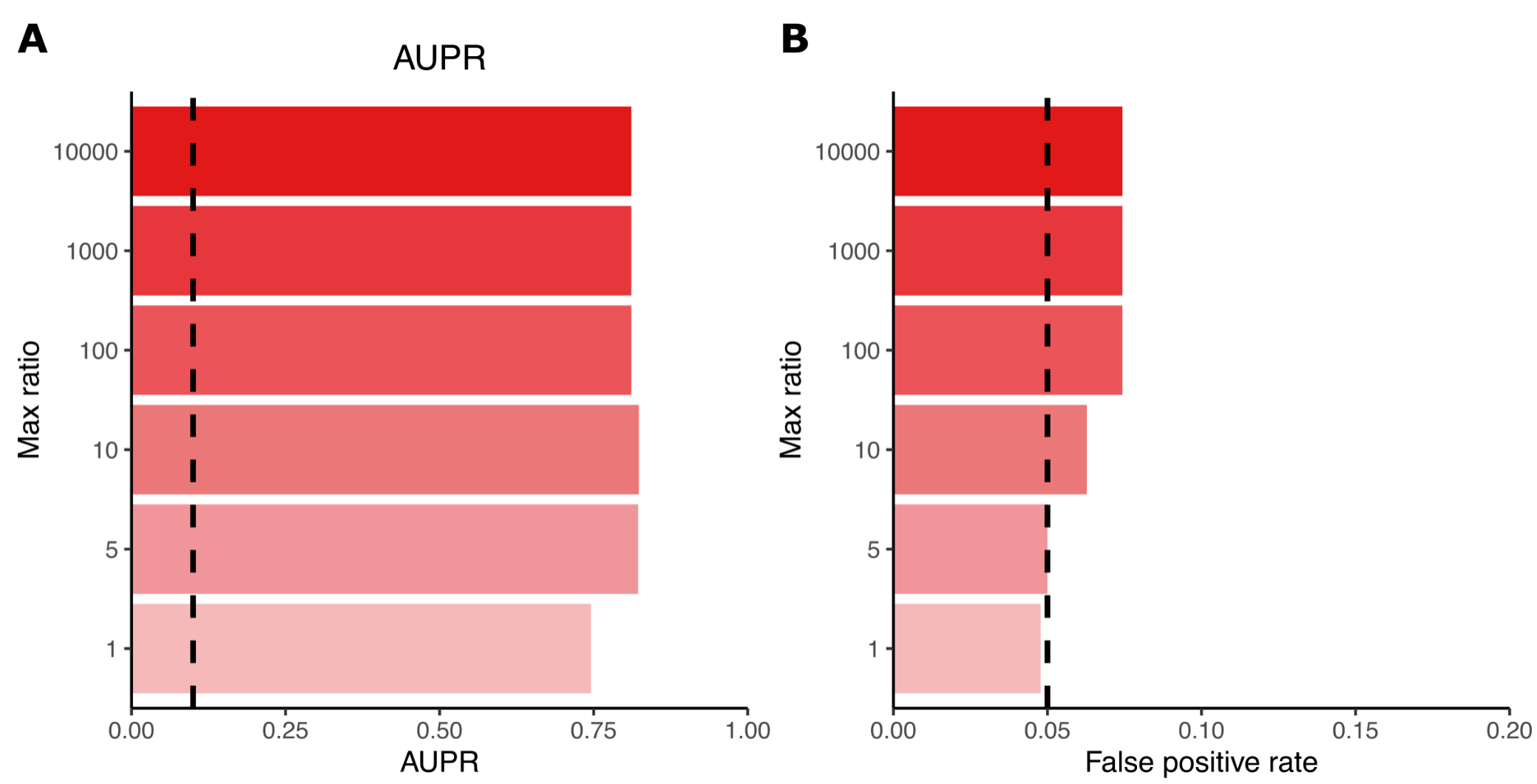

Figure S12: Sensitivity analysis: weight regularization. Simulations from Fig 2A were repeated changing the weight regularization parameter value for crumblr. This parameter caps the ratio between the maximum value and specified quantile value (default of 5%). A) AUPR and B) false positive rate are shown for 10k simulations. With max.ratio set to 1, there is very little variation in the precision weights and the method behaves like standard CLR. With large values for max.ratio, stochastic variation in the precision weights can give very high weights to a small subset of samples. This can increase the false positive rate and underscores the need to regularize the weights.

4. Empirical Null for Multivariate Test: The extension of the Lin-Sullivan test using an empirical null distribution derived from Wishart sampling is an interesting approach to control false positives in small samples. Clarification is needed on how the degrees of freedom (v) for the Wishart sampling distribution are determined, particularly within the context of linear mixed models where degrees of freedom estimation can be complex.

We thank the Reviewer for this question. We have added the following text to the Supplementary Methods:

Residual degrees of freedom

In the case of a linear model, the residual degrees of freedom, ν, is computed from the sample size and the number of coefficients estimated. In the case of the a linear mixed model, the form of the residual degrees of freedom is more complicated. We use an approximation we developed previously (Hoffman et al., 2024). See Supplementary Methods Section 2.3.1 of that work for the full derivation. It is implemented in `variancePartition::rdf.merMod()` and computes the residuals degrees of freedom from a linear mixed model fit with `lme4::lmer()`.

5. Scalability and Performance: While the manuscript indicates favorable computational performance compared to scCODA, a more formal analysis of computational complexity would be beneficial. How does the runtime scale with the number of samples (n), cell types (D), covariates (c), and the complexity of random effects structures? Is the multivariate testing step computationally intensive compared to the univariate analyses?

We thank the Reviewer for this feedback. We have added details about the computational complexity in the Methods:

Time complexity

Evaluating the CLR transform and the precision weights with crumblr is linear in the number of samples, n, and cell types, D. It is very fast and takes < 1 second for 1000 samples and 100 cell types. Fixed effect linear regression with c covariates, is $\mathcal{O}(n^2c + c^3)$ and requires just a few seconds even for large datasets. Linear mixed models are linear in n, but the complexity as a function of c is more complicated because estimation uses sparse matrix encoding of random effects and uses iterative methods to maximize the log-likelihood [17]. Combining results across D cell types using the Lin-Sullivan statistic is $\mathcal{O}(D^3)$. Using the Monte Carlo sampling, combining effects along a tree of 30 leaves takes < 30 seconds.

6. Interpretation of Hierarchical Tests: When multivariate tests on internal nodes of the cell hierarchy yield significant results (e.g., Figures 4C, 5C), guidance on the biological interpretation is needed. Does significance indicate a coordinated change across child nodes, an average effect, or primarily reflect the strongest effect among children? How should researchers relate parent node significance back to individual cell types?

We thank the Reviewer for this feedback. We have added a new section to the Methods:

Multivariate hierarchical testing with `treeTest()`

In the field of single-cell transcriptomics, the number of cell types analysed varies widely across studies. For example, Mathys, et al.[1] perform analysis on 12 major cell types and 54 higher resolution clusters from human prefrontal cortex to identify gene expression changes in Alzheimer's disease, while the BRAIN Initiative Cell Census Network identified 31 cell superclusters, 461 clusters, and 3313 subclusters across multiple brain regions[2]. In the brain, there is vast diversity in neuronal subtypes, but much less diversity in other cell types[2]. In practice, the number of cell type clusters used is determined by the tissue, the number of cells observed, and the biological question.

Yet in analyses of differential cell type composition, the number of cell clusters used can affect the analysis due to the tradeoff between resolution and statistical power. While increasing the number of cell clusters enhances cell type resolution and the biological specificity of each cluster, the frequency of each higher-resolution cluster decreases. All analysis methods we considered suffer from a loss in power as the cell type frequency decreases. This fundamental tradeoff leaves the analyst to select the appropriate number of cell types to address their biological question.

Instead of selecting this number at the beginning of a project, the `treeTest()` analysis in the `crumblr` framework performs tests of differential abundance at multiple levels of cell type resolution. Given a hierarchical clustering of cell types, the method performs a multivariate hypothesis test on each internal node in the hierarchy. Given D total cell clusters at the leaves of this hierarchy, consider an internal node ℓ with d_ℓ descendant leaves. Letting $\hat{\vec{\beta}}_\ell$ be the vector of estimated coefficients from descendant leaves of node ℓ, we want to test the null hypothesis that the mean effect size is zero. Since high-resolution cell clusters often have frequencies that covary, modeling covariance in the estimated coefficients increases power to reject the null hypothesis

while controlling the false positive rate. This test of the mean effect of descendant leaf nodes is repeated for each internal node using the empirical Lin-Sullivan test.

By testing at internal nodes, this approach can accumulate power across multiple leaves where testing at a single leaf is underpowered. Rejecting the null hypothesis at an internal node where few or no leaves show a significant signal can indicate that high-resolution clustering caused loss of power at the leaves, which is then 'rescued' by the multilevel analysis. However, note that a strong signal in one leaf can drive the result at an internal node where all other leaves have small effects, as we see in **Fig. 3B**. We also note that the results depend on the hierarchical clustering used.

7. Simulation Comparisons: The comparison in simulations (Fig 2A) includes methods based on transformations (logit, arcsin-sqrt) suitable for proportions bounded [0,1], whereas CLR yields unbounded values. Please comment on the fairness of this comparison given the different data scales and underlying assumptions. Furthermore, discussion regarding how crumblr compares to other count-based compositional data analysis methods beyond those included (e.g., ALDEx2, ANCOM-BC) would strengthen the comparative analysis.

We thank the Reviewer for this feedback. We have added the following text to the Methods:

Comparison with other methods

Statistical testing of differences in cell type frequency typically use models based on frequencies, counts, normal approximations, or non-parametrics. Here we summarize some of the assumptions and limitations of models in our comparison. Binomial models for frequency data can be fit with a generalized linear model (GLM), but do not model overdispersion or heteroskedasticity due to differences in total counts per sample. Beta-binomial models add an overdispersion term. Poisson models of counts can be fit with a GLM, but do not model overdispersion. Negative binomial models extend the Poisson model to accommodate overdispersion. DESeq2 and edgeR are negative binomial models designed for RNA-seq and use a Bayesian method to borrow information across features (i.e. either genes or cell clusters). However, this information borrowing was not designed for the relatively small number of cell clusters and can fail in the case of DESeq2 (see **Fig S6**). The multinomial and Dirichlet-multinomial distributions are often used to simulate count data. But performing parameter estimation and hypothesis testing for complex study designs with covariates is challenging.

For a normal approximation, the count data is transformed so that the sampling variance of each observation is approximately equal and the new values can be fit with a linear

model. Transformations include CLR, asin, log, logit, and identity (where regression is performed on the fractions directly).

The crumblr method uses a more sophisticated normal approximation using weighted regression based on the Dirichlet-multinomial distribution. This allows crumblr to retail high power and speed, while accommodating random effects and multivariate testing.

Tests of differential frequencies are also important in the microbiome field. For example, ANCOM-BC is designed for sparse microbiome datasets, models uncertainty in the observed counts and uses a bias correction as an offset in the regression [42]. This bias correction accounts for variation in how well observed cell proportions from a small tissue sample are representative of the true cell proportion of the entire tissue. ALDEx2 models uncertainty in the observed counts by using a 'multiple imputation' approach [43]. A Dirichlet model is first used to fit the observed data. Multiple sample datasets are drawn from this distribution, the CLR transform is applied and a regression analysis is performed on each one. Summary statistics are then computed across the Monte Carlo samples.

8. Modeling Non-Linear Effects: The aging dataset analysis (Fig 3D) raises an interesting point about linearity. While crumblr supported a linear trend where a standard model might suggest non-linearity, could the precision weighting potentially mask true biological non-linearities by down-weighting samples where frequencies are low (often at extremes of a biological range)? Please discuss how users might investigate or model potential non-linear associations within the crumblr framework.

We thank the Reviewer for this feedback. In this case, we observe a non-linear association between age and composition of $CD8^+$ αβ T cells when the measurement precision is ignored using the standard CLR approach. Using crumblr to model the variability in measurement precision, only the linear association is supported. Here, modeling the precision weights with crumblr avoids a false positive finding here. An important question is whether, crumblr could hide a non-linear association to produce a false negative.

In exploratory research like in genomics, control of the false positive rate is the top priority. In simulations, crumblr shows control of the false positive rate in a range of conditions, but these involve linear effects. A more rigorous consideration of non-linear associations would require additional real datasets and extensive analysis that is beyond the scope of the current work.

Reviewer #3 (Remarks on code availability):

The provided vignette offers a clear, step-by-step demonstration of the crumblr package workflow using an example dataset, effectively illustrating its integration with established Bioconductor tools like variancePartition and limma/dream. The code appears well-structured and covers key functionalities including data transformation, variance decomposition, PCA, differential testing, and the novel multivariate tree analysis. To further enhance usability, consider adding more detailed explanations within the vignette regarding the interpretation of intermediate objects (like cobj) and the rationale for specific functions (e.g., standardize()). Additionally, explicitly showing or referencing the method used to generate the input hierarchy (hcl) for treeTest, and providing brief guidance on potential scalability considerations or parameter tuning for larger, more complex datasets would be beneficial for prospective users.

> We thank the Reviewer for this valuable feedback. We have improved the documentation in crumblr v0.99.22:
>
> - Hierarchical clustering
>   - Using crumblr in practice
>   - Integration with dreamlet / SingleCellExperiment
> - Interpretation of testing internal nodes
>   - Interpretation
> - Object returned by `crumblr()`
>   - Processed data
> - Standardize
>   - Homoscedastic data
> - Computational scaling, complex study designs and parameter tuning
>   - Considerations

References

1. Mathys, H. *et al.* Single-cell multiregion dissection of Alzheimer's disease. *Nature* **632**, 858–868 (2024).
2. Siletti, K. *et al.* Transcriptomic diversity of cell types across the adult human brain. *Science* **382**, eadd7046 (2023).
3. Liu, Y. *et al.* ACAT: A fast and powerful p value combination method for rare-variant analysis in sequencing studies. *Am. J. Hum. Genet.* **104**, 410–421 (2019).
4. Wilson, D. J. The harmonic mean p-value for combining dependent tests. *Proc. Natl. Acad. Sci. U. S. A.* **116**, 1195–1200 (2019).
5. Sarkar, A. & Stephens, M. Separating measurement and expression models clarifies confusion in single-cell RNA sequencing analysis. *Nat. Genet.* **53**, 770–777 (2021).
6. Svensson, V. Droplet scRNA-seq is not zero-inflated. *Nat. Biotechnol.* **38**, 147–150 (2020).
7. Yazar, S. *et al.* Single-cell eQTL mapping identifies cell type-specific genetic control of autoimmune disease. *Science* **376**, eabf3041 (2022).
8. Nathan, A. *et al.* Multimodally profiling memory T cells from a tuberculosis cohort identifies cell state associations with demographics, environment and disease. *Nat. Immunol.* **22**, 781–793 (2021).
9. Kfoury, Y. *et al.* Human prostate cancer bone metastases have an actionable immunosuppressive microenvironment. *Cancer Cell* vol. 39 1464–1478.e8 Preprint at https://doi.org/10.1016/j.ccell.2021.09.005 (2021).
10. COMBAT Consortium. A blood atlas of COVID-19 defines hallmarks of disease severity and specificity. *Cell* **185**, 916–938.e58 (2022).
11. Zhang, H., Weyand, C. M. & Goronzy, J. J. Hallmarks of the aging T-cell system. *FEBS J.* **288**, 7123–7142 (2021).
12. Mittelbrunn, M. & Kroemer, G. Hallmarks of T cell aging. *Nat. Immunol.* **22**, 687–698 (2021).
13. Sun, L., Su, Y., Jiao, A., Wang, X. & Zhang, B. T cells in health and disease. *Signal*

Transduct. Target. Ther. **8**, 235 (2023).

14. Sun, M. *et al.* Specific CD4+ T cell phenotypes associate with bacterial control in people who ‘resist’ infection with Mycobacterium tuberculosis. *Nat. Immunol.* **25**, 1411–1421 (2024).
15. Flores-Gonzalez, J. *et al.* The presence of cytotoxic CD4 and exhausted-like CD8+ T-cells is a signature of active tuberculosis. *Biochim. Biophys. Acta Mol. Basis Dis.* **1870**, 167219 (2024).
16. Jiang, Z. *et al.* Pericytes in the tumor microenvironment. *Cancer Lett.* **556**, 216074 (2023).
17. Bates, D., Mächler, M., Bolker, B. & Walker, S. Fitting Linear Mixed-Effects Models Using lme4. *J. Stat. Softw.* **67**, (2015).

Response to reviewers.

We thank the reviewers for their careful reading of our manuscript and pointing out cases where our mathematical notation was imprecise. Our responses are shown below in black, indented text. Changes to the manuscript are shown in red text.

Reviewer #1 (Remarks to the Author):

The authors have addressed many of the earlier concerns, and the paper is substantially improved in clarity, scope, and empirical evaluation. However, I have identified several remaining issues that require attention.

1. There are inconsistencies in the mathematical notation throughout the manuscript that may cause confusion for readers. Estimated statistics should be marked with hat notation (^), while true parameters should not. Specific examples include:

In Formula 56: Σ is described as a "known variance-covariance matrix," but the text subsequently indicates it is estimated through formulas 42-52. If estimated, it should be denoted as $\hat{\Sigma}$.

> We thank the Reviewer for pointing out this issue and acknowledge that the original text was unclear. In the previous section, we describe how to estimate the covariance matrix between coefficient estimates. Here, we develop a test statistic for multivariate hypothesis testing. For ease of presentation we divided the derivation into two parts: 1) (Section 1.3.3) consider the idealized case where the covariance matrix is *known*, and then 2) (Section 1.3.4) extend to the realistic case where the covariance matrix is *estimated*.
>
> In order to clarify our two-part approach we added the text:
>
> **1.3.3 Multivariate hypothesis testing**
>
> Here, we develop a test statistic assuming an idealized case where the covariance matrix between parameter estimates is known. In the next section, we extend this to the realistic case of an estimated covariance matrix.

1.3.4 An adaptation for unknown variance

In real data, the covariance matrix between coefficients must be estimated from the data according to Equations (42) or (52). Here, we extend the test statistic to the case of estimated covariance.

In Formula 14: The term clr(p) should be clr(p̂) since p represents an estimate. Similarly, in Formulas 15, 20, 21, and 25: clr(p) should be clr(p̂) to reflect that these are based on estimated proportions.

We agree. We have added hat notation to

$\mathrm{var}[\mathrm{ilr}(\hat{\mathbf{p}})]$ in Eqn 11,

$\mathrm{ilr}(\hat{\mathbf{p}})$ in Eqn 14

$\mathrm{var}[\mathrm{clr}(\hat{\mathbf{p}})] = \mathbf{V}\,\mathrm{var}[\mathrm{ilr}(\hat{\mathbf{p}})]\mathbf{V}^T$ in Eqn 15,

$\mathrm{var}[\mathrm{clr}(\hat{p}_i)]$ in Eqn 20

$\mathrm{var}\widehat{[\mathrm{clr}(\hat{p}_i)]}$ in Eqn 21

$\mathrm{var}[\mathrm{clr}(\hat{\mathbf{p}})]$ in Eqn 25

$\mathrm{var}\widehat{[\mathrm{clr}(\hat{p}_i)]}$ in Eqn 31

I recommend a comprehensive review of all mathematical expressions to ensure consistent notation distinguishing between parameters and their estimates.

We have also added hat notation to

$\hat{\sigma}_i\hat{\sigma}_j$ in Eqn 38

$\hat{s}$ in Eqns 59, 60, 61

2. Regarding the Dirichlet-multinomial (DMN) dispersion parameter τ and the weighting scheme, several points require clarification:

Based on Formula 33, weights w_{i,k} are proportional to the inverse variance v_{i,k}. Is v_{i,k} equivalent to var[clr(p̂_i)] from Formula 31?

Yes. We have added the following to make this clearer:

In the case of clr-transformed proportions, let $w_{i,k}$ be the weight and $v_{i,k}$ be the sample variance of the ith component for the kth sample computed from Eqn (31), so that

$$w_{i,k} \propto 1/v_{i,k}. \tag{33}$$

The manuscript states that relative weights are invariant to the value of τ̂. Could you explain why this is the case? Are the weights scaled and standardized so that their sum equals 1?
A more detailed explanation of how the weights are derived and normalized would be beneficial.

We thank the Reviewer for raising this issue.

The Gauss-Markov Theorem proves that the ordinary least squares estimator of the regression coefficients is the best linear unbiased estimator (BLUE) for the linear model with equal error variance across all samples. Aitken's Theorem extends this to more general error covariance matrices and shows that the generalized least squares (GLS) estimator is the BLUE. We now include a proof that the weighted least squares estimator and its covariance are invariant to the scale of the weights. Therefore, the results are invariant to the value of $\hat{\tau}$.

We have added to following text:

Page 17:

According to Theorem 4.1 (see Section 4), in a weighted least squares regression model the coefficient estimates and their variances are invariant to the scale of the weights. Therefore, by convention, the weights are scaled to sum to 1.

Page 24:

4 Additional proofs

Theorem 4.1. *In weighted least squares, the coefficient estimates and their variances are invariant to the scale of the weights.*

Consider a response y drawn from a multivariate normal distribution with mean determined by design matrix X of n samples and p variables, coefficients β and error term ε with zero mean. Consider a heteroskedastic model

$$y = X\beta + \varepsilon, \tag{67}$$

where error variance varies across samples, and let

$$\text{var}(\varepsilon_i) = 1/w_i \tag{68}$$

be the *true* error variance for sample i.

First, consider estimating the coefficients using weighted least squares. Using inverse variance weights by setting $W = \text{diag}(\vec{w})$ gives

$$\hat{\beta} \;=\; (X^T W^{-1} X)^{-1} X^T W^{-1} y. \tag{69}$$

Instead of using the *true* weights, consider scaling the weights by c and let β_c indicate that corresponding coefficient estimates. It follows that

$$\begin{aligned}
\hat{\beta}_c &= (X^T (cW)^{-1} X)^{-1} X^T (cW)^{-1} y & (70)\\
&= (X^T c^{-1} W^{-1} X)^{-1} X^T c^{-1} W^{-1} y & (71)\\
&= cc^{-1} (X^T W^{-1} X)^{-1} X^T W^{-1} y & (72)\\
&= (X^T W^{-1} X)^{-1} X^T W^{-1} y & (73)\\
&= \hat{\beta}. & (74)
\end{aligned}$$

Therefore the coefficient estimates are invariant to the scale of the weights.

Now consider the variance of the coefficient estimates. Letting $r = y - X\beta$, then

$$\hat{\sigma}^2 = r^T W^{-1} r/(n-p) \tag{75}$$

so that

$$\begin{aligned} \widehat{\text{var}}(\hat{\beta}) &= \hat{\sigma}^2 (X^T W^{-1} X)^{-1} && (76) \\ &= r^T W^{-1} r (X^T W^{-1} X)^{-1}/(n-p) && (77) \end{aligned}$$

Now consider scaling the weights by c, so that

$$\begin{aligned} \widehat{\text{var}}(\hat{\beta}) &= r^T (cW)^{-1} r (X^T (cW)^{-1} X)^{-1}/(n-p) && (78) \\ &= r^T c^{-1} W^{-1} r (X^T c^{-1} W^{-1} X)^{-1}/(n-p) && (79) \\ &= r^T c^{-1} W^{-1} r c (X^T W^{-1} X)^{-1}/(n-p) && (80) \\ &= r^T W^{-1} r (X^T W^{-1} X)^{-1}/(n-p) && (81) \end{aligned}$$

Therefore, the estimated covariance between coefficient estimates is invariant to the scaling of the weights.

■

3. Following up on my previous Comment 7, I note that var[clr(p̂_i)] from Formula 31 is used as weights for weighted multiple regressions. However, these appear to be only the diagonal entries of the complete variance-covariance matrix var[clr(p̂)]. The off-diagonal entries, representing covariances such as cov(clr(p̂_i), clr(p̂_j)), do not appear to be incorporated into subsequent regressions.

If this interpretation is correct, it suggests that some correlation information inherent in the clr transformation is being discarded. Please clarify:

Whether only diagonal elements are used as weights
The rationale for not incorporating the full covariance structure
Any potential implications of ignoring the off-diagonal covariance terms
Whether this simplification affects the validity or efficiency of the statistical inference

> The Reviewer is correct that the model we proposed here considers the sampling variance of the CLR-transformed proportions, but does not consider the sampling *covariance* between cell types. Our model performs weighted fixed or mixed effects regression under a normal model, and analyses each response (i.e. cell type) separately. Compared to previous work that ignores the heteroskedastic error, we believe this is a substantial contribution.
>
> The prospect of extending the model to consider sampling covariance between responses is interesting. In the case of a fixed effect model, this would involve

modeling the data with a matrix-normal distribution. This type of model is called "seemingly unrelated regressions" or SUR to account for correlated errors. SUR models should have increased statistical efficiency for correlated errors.

Our current approach is a special case of the matrix-normal distribution modeling responses as having independent errors.

Implementing the extension involves a number of challenges:

1) I don't have much intuition about the additional value of the more complex model. In empirical data cell type proportions are often correlated, but this is likely due to the biology of these cell types rather than sampling covariance.
2) The time complexity of the current model is linear in the number of cell types. Modeling the sampling covariance between k cell types would require inverting a k x k matrix, so the time complexity would be cubic in the number of cell types.
3) Extending this approach to mixed effects regression in the framework of a SUR / matrix-normal distribution is challenging. It's not clear if it can take advantage of the lme4 package for fitting linear mixed models, as the current model does. Instead, it might require MCMC

In conclusion, this is an intriguing suggestion but it is beyond the scope of the current work.

Reviewer #1 (Remarks on code availability):

The GitHub link provided in the response to Comment 4 appears to be broken (https://github.com/GabrielHoffman/crumblr_analysis/blob/main/simulations/remaCor_gamma.Rmd). This link returns an error and should be verified and corrected to ensure reproducibility of the results.

We apologize for this. The link works now

While the analysis code on GitHub is detailed and logically organized, I was unable to run it successfully in practice due to data-loading errors. Within the limited time available, I was not able to diagnose and resolve the issue.

I am happy to resolve this given more information.

Reviewer #3 (Remarks to the Author):

Thanks for the very thorough and thoughtful response to my previous comments. I've read through the revised manuscript, and I'm really impressed by the amount of work you put into it. The new simulations, the added details in the methods, and the improved biological context have made the paper much clearer and more convincing. All of my previous concerns have been fully addressed. All in all, this is an excellent piece of work. Congratulations!

We thank the Reviewer for their feedback.

Reviewer #3 (Remarks on code availability):

Yes, I've reviewed the code and the associated documentation. The R package installs without any issues, and the core functions work as described. I was particularly pleased to see the significant improvements made to the documentation following the last review. The new README files for each analysis on the GitHub repository are very helpful, and the updated vignette now provides much clearer instructions on how to run the analyses, interpret the results, and handle things like the hierarchical clustering input for treeTest . These changes have successfully addressed the previous concerns about reproducibility . I'm now confident that the code is a well-documented and valuable resource for the community.

We thank the Reviewer for their feedback

REVIEWERS' COMMENTS

Reviewer #1 (Remarks to the Author):

I appreciate the authors' clarification in their response that only the diagonal elements of the variance–covariance matrix (i.e., var[clr(p_i)]) were used as weights, without incorporating the off-diagonal covariance terms. However, I must respectfully insist that this methodological choice requires explicit documentation in the manuscript itself, not merely in the response letter.This is essential for methodological transparency and reproducibility.

The authors should provide a brief rationale for this simplification. While I understand the authors consider full covariance incorporation to be "out of scope," readers need to understand whether this simplification is made for computational convenience or conceptual reasons.

The authors correctly note in their response that the generalized least squares (GLS) estimator incorporating the full variance-covariance matrix would be the Best Linear Unbiased Estimator (BLUE), and that weighted least squares (WLS) using only diagonal elements represents a special case of GLS. This point actually strengthens my concern: since the theoretical framework already accommodates GLS, and the full var[clr(p)] matrix is available from Formula 31, using the complete covariance structure would be straightforward within the existing framework and would yield more efficient estimates. The fact that the full GLS approach fits naturally into the authors' existing theoretical framework makes the omission of covariance terms more puzzling.

I recommend adding a brief paragraph in the Methods section and/or a note in the Discussion/Limitations section addressing these points. This addition would significantly enhance the methodological rigor and transparency of the work without requiring substantial revisions to the analysis itself.

We appreciate the Review's feedback and we have added a new section to the Methods:

Note on statistical modelling

The model we propose here considers the sampling variance of the CLR-transformed proportions, but does not consider the sampling *covariance* between cell types. The model performs weighted fixed or mixed effects regression under a normal model, and analyses each response (i.e. cell type) separately. It is natural to consider the prospect of modelling this covariance in errors. In the case of a fixed effect model, this would involve modeling the data with a matrix-normal distribution. This type of model is called "seemingly unrelated regressions" or SUR to account for correlated errors (Zellner 1962). SUR models should have increased statistical efficiency for correlated errors. Our current approach is a special case of the matrix-normal distribution modeling responses as having independent errors.

This more sophisticated model is indeed an interesting research question, but its potential value and tractability require further thought. First, in empirical data cell type proportions are indeed often correlated. Yet, this is likely due to the biology of these cell types rather than sampling covariance. More work to understand the importance of the error covariance term in realistic data is needed. Second, modeling the error covariance across D cell type would require inverting a $D \times D$ matrix and require $\mathcal{O}(D^3)$ time. Third, extending a SUR-type model to incorporate random effects is challenging. The *crumblr* model uses standard R packages to fit the regression models for each response, and it is unclear if this extension could be accommodated by existing software.
